# Crewmember microbiome may influence microbial composition of ISS habitable surfaces

**DOI:** 10.1371/journal.pone.0231838

**Published:** 2020-04-29

**Authors:** Aram Avila-Herrera, James Thissen, Camilla Urbaniak, Nicholas A. Be, David J. Smith, Fathi Karouia, Satish Mehta, Kasthuri Venkateswaran, Crystal Jaing

**Affiliations:** 1 Computating Directorate, Lawrence Livermore National Laboratory, Livermore, California, United States of America; 2 Physical and Life Sciences Directorate, Lawrence Livermore National Laboratory, Livermore, California, United States of America; 3 Biotechnology and Planetary Protection Group, NASA Jet Propulsion Laboratory, Pasadena, California, United States of America; 4 Space Biosciences Research Branch, NASA Ames Research Center, Moffett Field, California, United States of America; 5 KBRwyle, NASA Ames Research Center, Moffett Field, California, United States of America; 6 Department of Pharmaceutical Chemistry, University of California San Francisco, San Francisco, California, United States of America; 7 Microbiology Lab, Wyle Laboratories, NASA Johnson Space Center, Houston, Texas, United States of America; Charles P. Darby Children’s Research Institute, UNITED STATES

## Abstract

The International Space Station (ISS) is a complex built environment physically isolated from Earth. Assessing the interplay between the microbial community of the ISS and its crew is important for preventing biomedical and structural complications for long term human spaceflight missions. In this study, we describe one crewmember’s microbial profile from body swabs of mouth, nose, ear, skin and saliva that were collected at eight different time points pre-, during and post-flight. Additionally, environmental surface samples from eight different habitable locations in the ISS were collected from two flights. Environmental samples from one flight were collected by the crewmember and samples from the next flight were collected after the crewmember departed. The microbial composition in both environment and crewmember samples was measured using shotgun metagenomic sequencing and processed using the Livermore Metagenomics Analysis Toolkit. Ordination of sample to sample distances showed that of the eight crew body sites analyzed, skin, nostril, and ear samples are more similar in microbial composition to the ISS surfaces than mouth and saliva samples; and that the microbial composition of the crewmember’s skin samples are more closely related to the ISS surface samples collected by the crewmember on the same flight than ISS surface samples collected by other crewmembers on different flights. In these collections, species alpha diversity in saliva samples appears to decrease during flight and rebound after returning to Earth. This is the first study to compare the ISS microbiome to a crewmember’s microbiome via shotgun metagenomic sequencing. We observed that the microbiome of the surfaces inside the ISS resemble those of the crew’s skin. These data support future crew and ISS microbial surveillance efforts and the design of preventive measures to maintain crew habitat onboard spacecraft destined for long term space travel.

## Introduction

An increased focus on the relationship between indoor buildings and human health has led to a recognition that the International Space Station (ISS) is particularly well suited for studying microbiome within structures because the orbiting outpost is a uniquely isolated “island” in space [1]. Onboard the ISS, investigators can systematically track microbial arrival, circulation, and transmission—unlike other settings used for studying microbiology of built environments, such as homes [2], hospitals [3], submarines [4], subways [5], aircraft [6], or remote scientific stations [7]. Moreover, the influx and outflow of microorganisms at the ISS generally occurs at preplanned intervals when crewmembers, cargo, and experiments are exchanged between transport spacecraft. For these reasons, the ISS is an unparalleled location for studying the interactive dynamics between humans and microorganisms inside closed habitats. Comparatively, terrestrial efforts to monitor microbial dynamics in buildings must address a variety of confounding study factors (e.g., ventilation, human occupants, environmental conditions such as temperature, humidity).

Remarkably, up to 40% of microbial signatures inside terrestrial buildings are derived from humans living or visiting the space [8], due to microbial debris associated with shedding, exhalation, expectoration, skin shedding, cuts in the skin, bladder and bowel waste [9]. Hospodsky et al. [10] estimated that every hour approximately 10 million bacteria or fungi can be released inside a built environment by a single human occupant. People with uncompromised respiratory tract and healthy immune systems can usually fight off potentially harmful microbes residing within built habitats. However, human spaceflight introduces new risks that must be considered because astronauts can become more susceptible to infections during long spaceflight missions [11]. The beneficial, benign or detrimental pathways associated with microbe-host interactions in spaceflight remain poorly understood [12, 13]. Notably, recent ISS surveys of habitable surface, air, and water samples observed novel species, some of which might have become more abundant due to space-unique stressors including microgravity and solar/cosmic radiation [14–16]. It was also recently shown that *Zinnia* plants that were grown on the VEGGIE system on the ISS, had succumbed to wilt and rot disease due to a fungal pathogen, *Fusarium oxysporum* [17]. The plant pathogen was also isolated from surface wipes from the Microbial Tracking -1 study [18]. Numerous spaceflight experiments testing *Bacillus subtilis*, *Bacillus pumilus*, *Escherichia coli*, and *Salmonella typhimurium* reported mutations and a subset of differentially expressed genes associated, or not associated with virulence [19–24]. Taken together, the previous body of ISS microbiology research highlights many unknowns that can only be addressed with more systematic, spatiotemporal measurements directly correlated to ISS occupants. Collecting and interpreting microbial profiling data onboard will be crucial for future mission plans (i.e., traveling to the Moon or Mars) where rapid crew return and Earth-based medical interventions seem unlikely.

Recently, ISS surface samples from several locations were acquired and microbial diversity profiles were documented utilizing shotgun metagenome [25] and amplicon (bacteria and fungi) sequencing [26]. These investigations revealed a previously unmonitored richness of microbial diversity inside the ISS [25, 27]. One study has, for the first time, examined correlations between resident astronauts and the ISS surface microbiomes using 16S rRNA sequencing, and found that surface microbiome shares similarities to the skin microbiome [28]. The study also presented evidence that the microbial communities of the gastrointestinal tract, skin, nose and tongue change during the space mission. Recently, one astronaut was monitored for a 1-year spaceflight study, and the gastrointestinal microbiomes pre-, during and post-flight were analyzed and compared to a control subject on earth [29]. There were no significant differences between the two subjects in the overall microbial diversity, though the richness of the astronaut’s microbiome was lower than his control subject. Within the astronaut samples, there did not appear to be any significant decreases in richness or diversity in inflight samples relative to pre-flight and post-flight samples.

Motivated by a predecessor Microbial Tracking-1 project described by Singh et al. [25] and Checinska-Sielaff et al. [26], and the need for more comprehensive and metagenomic sequencing analysis of crew and environmental microbiome (including bacteria, archaea, fungi and viruses), we conducted a study called Microbial Tracking-2 (MT-2) that aimed to measure the relationship between environmental microorganisms and astronauts in spaceflight which could be insightful for future mission planners assessing threats or benefits to astronaut health (i.e., commensal, mutualistic, and pathogenic relationships). To achieve these objectives, eight defined ISS surface locations were sampled two times over the course of six months from 2017 to 2018, DNA extracted, and microbial diversity were catalogued using shotgun metagenome sequencing. Alongside these environmental surface samples, skin, ear, mouth, nostril and saliva samples provided by a crewmember (pre-flight, inflight, and post-flight) were collected to identify trends in temporal and spatial dynamics. Analyses with data collected from additional crewmembers will be needed to rigorously test identified trends. The overarching hypothesis for MT-2 was that the arrival, 6-month stay, and departure of a crewmember to the ISS could be sensed via an increase or perturbation of microbial similarity between crewmember body sites and various habitat surfaces. A related objective for MT-2 was to determine if the astronaut microbiome would inherit microbiome from the ISS and if those taxa would be retained months after returning from the mission.

## Methods

### Crewmember sample collection

Crewmember sample collection was a study approved by the Johnson Space Center Institutional Review Board (IRB) under IRB protocol Pro1974. Informed Consent forms were obtained and were part of the IRB approval. The crewmember was in spaceflight for a total of 135 days. Samples were collected from the crewmember at two time points (i.e., “day codes”) pre-flight: 180 (±30) days and 90 days before flight (day codes L-180 and L-90 respectively); three time points during flight: early (1st-2nd months), middle (2nd-4th months), and late (10 days before return) (day codes FD60, FD97, and R-9 (+126) respectively); and three time points post-flight: one day after return (day code R+1) (+136), 30 days (day code R+30) (+165) and 180 (±30) days after return (day code R+180) (+315). Body swabs include the mouth, nasal cavity, forehead, armpits, navel, forearms (antecubital fossa), navel region, and the back of both ears. Swabs were collected first thing in the morning, with no hygiene 6 hours prior to the session. Multiple saliva samples were collected and associated with each day code: L-180 (-180, -178, -176, -174); L-90 (-90, -88, -86, -84); FD60 (+60, +62, +64, +66); FD97 (+97, +99, +101, +103); R-9 (+126, +128, +130, +132); R+1 (+136, +138, +140, +142); R+30 (+165, +167, +169, +171); R+180 (+315, +317, +319). Due to the low biomass from forehead, armpits, navel and forearms, the DNA extracted from these four skin swabs were pooled for sequencing analysis. One saliva R+180 sample did not have a successful sequencing library preparation due to poor DNA quality. The total number of samples for downstream analysis were 64 (Table 1).

**Table 1:** Number of saliva and body swab samples from one crewmember. FD stands for Flight Day. L stands for Launch. R stands for Return. Crew member was aboard ISS during Flight 4.

	Pre-flight		During flight			Post-flight			Total
*Day Code*:	L-180 (-180)	L-90 (-90)	FD60 (+60)	FD97 (+97)	R-9 (+126)	R+1 (+136)	R+30 (+165)	R+180 (+315)	
**Saliva**	4	4	4	4	4	4	4	4	32
**Mouth**	1	1	1	1	1	1	1	1	8
**Ear**	1	1	1	1	1	1	1	1	8
**Nostril**	1	1	1	1	1	1	1	1	8
**Pooled skin (forehead, armpits, navel, forearm)**	1	1	1	1	1	1	1	1	8
**Total**									64

For saliva sample collection, a sample was collected on days 1, 3, 5 and 7 of each pre-, during, and post-flight time point (i.e., day code) to cover possible variations between different days. Saliva samples were collected in the morning just after waking up and before eating and drinking, and no exercise 4 hrs prior to collecting samples. SalivaBio Swabs (Salimetrics, LLC, Carlsbad, CA) were used for saliva collection as described [30]. For skin and mouth, a polyester swab, EnviroTrans Saline 0.85% Swabs (SRK35 from Hardy Diagnostics, Santa Maria, CA), was used. Prior to sample collection, the subject was required to wear a pair of sterile gloves, Kimtech Pure G3 Sterile Nitrile Gloves (Kimberly-Clark Professional, Roswell, GA). Each swab was pressed on the skin or in the mouth and the nose with different pressure (light, moderate, and strong) based on the sampling location and swabbed in serpentine or rotation patterns. A control swab, waving the swab in the air for 10 seconds, was also collected at each time point. The samples were frozen at or below -80°C onboard the ISS and during transit back to Earth and kept frozen until processed at the NASA Jet Propulsion Laboratory (JPL) upon arrival.

For DNA extraction, saliva rolls and body swabs were vortexed in 4 mL of Phosphate Buffered Saline (PBS) at maximum speed for 2 min. Two mL of liquid was collected, one mL for bead beating to lyse the microorganisms that are hard to lyse without bead beating; and the other mL was not subjected to bead beating in order to preserve the microorganism that might be sheared by this procedure. The two fractions with and without bead beating (2 mL) were then placed in a Maxwell cartridge for DNA extraction as described previously [25]. The DNA was eluted in 60 μL sterile water. The procedure was repeated starting with the remaining 2 mL of liquid after vortexing so two tubes of 60 μL total DNA were collected. DNA extraction was performed with the Maxwell 16 automated system (Promega, Madison, WI), in accordance with manufacture instructions using the Maxwell 16 Tissue LEV Total RNA purification kit.

### ISS surface sample collection

Environmental surface wipes were collected from the same locations and in the same manner as previously described [25]. Briefly, eight locations within the US on-orbit segments were sampled: Node 1, Node 2, and Node 3; US Laboratory Module; and Permanent Multipurpose Module (PMM). In this paper, location #1 is labeled as “port_panel”; #2 as “WHC”; #3 as “ARED_foot_platform”; #4 as “dining_table”; #5 as “overhead_4; #6 as “PMM_port_1”; #7 as “lab_overhead_3”; and #8 as “port_crew_quarters”. The crewmember collected Flight 4 environment surface samples. Flight 5 surface samples were collected after the crewmember had departed. The number of samples and locations from Flights 4–5 in MT-2 and Flights 1–3 from MT-1 is shown in Table 2.

**Table 2:** Number of surface samples per location per flight.

	Microbial Tracking 1			Microbial Tracking 2		Total
***Flight***:	1	2	3	4	5	
**port_panel**	1	1	1	1	1	5
**WHC**	1	1	1	1	1	5
**ARED_foot_platform**	1	1	1	1	1	5
**dining_table**	1	1	1	1	1	5
**overhead_4**	1	1	1	1	1	5
**PMM_port_1**	0	0	0	1	1	2
**lab_overhead_3**	1	1	1	1	1	5
**port_crew_quarters**	1	1	1	1	1	5
						37

Sample collections were conducted using pre-moistened sterile polyester wipes (23 cm × 23 cm; ITW Texwipe, Mahwah, NJ) that were placed in a sterile Ziploc bag and sent to the ISS as described [25] except that samples were collected during early flight for Flight 4 (64 days after launch), and mid-flight on Flight 5 (approximately 120 days after the crewmember’s departure from the station). Collected samples were stored at 4°C prior to return. This is in contrast to samples from Flights 1–3, which were stored at room temperature due to power restrictions [25]. Upon return, samples were processed and concentrated using the InnovaPrep concentrating pipettor [31]. DNA extraction was performed using the Maxwell 16 System (Promega, Madison, WI) in accordance with the instructions provided by the manufacturer.

### Metagenomic sequencing

The Illumina NextSeq500 was used for shotgun metagenomic sequencing with the NextSeq 500/550 300 cycle High Output Sequencing Kit v2.5 (Catalog number 20024908, Illumina, San Diego, CA), using 150 base pair, paired-end reads. DNA libraries were prepared for sequencing using the Nextera Flex DNA Library Preparation Kit (20018705, Illumina). Quality and fragment size were assessed on the Agilent Tapestation 4200 (Agilent, Santa Clara, CA). Libraries were quantitated using the Qubit fluorimeter (Thermo Fisher Scientific, Waltham, MA) and normalized to equivalent DNA quantities where possible, pooled, and diluted according to the manufacturer’s standard recommendations. The numbers of Illumina read pairs from 16 environmental surface (8 locations x 2 flights (F4 and F5)), 63 crewmember (one sample failed to produce usable data) samples, and control samples are shown in S1 Table.

The metagenomic sequence data generated from the ISS surfaces in this study can be found under NCBI Sequence Read Archive (SRA) under the bio-project number PRJNA497280. The crewmember associated microbiome sequencing data is deposited in NASA Life Sciences Data Archive (LSDA) (https://lsda.jsc.nasa.gov/Dataset). All intermediate analysis files are provided in Supporting document as Zip File Archive (S1–S10 Datasets). The sample metadata is provided in S9 Dataset.

### Taxonomic classification

#### Livermore metagenomics analysis toolkit analysis

The metagenomic sequencing data from crewmember samples and ISS surface samples from Flights 4 and 5 were analyzed using the Livermore Metagenomics Analysis Toolkit. LMAT is a metagenomic analysis pipeline to search for taxonomic identifiers associated with *k*-mers found in its reference genome database [32, 33]. LMAT operates and is parameterized differently from the pipeline in [25] resulting in potentially higher sensitivity. In recent metagenomic sequencing benchmarking studies, LMAT was shown to have good limit of detection (~80% sensitivity for genomes with 0.04X coverage), and precision in the range of 20–100%, which can be tuned by post-hoc thresholding on abundance depending on whether remote homology detection is desired (e.g., mapping shuffled reads to nearest phylum) [34, 35]. Reads mapping to the genus *Homo* were removed prior to depositing to public databases. Counts of reads mapping to kingdom Metazoa, Viridiplantae, or which were not mapped at the kingdom level were removed, as well as reads mapping to synthetic constructs at the species level. Metagenomic sequences from Flights 1–3, generated from MT-1 [25], were also re-analyzed using LMAT for consistency in analysis. Per sample relative abundance of each genus or species in the LMAT database was estimated from the proportion of reads assigned at the genus and species in each sample. For brevity, we refer to this quantity as the proportion of the taxon throughout the remainder of the paper. Though, this is a biased estimate because genome lengths and genome copy numbers vary across species and even within a strain. In addition to cellular abundance, this variation affects the proportion of reads sequenced from each taxon [36]. All LMAT read analysis files (all ranks, genus level, species level) are included in supporting document as Zip File Archive (S5–S7 Datasets).

#### Alpha diversity estimation

The alpha diversity of each sample was estimated separately with the phyloseq (version 1.24.2) [37] and the vegan (version 2.5–4) [38] packages, using the functions: **estimate_richness**, **estimateR**, **specnumber**, and **renyi**. We quantified alpha diversity at both the genus and species level using Hill numbers of order *a* (N_*a*_) [39], also known as the effective number of observed taxa. The effective number of taxa is weighted by each taxon’s relative abundance of reads per sample [39]. Hill numbers of order *a* = 0, 1, and 2 correspond to the following popular diversity indices: observed richness (i.e., N_0_ is richness, a.k.a, the number of taxa seen), exponentiated Shannon index (N_1_ is **exp(H)**), and the reciprocal Simpson index (N_2_ is **1/S**). Detailed methods are available in S1 Text Section 4.2.2.

#### Ecological distances

Samples were visualized by non-metric multidimensional scaling and Principal Coordinates Analysis (NMDS and PCoA) with Jaccard and Euclidean distances respectively, using the function **ordinate** from the phyloseq package (version 1.24.2) [37]. Jaccard and Euclidean distances were calculated between samples using phyloseq’s **distance** function. The read counts for each taxon (treated as compositions) were transformed to Euclidean space via the centered log-ratio (clr) transform [40, 41]. Therefore, transformed values should be interpreted as relative to the mean abundance in each sample. Crewmember samples, Flights 4–5 surface samples from this study and Flights 1–3 surface samples from the MT-1 study were included in the analysis.

Sample distances were visualized within and between groups of interest: skin and flight surfaces from flights F1-5, body sites and Flight 4. Assessment of the differences among the sets of between-group distances was done via Kruskal-Wallis rank sum test.

We additionally performed a permutational multivariate analysis of variance (PERMANOVA) [42, 43] to test whether these distances were significantly different within groups of samples versus among groups. We used **adonis2** from the vegan package (version 2.5–4) [38] for the PERMANOVA, including terms for sequencing effort, surfaces vs crew, flight group, flight status, whether the sample is oral (mouth or saliva), whether is sample is from skin or Flight 4 or Flight 5 (See Section 5.3.2 PERMANOVA in S1 Text for detailed procedures).

#### Differential abundance

The package ALDEx2 (version 1.12.0) [40] was used to transform relative abundances prior to ordination (**aldex.clr**), and for testing for differentially abundant taxa (**aldex.glm**). For each taxon and instance, the method performs both a Kruskall-Wallis test between conditions and a likelihood ratio test for including a condition term as an explanatory variable in a generalized linear model (“glm” test). *P*-values are adjusted for the false discovery rate (FDR) using the method of Benjamini and Hochberg (reported as “kw.eBH” and “glm.eBH” for the Kruskall-Wallis and glm tests, respectively). Finally, the multiple-taxa-adjusted *P*-values for each taxon are averaged across the sampled Dirichlet instances.

#### Microbial Source Tracking

SourceTracker models the composition of each “sink” sample as a mixture of compositions from presumed “source” environments using Latent Dirichlet Allocation, producing an expected value for each sink’s contribution from each source over a series of Gibbs sampling runs [44]. ISS surface samples were designated as sinks and the crewmember sequences were designated as a source environment of sequencing reads that unambiguously map to species. SourceTracker adds an additional “Unknown” environment as a source with uniform read counts to account for sources absent in the experiment. SourceTracker performed Gibbs sampling 10 times with a burn-in of 100 passes before drawing a sample with the following default parameters: **alpha1 = 0.001**, **alpha2 = 0.001**, **beta = 10**. Alpha1, alpha2, and beta^-1^ represent prior knowledge of uniform counts in the known and unknown sources and in the sinks as a proportion of the number of reads in the sinks. Gibbs samples were of size 1000 reads.

## Results

### Crewmember body site microbiome

#### Prevalent and abundant microbial genera and species

**Fig 1:**
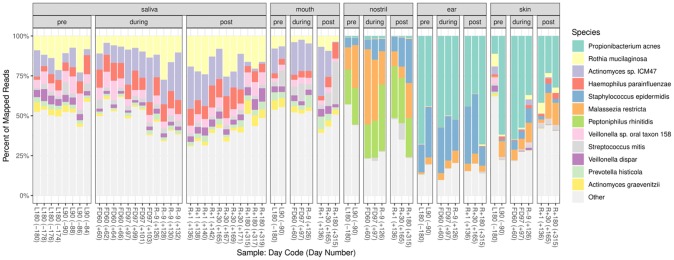
Top 12 most abundant species from crewmember samples pre-, during- and post-flight. The percent of mapped reads from saliva, mouth, nostril, ear and skin samples for each species in each sample (ranked by the average abundance in each panel summed across locations). Each sample’s time point label (day code) and day number are shown.

Overall, sequencing reads from crewmember samples were mapped to 1,394 genera and 5,192 species. The top 12 microbial species detected from all crewmember samples pre-, during and post-flights are shown in Fig 1. Ear and skin samples were dominated by *Propionibacterium acnes*, a common skin associated bacteria [45]. *Staphylococcus epidermidis* is prevalent in both Ear and nostril samples. *Malassezia restricta* and *Peptoniphilus rhinitidis* are prevalent in nostril samples. Mouth and saliva samples were dominated *Rothia mucilaginosa*, *Actinomyces* sp. *ICM47*, *Haemophilus parainfluenza*, and *Veillonella* sp. *oral taxon 158*. These organisms are associated with oral microbiome [46]. The top five most abundant genera in each of the body site are shown in S2 Table. *Propionibacterium* was the most abundant bacterial genus in skin and ear. *Streptococcus*, *Prevotella*, and *Actinomyces* are the top three genera in mouth and saliva. In nostril, *Corynebacterium*, *Propionibacterium* and *Staphylococcus* are the most abundant. A detailed list of the prevalent species and genera by sample type is included as Zip File Archive in supporting document (S5–S7 Datasets).

#### Microbial diversity in crewmember saliva

**Fig 2:**
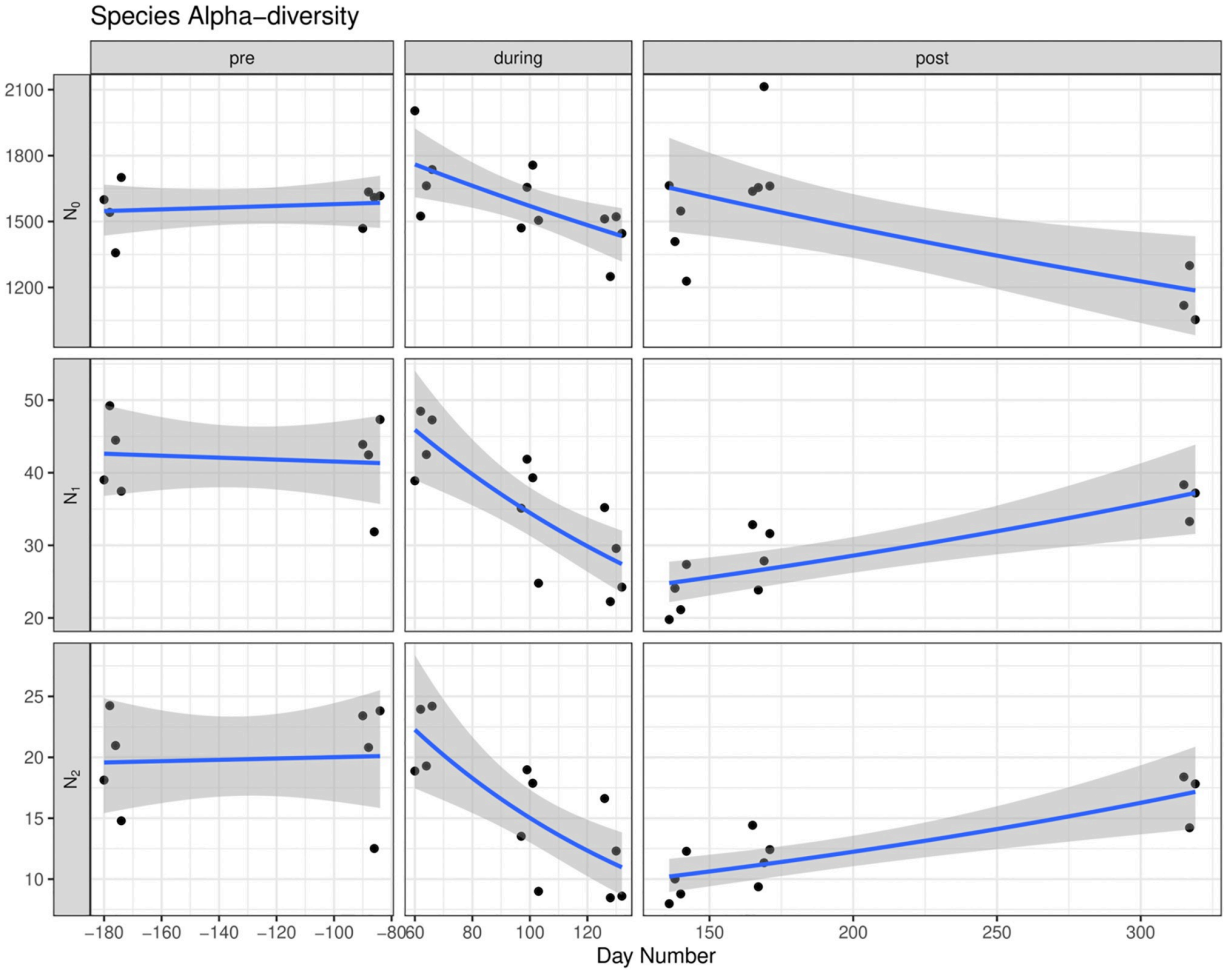
Alpha diversity for crewmember saliva samples at the species level resolution over time. Alpha diversity is quantified as Hill numbers corresponding to transformed richness (N_0_), Shannon (N_1_), and Simpson (N_2_) diversity indices. Samples are shown as black points (round). A gamma regression line (blue) is fitted to the points with a 95% confidence interval in grey.

*Alpha diversity*. In saliva samples, a total of 748 genera detected were shared among pre-, during and post-flight samples, additionally, 59 taxa were unique to pre-flight, 93 to during flight and 80 to post-flight (S1 Fig). Alpha diversity was quantified as the effective number of species or Hill numbers, N_0_ (Richness), N_1_ (exp{Shannon’s diversity}), and N_2_ (reciprocal of Simpson’s diversity). Fig 2 shows the three diversity measurements per saliva sample pre-, during and post-flight. All three diversity indexes showed that the average species diversity at the two time points pre-flight (days -180, -90) were similar. The species diversity decreased across the three time points during flight (days 60, 97, 126). N_1_ and N_2_ showed that the saliva microbial diversity gradually recovered across the three time points post-flight (days 136, 165, 315), though still not to the same level as pre-flight. The species richness (N_0_) recovered at day 136 and day 165 post-flight, but there was a drop at day 315, indicating that the species richness was not fully recovered after 6 months of return.

**Fig 3:**
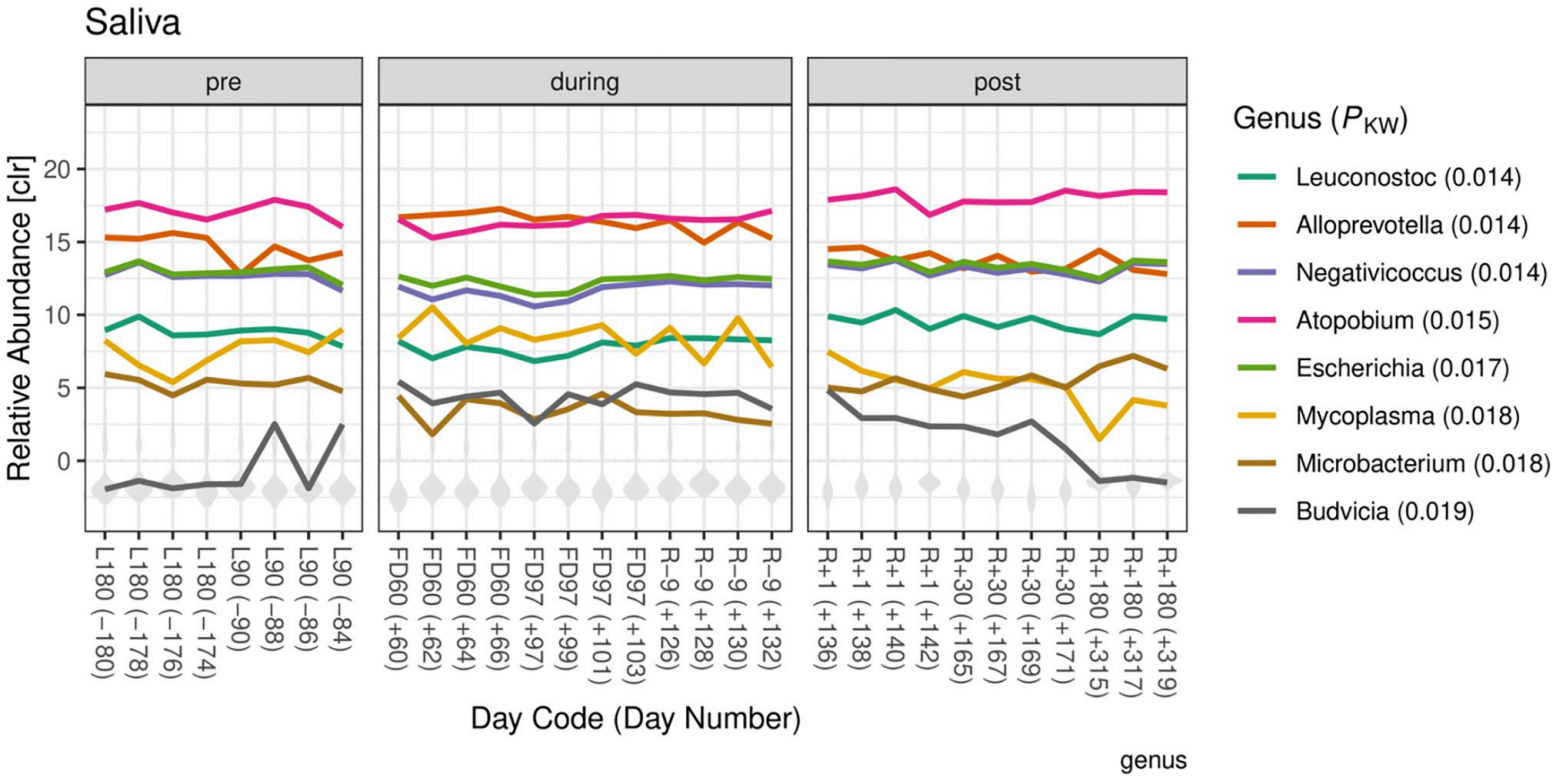
Relative abundances of genera that were differentially abundant among flight state in saliva samples. The relative abundances of the eight genera with the smallest Kruskall-Wallis *P*-values are shown in color. The distribution of relative abundances from other genera are shown in grey. Abundances are log transformed and relative to the geometric mean abundance per sample using genera that were present within each flight state (centered log-ratio transform).

*Differential abundance by flight state*. The observations in alpha diversity changes in saliva supported further investigation of differential abundance amongst flight states. Fig 3 shows the relative abundance of the eight genera with smallest adjusted *P*-values for a differential abundance test among pre-, during and post-flight samples (Kruskall-Wallis *P* < 0.02). The *P*-values associated with the eight genera area also shown in Fig 3. The relative abundance of *Microbacterium*, *Leuconostoc*, *Negativicoccus*, *Escherichia*, and *Atopobium* during flight was decreased. The abundance of *Budvicia* and *Alloprevotella* was elevated during flight. The abundance of *Alloprevotella*, *Escherichia*, *Negativicoccus*, *and Microbacterium* in post-flight samples returned to the level closer to pre-flight. The abundance of *Atopobium and Leuconostoc* post-flight returned to the level more similar to pre-flight. Of these eight genera, abundance of *Budvicia* has the most variation within pre- and post-flight states, with relative abundances both above and below the sample averages. The full set of differential analysis at the genus level is provided in Zip File Archive (S8 Dataset).

### ISS surfaces microbiome

**Fig 4:**
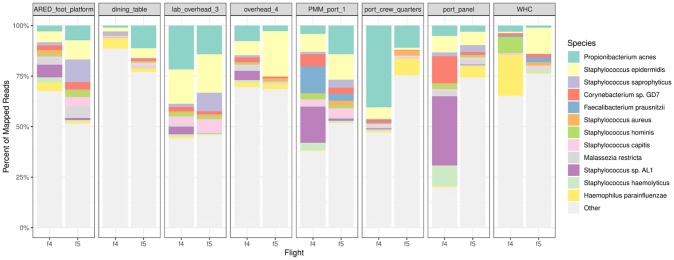
Relative abundances of top 12 species in environmental samples in Flights 4 and 5. The proportion of mapped microbial reads assigned to each genus is shown for each environmental sample. The top 12 genera are shown in colors and light grey (ranked by the average abundance in each panel summed across locations). Other less abundant genera are lumped together in lighter grey.

Reads were mapped to 1,351 genera and 5,281 species across all environmental surface samples from Flights 4 and 5 (16 samples). Fig 4 shows the relative abundance of the top 12 species across eight locations. *Propionibacterium acnes* and *Staphylococcus epidermidis* are the most prevalent in all eight surface locations from both Flights 4 and 5. *Staphylococcus* sp. *AL1* and *Corynbacterium* sp *GD7* are the most dominant in port_Panel and PMM_port_1 from Flight 4, but not in Flight 5. *Staphylococcus saprophyticus* is more abundant in Flight 4 than Flight 5 in ARED_foot_platform and lab_overhead_3 locations. A detailed list of the prevalent species and genera by sample type is included as zipped archive file in supporting document (S5–S7 Datasets).

### Similarity of microbial profiles from crewmember and ISS surface samples

**Fig 5:**
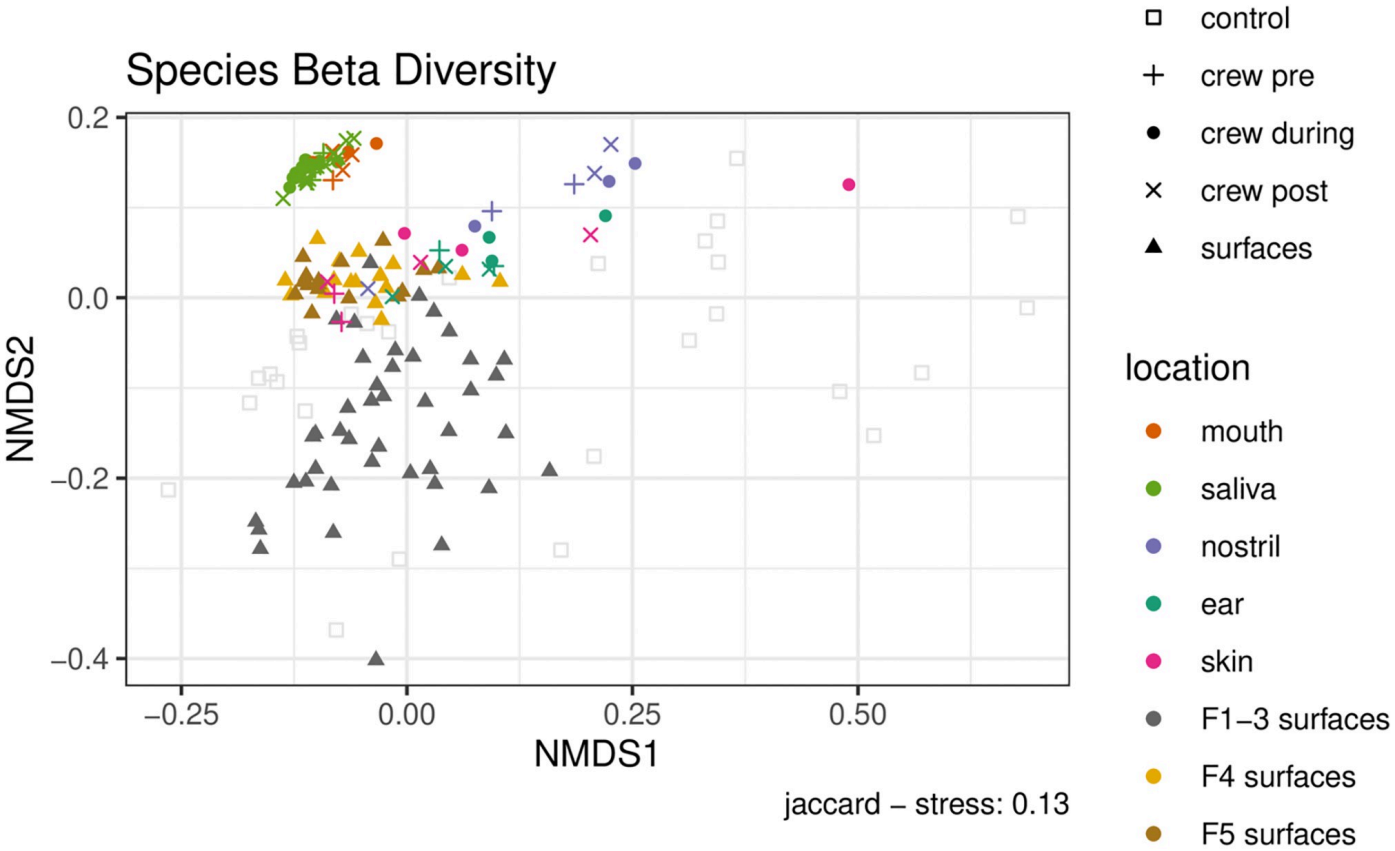
NMDS ordination of samples by Jaccard distance at species level resolution. Environmental samples are shown as gray circles (Flights 1–3 [2]) and brown and yellow circles (Flights 4, 5 this analysis). Samples from the crewmember (this analysis) are shown as circles for during flight, plus signs (+) for pre-flight, and crosses (x) for post-flight. Skin samples are colored pink; mouth as red, saliva lighter green, nostril purple, ear darker green. Control samples are semitransparent squares.

Beta-diversity estimates the differences between two microbiomes or samples by quantifying the overlap of shared taxa between them. The similarities and differences between crewmember and surface samples were visualized using NMDS with Jaccard distances (Fig 5). Flights 4 and 5 surface samples are more similar to crewmember samples than Flights 1–3 from previous Microbial Tracking -1 study. Within crewmember samples, skin samples appear to overlap with ISS surface samples, and they are closer to surface samples than other crewmember samples including saliva, mouth, nose and ear. When the during flight skin sample group was compared to each of the Flights 1–5 surface sample groups, the distances were different among flights (Kruskal-Wallis P < 0.001). The median distance between skin microbiome to Flights 4 and 5 surface microbiomes were smaller than to Flights 1–3 (S2 Fig). When Flight 4 and skin samples were compared, a large percentage of species in skin were shared with F4 surfaces, with the during flight samples sharing the most species (pre: 93.8%, during: 97.0%, post: 94.6%) (S3 Table).

The distances between all of Flight 4 surface samples and each of the during flight crewmember body sample groups (ear, mouth, nostril, saliva and skin) were also compared, and the medians differ among body sites (Kruskal-Wallis P < 0.001) (S3 Fig). Additionally, PERMANOVA analysis showed that body sites rather than flight state (pre, during and post) were more important in explaining differences among samples (P = 0.001 vs P = 0.152) (S1 Text Section 5.3.4 and 5.3.5). For ISS surface samples, PERMANOVA analysis showed that Flight numbers rather than surface locations were more important in explaining differences among samples (P = 0.001 vs P = 0.267).

### Microbial contribution from crewmember to surfaces

**Fig 6:**
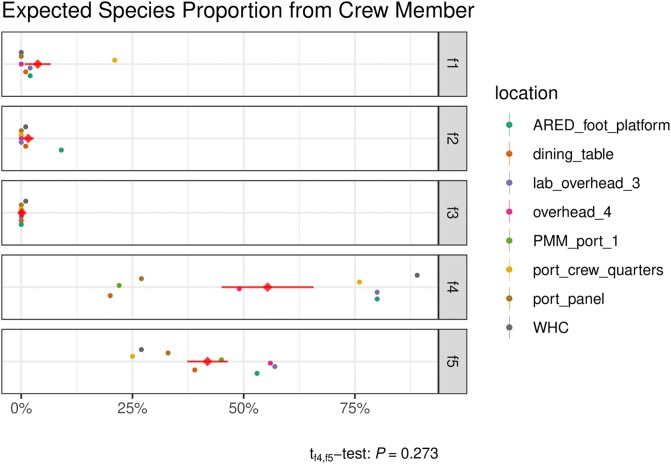
Assessment of astronaut contribution to the ISS microbiome. SourceTracker was used to assess the proportion of crewmember 1 microbiome sequences to Flight 4 and Flight 5 ISS surface microbiome sequences. This was done by comparing the species compositions of reads found in the crewmember inflight samples (“source”) with those of various flight samples (“sink”). The expected proportion of reads contributed by crewmember 1 for each sink (i.e., surface location, by flight) is shown along the x-axis. The standard deviation of the predicted proportions for each sink was either 0.00 or 0.01, computed from 10 Gibbs samples per sink location, and thus treated as point estimates. The mean contribution per flight across surface locations (diamond) and its standard error are shown in red.

SourceTracker was used to assess the proportion of sequences in the F4 and F5 microbiomes that could be expected to come from the crewmember microbiome (Fig 6). F1-3 data was also included for comparison. The proportion of crewmember sequences contributing to the F4 microbiome was on average 56%, ranging from 20% at Dining Table to 89% at WHC. Flight 4 environmental surface wipes were collected two months after the crewmember arrived on the ISS. The crewmember’s microbiome persisted even after their departure from the ISS and was still evident during the F5 sampling event, which occurred four months after the crewmember had departed. The proportion of the crewmember’s sequences during F5 sampling was on average 42% and ranged from 25% to 57%. The average contribution from the crewmember to F4 versus to F5 were not statistically different (P = 0.27 paired t-test). As expected, there was negligible predicted contribution from the crewmember to F1, F2 and F3 as these were sampling dates prior to the crewmember’s arrival.

### Comparison of surface microbiome from Flights 4–5 to Flights 1–3

In Flights 4 and 5, 208 microbial genera were seen in each sample (100% prevalence in 16 samples). In Flights 1–3 which were conducted under MT-1, 42 genera were seen in each sample (100% prevalence in 21 samples). The top 12 most abundant genera from both studies are shown in S4 Fig. The top three genera, *Propionibacterium*, *Staphylococcus*, *Streptococcus* from Flights 4 and 5 are also among the top 15 in MT-1 (Flights 1–3) (S4 Table). The overall number of detectable genera that are shared between samples from MT-1 and MT-2 were 990 (70% of all MT-1 or MT-2 genera), 361 genera were unique to MT-2 (26%), while 54 were unique to MT-1 (4%) (S5 Fig). The detailed list of genera detected from MT-1 vs MT-2 and the prevalence of the genera in each study is included in S10 Dataset. Additionally, the genera that are specific to MT-1 is included in S5 Table and genera that are specific to MT-2 is included in S6 Table.

## Discussion

Learning the rules governing microbial communities is important for the advancement of spaceflight activities. In a relatively small mostly closed environment, the consequences of these communities deviating from normal could present significant challenges—as minor as increased cleaning time to food, air, and water problems that can have negative implications for crewmember health and the success of the whole mission. Studies have shown that the environmental microbiome can affect both the human microbiome and human health outcomes such as metabolic and immune function [47]. Human occupancy is also a source of indoor bacteria such as *Propionibacterineae*, *Staphylococcus*, *Streptococcus*, *Enterobacteriaceae*, and *Corynebacterineae* [48]. Better understanding of these microbiome interactions between humans and the shared environment will require continued monitoring, sampling, and development of effective detection and statistical analysis methods. A first step in understanding what normal microbial dynamics look like in human spaceflight is collecting data from ongoing successful missions.

Voorhies et al. have taken one of the first steps to show that the astronaut microbiome is affected by long term spaceflight and confirm that astronaut skin microbial composition is similar to ISS environmental surfaces [28]. The NASA twin study also monitored microbiome changes pre, during and post-flight, though no significant changes were observed [29]. The present data is the initial report of a study employing metagenomic sequencing to analyze the composition of crewmember microbiome at the ISS and its association ISS environmental microbiome, leveraging an extensive taxonomically annotated sequence database and a fast and sensitive *k*-mer based read mapping strategy. This is the first study to report an in-depth analysis of crewmember saliva microbiome changes due to spaceflight conditions.

The current study is limited to one crewmember subject. Analysis of samples from additional crewmember subjects will increase statistical significance and confidence of the observations. The current study has the most saliva samples since four saliva samples were collected every other day over a week for each of the eight time points (two pre-flight, three inflight and three post-flight). The saliva samples allowed better exploration of alpha diversity over time. In this study, environmental samples cross different flights grouped more strongly by flight than by location. The limited flights in this study could influence these observations and findings.

In this study, 63 samples from one crewmember and 16 environmental surface wipe samples collected from two separate flight missions were analyzed by metagenomic sequencing. Similar to other human microbiome studies, we have observed *Propionibacterium* as the most abundant bacterial genus in skin and ear. *Propionibacterium* was also the most abundant bacteria found on the surfaces of the ISS, present in all 16 surface samples analyzed from Flights 4 and 5 (Fig 4). When compared with ISS surface samples collected by the crewmember (Flight 4, collected in June 2017), another flight in the same MT-2 study (Flight 5, collected in January 2018) and three previous flights conducted in the prior MT-1 study (Flights 1–3, collected between March 2015 to May 2016), it was observed that the crewmember skin samples were more closely related to Flights 4 and 5 environmental samples than Flights 1–3, and skin samples were more similar to the ISS surface samples than saliva, mouth and nostril samples (Fig 5). The SourceTracker results (Fig 6) predicted that the crewmember microbiome contributed to 55% of the Flight 4 surface microbiome and 42% of the Flight 5 microbiome, averaged across all eight locations, with the largest contribution being at the WHC, the ARED foot platform, and the port crew quarters.

With the SourceTracker and ordination results together, we are inclined to believe that there could be an exchange of microbial composition between crewmember skin samples and surfaces at the ISS. This observation of microbiome exchange between crewmember microbiome and surface microbiome was also supported by another recent NASA research effort [28].

The oral microbiome is the second most diverse microbial community in the human body with distinct health and diseased state [49]. The effect of spaceflight on oral microbiome provides a good view of human microbiome. Environmental disruption can alter the microbial balance and lead to the overgrowth of pathogens. This could lead to tooth decay, gingivitis, and periodontal disease which can cause major discomfort and, in some cases, require medical treatment. In rare cases, poor oral hygiene can also result in more serious and life-threatening conditions such as endocarditis and heart-disease [49, 50].

The alpha diversity measurement in the crewmember’s oral microbiome is comparable to a previous study on core oral microbiomes from several healthy volunteers [51] and Human Microbiome Project [52, 53]. Saliva samples showed the most interesting trend of decreasing species diversity in the three time points during flight and increasing effective number of species (N_1_, N_2_) in post-flight time points, though not fully to pre-flight levels (Fig 2). The overall species richness (N_0_) decreased post-flight, which may be limiting N_1_ and N_2_. Saliva samples have been used by NASA researchers to study astronaut immune changes and health conditions. Herpesviruses were detected from saliva samples from crewmembers during spaceflight [30, 54], suggesting that saliva samples have the potential to serve as biomarkers to monitor crew health. The observation of a decreased in-flight alpha diversity in saliva microbiome could be due to the space conditions (e.g., microgravity, radiation) that can cause certain microbes to colonize and reduce overall diversity. This could also be driven by immune response changes associated with space travel. Several of the differentially abundant bacterial genera detected from saliva such as *Mycoplasma* [55] and *Microbacterium* [56] are considered opportunistic pathogens and could infect immunocompromised patients. *Alloprevotella* has been found to be associated with dental caries [57].

The microbial profiles from environmental surface samples from the current study showed both similarities and differences with the previous Microbial Tracking -1 study. The top three genera, *Propionibacterium*, *Staphylococcus*, *Streptococcus* from Flights 4 and 5 in this study are also among the top 15 in MT-1 (Flights 1–3) (S4 Table). The overall number of detectable genera that are shared between samples from MT-1 and MT-2 are 1,059. This data supports the notion that the microbial profiles in the ISS exhibit both spatial and temporal changes. The most abundant microbes seem to persist over time, but the overall composition and distribution of microbiome evolves over time. However, there is a difference on how the samples were stored during transport from ISS to Earth. The surface wipes from Flights 4 and 5 were stored at 4°C while the wipes from Flights 1–3 were stored at room temperature due to the lack of stowage facility during transport from ISS to Earth. This difference in storage condition could have contributed to some microbial variances.

Shotgun sequencing of metagenomic samples is valuable for microbial community data collection because it interrogates all genetic components in a complex sample. This study is limited to taxonomic analysis from metagenomic sequencing data from one crewmember body swabs and two surface sampling experiments. Additional analysis of functional components of the microbiome such as metabolic genes, virulence genes, antibiotic resistance genes will provide further understanding of the relationships between crewmember and space microbial profiles and potential impacts to crew health.

The international Space Station is a specialized built environment. There are constant and dynamic microbial exchanges between environment, microbes and humans in an enclosed environment. The space conditions including microgravity and radiation impact the human microbiome composition and potentially cause a dysbiosis of the health microbiome. Analysis of samples from additional crewmembers will further our understanding of the fluctuation of the microbiome pre-, during and post-flights from this crewmember. Taxa that are differentially abundant between conditions or environments, and over time, can be potentially developed as taxonomic markers for targeted biosurveillance that may be more feasible for inflight assays and be less expensive than shotgun sequencing. Investigation in this general direction holds potential for developing technology to forecast and respond to health impacts.

## Supporting information

S1 Fig Number of observed genera in saliva shared across flight state. The numbers of observed taxa in saliva amongst flight states vs the number of observed taxa in any of the flight states is proportional to the areas of the overlaps. A taxon is considered observed in a sample if LMAT mapped at least 1 read to it. A taxon is observed in a flight state if it is observed in any saliva sample in that flight state.
(TIF)

S2 Fig Between and within group comparisons of crew member during flight skin samples vs Flights 1–5 surface samples. Jaccard distances based on genus presence-absence are shown as points. The median is marked by a circle, with bars showing the middle 50% of the data.
(TIF)

S3 Fig Between and within group comparisons of crew member ear, mouth, nose, saliva and skin during flight samples vs Flights 1–5 surface samples. Jaccard distances based on genus presence-absence are shown as points. The median is marked by a circle, with bars showing the middle 50% of the data.
(TIF)

S4 Fig Relative abundances of the top 12 genera in environmental samples from Flights 4–5 and Flights 1–3. The proportion of mapped microbial reads assigned to each genus is shown for each environmental sample. The top 12 genera are shown in colors and light grey (ranked by the average abundance in each panel summed across locations). Other less abundant genera are lumped together in lighter grey.
(TIF)

S5 Fig Number of observed genera in ISS surface samples shared between studies. The numbers of observed taxa shared between MT-1 (Flights 1–3) and MT-2 (Flights 4, 5) vs the number of observed taxa in either study is proportional to the areas of the overlaps. A taxon is considered observed in a study if it is detected in any sample in that study.
(TIF)

S1 Table Read tally summaries of crew samples and ISS surface samples by location and type prior to LMAT processing. (XLSX)

S2 Table Top 5 most abundant species per body site per flight state by relative abundance. (XLSX)

S3 Table Crewmember skin samples and Flight 4 surface sample taxonomy comparison. (TXT)

S4 Table Top 20 most abundant genera in either Flights 1–3 or Flights 4, 5. Genus clr-transformed sample abundances are averaged and ranked within each study.
(PDF)

S5 Table List of genera that are detected only in MT-1 study. The prevalence of each genera is also included.
(XLSX)

S6 Table List of genera that are detected only in MT-2 study. The prevalence of each genera is also included.
(XLSX)

S1 Dataset LMAT read counts. (TSV)

S2 Dataset LMAT read counts for microorganisms. Read counts mapping to kingdoms Metazoa, Viridiplantae, or which were not mapped at the kingdom level were removed, as well as reads mapping to synthetic constructs at the species level.
(TSV)

S3 Dataset LMAT read counts for microbial genera. Read counts, clr-transformed read counts, and average read scores.
(TSV)

S4 Dataset LMAT read counts for microbial species. Read counts, clr-transformed read counts, and average read scores.
(TSV)

S5 Dataset Microbial genus prevalence and average abundance by group. Samples are grouped by location, PMA treatment, and flight status.
(TSV)

S6 Dataset Microbial species prevalence and average abundance by group. Samples are grouped by location, PMA treatment, and flight status.
(TSV)

S7 Dataset Microbial genus prevalence and average abundance by study (Flights 1–3 vs Flights 4, 5) for environmental samples. Samples are grouped by study and PMA-treatment.
(TSV)

S8 Dataset *P*-values for differential abundance tests amongst genera from all three flight states for saliva samples. Output of aldex.glm for genera sorted by Kruskal-Wallis and “glm” adjusted and unadjusted p-values. kw: Kruskal-Wallis test, glm: likelihood ratio test for including flight state in GLM of relative abundance; ep: expected P-value, eBH: adjusted expected *P*-value for controlling the false discovery rate.
(TSV)

S9 Dataset Types, locations, flight states for ISS surface samples from Flights 1–5 and crewmember samples. (TSV)

S10 Dataset Microbial taxonomy, ranking and prevalence from MT-1 and MT-2 studies. (TSV)

S1 Text A rendered RMarkdown book of the analyses. (PDF)
