# Supplementary figures and images for: Crewmember microbiome may influence microbial composition of ISS habitable surfaces
Source: PLoS One. 2020 Apr 29;15(4):e0231838. doi: 10.1371/journal.pone.0231838 (PMC7190111; doi:10.1371/journal.pone.0231838)

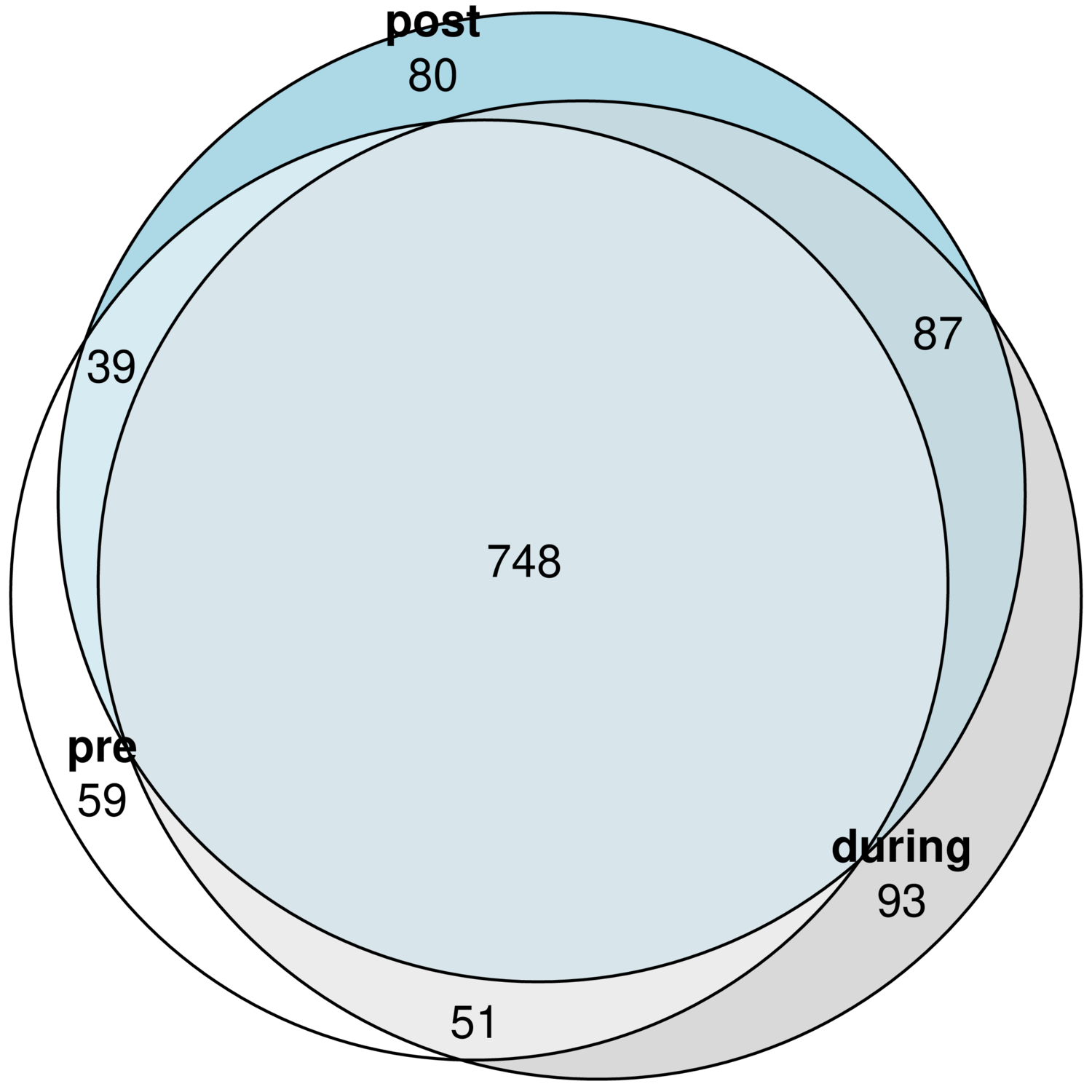

Supplement: S1 Fig — The numbers of observed taxa in saliva amongst flight states vs the number of observed taxa in any of the flight states is proportional to the areas of the overlaps. A taxon is considered observed in a sample if LMAT mapped at least 1 read to it. A taxon is observed in a flight state if it is observed in any saliva sample in that flight state. (TIF) [file pone.0231838.s001.tif]

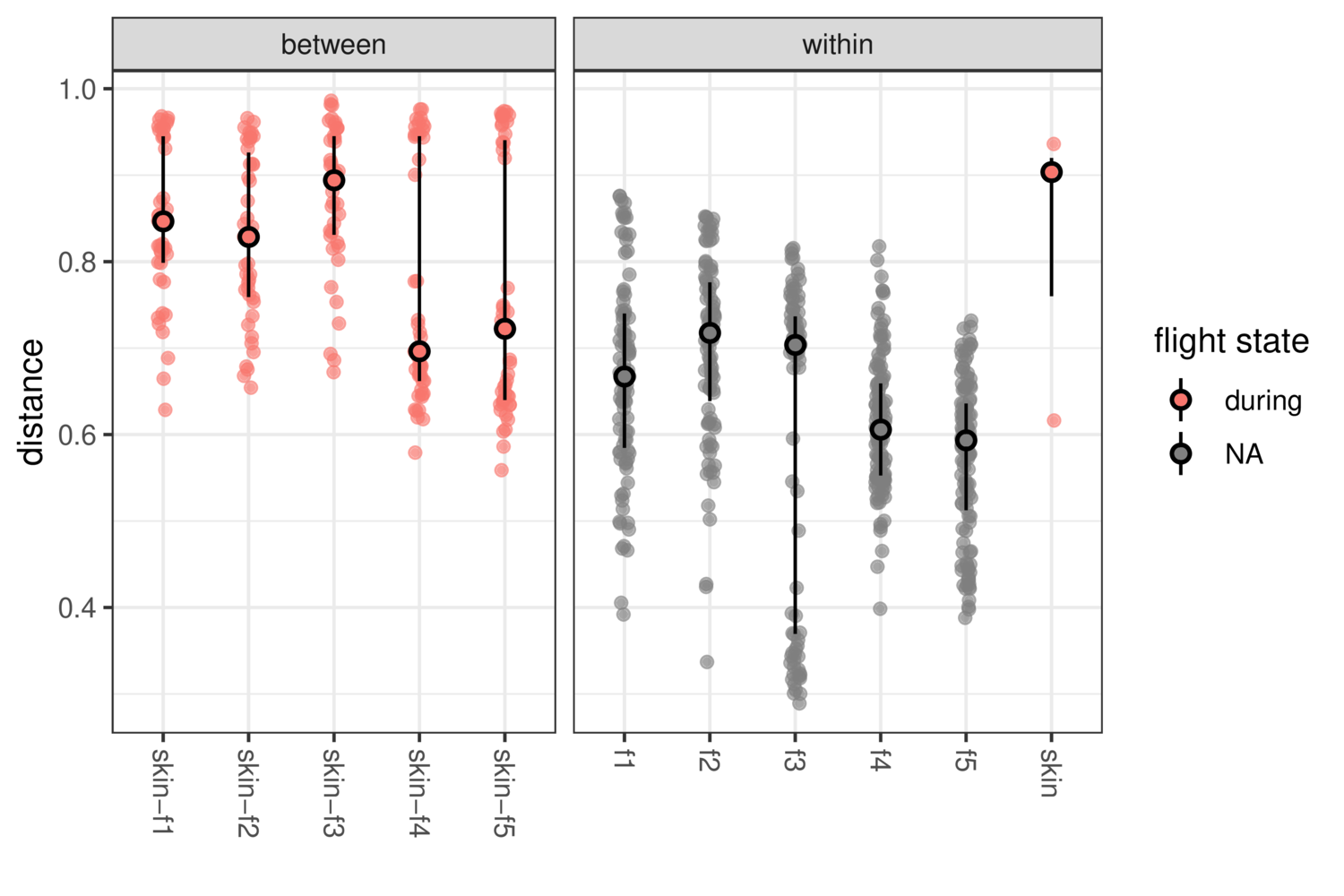

Supplement: S2 Fig — Jaccard distances based on genus presence-absence are shown as points. The median is marked by a circle, with bars showing the middle 50% of the data. (TIF) [file pone.0231838.s002.tif]

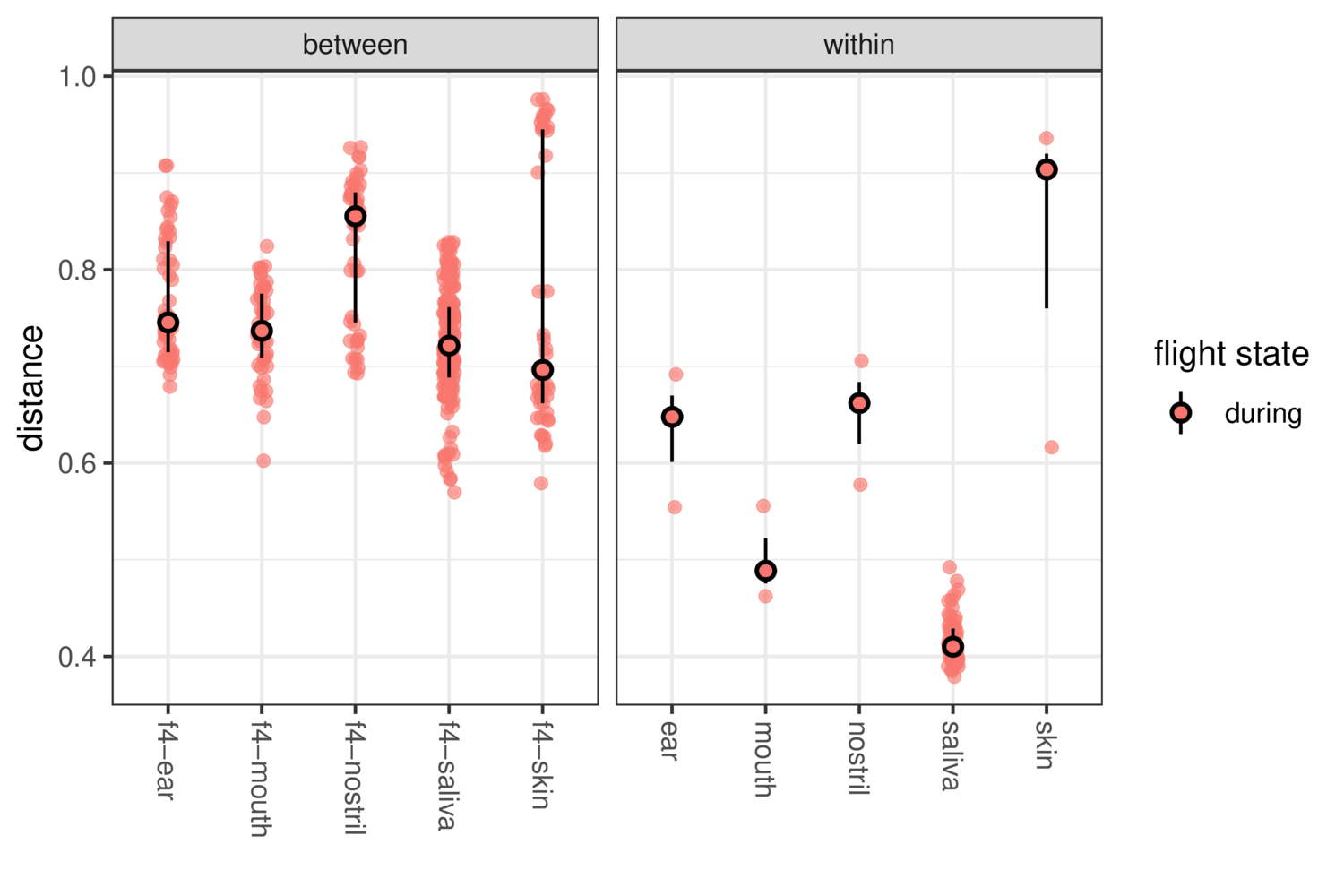

Supplement: S3 Fig — Jaccard distances based on genus presence-absence are shown as points. The median is marked by a circle, with bars showing the middle 50% of the data. (TIF) [file pone.0231838.s003.tif]

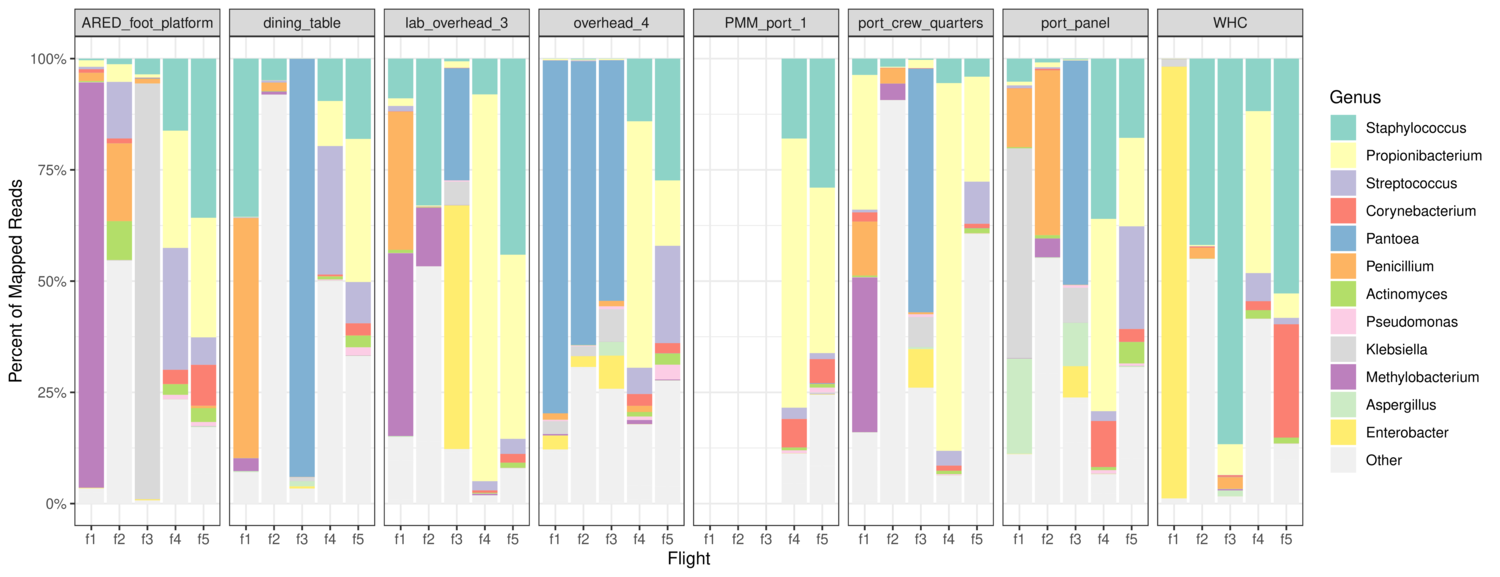

Supplement: S4 Fig — The proportion of mapped microbial reads assigned to each genus is shown for each environmental sample. The top 12 genera are shown in colors and light grey (ranked by the average abundance in each panel summed across locations). Other less abundant genera are lumped together in lighter grey. (TIF) [file pone.0231838.s004.tif]

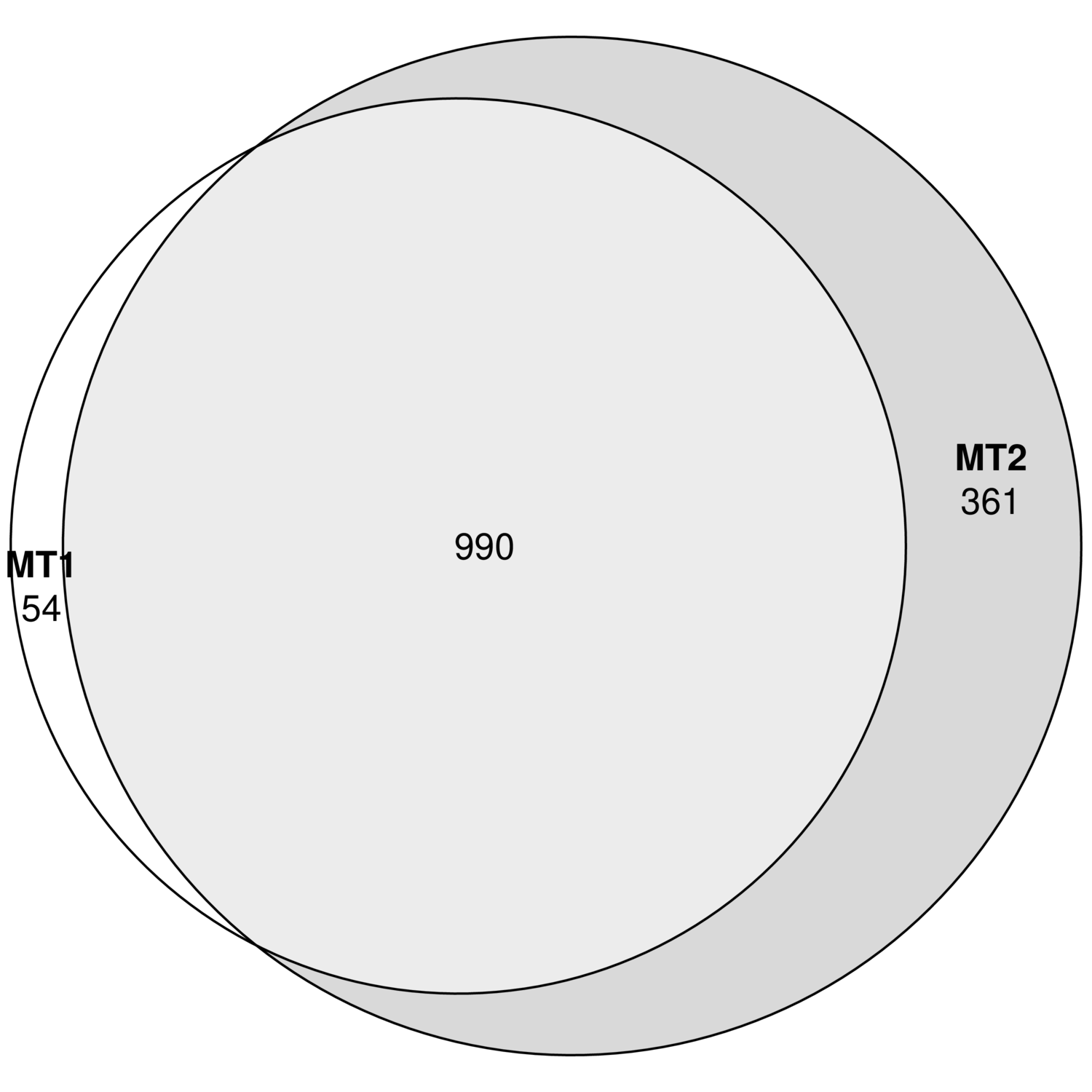

Supplement: S5 Fig — The numbers of observed taxa shared between MT-1 (Flights 1–3) and MT-2 (Flights 4, 5) vs the number of observed taxa in either study is proportional to the areas of the overlaps. A taxon is considered observed in a study if it is detected in any sample in that study. (TIF) [file pone.0231838.s005.tif]
