# Supplementary material for: Crewmember microbiome may influence microbial composition of ISS habitable surfaces
Source: PLoS One. 2020 Apr 29;15(4):e0231838. doi: 10.1371/journal.pone.0231838 (PMC7190111; doi:10.1371/journal.pone.0231838)
Supplement: S4 Table — Genus clr-transformed sample abundances are averaged and ranked within each study. (PDF) [file pone.0231838.s009.pdf]

genus	Flights 1–3			Flights 4, 5		
	rank	Cen(p) [%]	$\overline{\text{clr}(p)}$	rank	Cen(p) [%]	$\overline{\text{clr}(p)}$
Penicillium	1	33.28	16.30	60	0.03	6.71
Staphylococcus	2	22.84	15.76	2	16.09	15.84
Pantoea	3	2.83	12.74	116	0.01	4.39
Lecanosticta	4	2.62	12.63	423	0.00	0.03
Methylobacterium	5	5.87	13.03	12	0.39	10.46
Propionibacterium	6	1.72	12.02	1	63.66	17.82
Klebsiella	7	1.58	11.93	42	0.06	7.79
Aspergillus	8	1.60	11.92	54	0.04	7.17
Rhodotorula	9	1.45	11.78	314	0.00	1.17
Enterobacter	10	1.37	11.68	39	0.08	8.09
Puccinia	11	0.83	10.97	41	0.06	7.82
Pseudomonas	12	0.68	10.69	5	1.13	12.01
Streptococcus	15	0.32	9.57	3	3.56	13.66
Acinetobacter	18	0.23	9.11	8	0.67	11.25
Corynebacterium	21	0.14	8.36	4	2.61	13.21
Malassezia	30	0.05	6.86	7	0.73	11.37
Veillonella	40	0.02	5.80	9	0.56	10.99
Prevotella	44	0.02	5.50	10	0.53	10.92
Actinomyces	52	0.07	5.89	6	1.08	11.93
Rothia	92	0.03	4.31	11	0.47	10.74

PMA
