# Supplementary material for: Crewmember microbiome may influence microbial composition of ISS habitable surfaces
Source: PLoS One. 2020 Apr 29;15(4):e0231838. doi: 10.1371/journal.pone.0231838 (PMC7190111; doi:10.1371/journal.pone.0231838)
Supplement: S1 Text — (PDF) [file pone.0231838.s022.pdf]

Supplement to Crewmember microbiome may influence microbial composition of ISS habitable surfaces

Aram Avila-Herrera (avilaherrera1@llnl.gov)
Nicholas Be (be1@llnl.gov) James Thissen (thissen3@llnl.gov)
Camilla Urbaniak (camilla.urbaniak@jpl.nasa.gov)

2020-03-13

Contents

1 Prerequisites **5**
- 1.1 Data and Results 5
- 1.2 Code 5
- 1.3 Setup 5

2 Prepare Data **9**
- 2.1 Load LMAT read counts and sample data 9
- 2.2 Mapped reads 10
- 2.3 LMAT matchscores 14
- 2.4 CLR monte carlo 16
- 2.5 Select samples 17
- 2.6 Save 17

3 Prevalence and Abundance **19**
- 3.1 Geomtric mean 19
- 3.2 Groups of samples 20
- 3.3 Top taxa 20
- 3.4 Proportion bars 21
- 3.5 MT1 vs MT2 24
- 3.6 Caveats 26

4 Alpha diversity **27**
- 4.1 Richness and Evenness 27
- 4.2 N_1 vs. mapped microbial reads 27
- 4.3 Effective counts of taxa 29
- 4.4 Saliva α-diversity 31
- 4.5 Caveats 38

5 Beta-diversity **39**
- 5.1 Ecological distances 39
- 5.2 Ordination 39
- 5.3 PERMANOVA 46
- 5.4 aldex.glm 55
- 5.5 Saliva by flight state 55
- 5.6 "Venn" (Euler) Diagrams 58

6 SourceTracker **63**
6.1 F4 vs F5 64

```
knitr::opts_chunk$set(
  collapse = FALSE, comment = "#>",
  echo = params$show_code,
  cache = TRUE,
  cache.rebuild = params$rebuild
)
```

1 Prerequisites

This notebook is written in **Markdown**.

The **bookdown** package can be installed from CRAN or Github:

To compile this example to PDF, we'll need XeLaTeX. You are recommended to install TinyTeX (which includes XeLaTeX): https://yihui.org/tinytex/.

1.1 Data and Results

`data/` Minimally post-processed data are included in this directory. For example, here we will find read counts as processed by LMAT, a table of sample metadata, and pre-computed ALDEx2 objects.

`results/` Computed results such as summarized and filtered read counts, ranked lists of taxa, ordination coordinates, distances, etc…

`figures/` and `tables/` Main and supplementary figures and tables will be saved here.

1.2 Code

The bulk of the code exists in the Rmarkdown files (`*.Rmd`). Helper scripts are in `scripts/` and custom R functions will be in `R/`.

1.3 Setup

```
#> R version 3.6.2 (2019-12-12)
#> Platform:
#> Running under:
#>
#> Matrix products: default
#> BLAS/LAPACK: libopenblasp-r0.3.7.dylib
#>
#> locale:
#> [1] en_US.UTF-8/en_US.UTF-8/en_US.UTF-8/C/en_US.UTF-8/en_US.UTF-8
```

```
#>
#> attached base packages:
#> [1] stats     graphics  grDevices utils     datasets  methods   base
#>
#> other attached packages:
#>  [1] nvimcom_0.9-83   eulerr_6.0.0     phyloseq_1.28.0  kableExtra_1.1.0
#>  [5] ggridges_0.5.2   ggnomics_0.1.1   cowplot_1.0.0    vegan_2.5-6
#>  [9] lattice_0.20-38  permute_0.9-5    ALDEx2_1.16.0    glue_1.3.1
#> [13] magrittr_1.5     forcats_0.4.0    stringr_1.4.0    dplyr_0.8.4
#> [17] purrr_0.3.3      readr_1.3.1      tidyr_1.0.2      tibble_2.1.3
#> [21] ggplot2_3.2.1    tidyverse_1.3.0
#>
#> loaded via a namespace (and not attached):
#>  [1] nlme_3.1-144                bitops_1.0-6
#>  [3] matrixStats_0.55.0          fs_1.3.1
#>  [5] lubridate_1.7.4             webshot_0.5.2
#>  [7] httr_1.4.1                  GenomeInfoDb_1.20.0
#>  [9] tools_3.6.2                 backports_1.1.5
#> [11] R6_2.4.1                    DBI_1.1.0
#> [13] lazyeval_0.2.2              BiocGenerics_0.30.0
#> [15] mgcv_1.8-31                 colorspace_1.4-1
#> [17] ade4_1.7-13                 withr_2.1.2
#> [19] tidyselect_1.0.0            compiler_3.6.2
#> [21] cli_2.0.1                   rvest_0.3.5
#> [23] Biobase_2.44.0              xml2_1.2.2
#> [25] DelayedArray_0.10.0         bookdown_0.18
#> [27] scales_1.1.0                digest_0.6.23
#> [29] rmarkdown_2.1               XVector_0.24.0
#> [31] pkgconfig_2.0.3             htmltools_0.4.0
#> [33] dbplyr_1.4.2                rlang_0.4.4
#> [35] readxl_1.3.1                rstudioapi_0.11
#> [37] generics_0.0.2              jsonlite_1.6.1
#> [39] BiocParallel_1.18.1         RCurl_1.98-1.1
#> [41] GenomeInfoDbData_1.2.1      biomformat_1.12.0
#> [43] Matrix_1.2-18               Rhdf5lib_1.6.3
#> [45] Rcpp_1.0.3                  munsell_0.5.0
#> [47] S4Vectors_0.22.1            fansi_0.4.1
#> [49] ape_5.3                     lifecycle_0.1.0
#> [51] stringi_1.4.5               yaml_2.2.1
#> [53] MASS_7.3-51.5               SummarizedExperiment_1.14.1
#> [55] zlibbioc_1.30.0             rhdf5_2.28.1
#> [57] plyr_1.8.5                  grid_3.6.2
#> [59] parallel_3.6.2              crayon_1.3.4
#> [61] Biostrings_2.52.0           haven_2.2.0
```

```
#> [63] splines_3.6.2           multtest_2.40.0
#> [65] hms_0.5.3               knitr_1.28
#> [67] pillar_1.4.3            igraph_1.2.4.2
#> [69] GenomicRanges_1.36.1    reshape2_1.4.3
#> [71] codetools_0.2-16        stats4_3.6.2
#> [73] reprex_0.3.0            evaluate_0.14
#> [75] data.table_1.12.8       modelr_0.1.5
#> [77] foreach_1.4.8           vctrs_0.2.2
#> [79] cellranger_1.1.0        gtable_0.3.0
#> [81] assertthat_0.2.1        xfun_0.12
#> [83] broom_0.5.4             survival_3.1-8
#> [85] viridisLite_0.3.0       iterators_1.0.12
#> [87] IRanges_2.18.3          cluster_2.1.0
```

2 Prepare Data

2.1 Load LMAT read counts and sample data

LMAT maps the reads from shotgun metagenomic sequencing to taxonomic lineages as specifically as possible, according to match scores above a certain threshold. (Briefly, a read must match a *taxid* better than would the best of 1 million random length and GC-matched reads, and the read must match the *taxid* better than it would a parent or sibling *taxid*).

A closed reference based on NCBI taxonomy is used, and we'll focus our analyses only on reads that have been assigned to a microbial genus or species. The function `keep_lmat_microbes` removes *taxids* (and their read counts) containing the kingdom Metazoa or Viridiplantae in their lineage, and it also removes taxons with the word "synthetic" in the species name.

We summarize read count totals at the genus and species levels.

```
#> function (lmat)
#> {
#>     lmat %>% filter(!(kingdom %in% c("Metazoa", "Viridiplantae")) |
#>         is.na(kingdom)) %>% filter(!grepl("synthetic", species))
#> }
```

We have many samples across the flights, but perhaps not so many per location and type.

Table 2.1: sample tally

experiment	type	location	pma_treated	n
crew	control	control_body	no	7
crew	control	NA	no	4
crew	sample	ear	no	8
crew	sample	mouth	no	8
crew	sample	nostril	no	8
crew	sample	saliva	no	31

Table 2.1: sample tally *(continued)*

experiment	type	location	pma_treated	n
crew	sample	skin	no	8
surfaces	control	control_filter	no	2
surfaces	control	control_filter	yes	2
surfaces	control	control_filter_sample	no	2
surfaces	control	control_filter_sample	yes	2
surfaces	control	control_library_ntc	no	1
surfaces	control	control_maxwell	no	1
surfaces	control	control_wipe_flown	no	2
surfaces	control	control_wipe_flown	yes	2
surfaces	control	control_zymo_culture	no	1
surfaces	control	control_zymo_dna	no	2
surfaces	sample	ARED_foot_platform	no	5
surfaces	sample	ARED_foot_platform	yes	5
surfaces	sample	dining_table	no	5
surfaces	sample	dining_table	yes	5
surfaces	sample	lab_overhead_3	no	5
surfaces	sample	lab_overhead_3	yes	5
surfaces	sample	overhead_4	no	5
surfaces	sample	overhead_4	yes	5
surfaces	sample	PMM_port_1	no	2
surfaces	sample	PMM_port_1	yes	2
surfaces	sample	port_crew_quarters	no	5
surfaces	sample	port_crew_quarters	yes	5
surfaces	sample	port_panel	no	5
surfaces	sample	port_panel	yes	5
surfaces	sample	WHC	no	5
surfaces	sample	WHC	yes	5

We store abundance data as a list of nested data frames.

2.2 Mapped reads

We inspect the number of reads (technically read pairs) that map to a genus, species, and any taxonomy ID greater than 1 per sample. The fraction out of the total of microbial reads output by LMAT is also shown. Reads counts that do not map at the genus or

species levels are removed from further analyses (e.g., “unknowns”, or mapping at phyla level only, etc...).

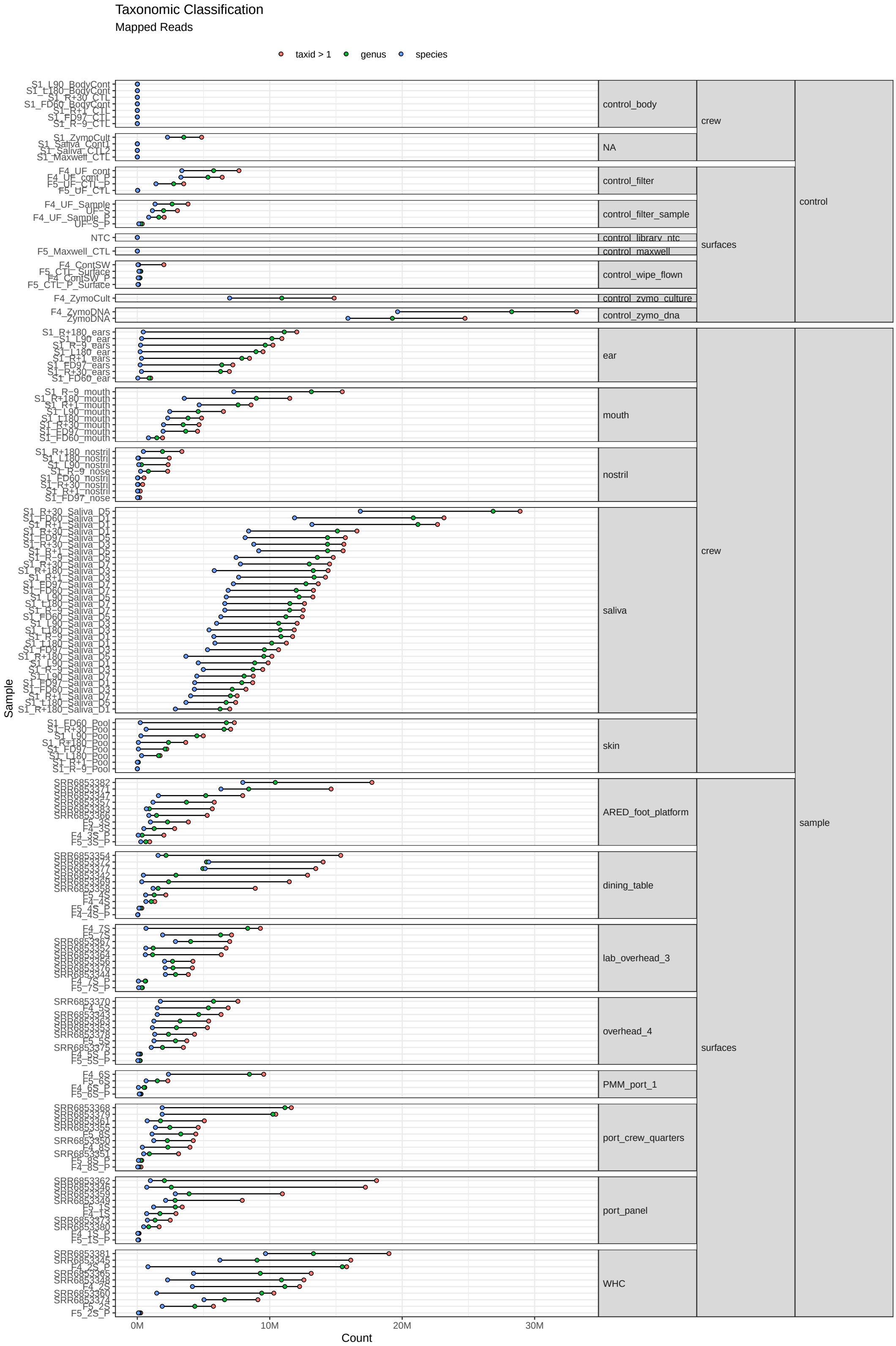
Taxonomic Classification
Mapped Reads
taxid > 1
genus
species
control_body
NA
control_filter
control_filter_sample
control_library_ntc
control_maxwell
control_wipe_flown
control_zymo_culture
control_zymo_dna
ear
mouth
nostril
saliva
skin
ARED_foot_platform
dining_table
lab_overhead_3
overhead_4
PMM_port_1
port_crew_quarters
port_panel
WHC
crew
surfaces
control
sample
Sample
0M
10M
20M
30M
Count

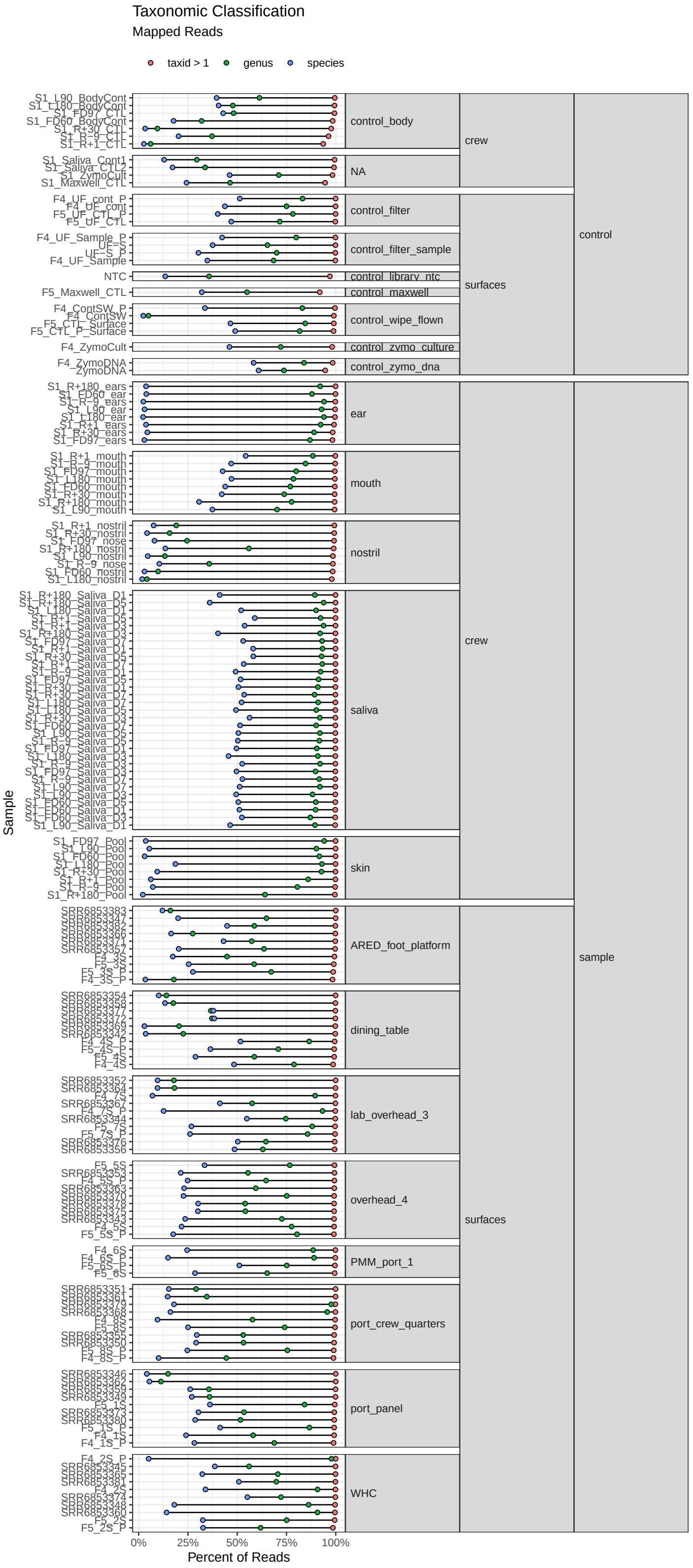
Taxonomic Classification
Mapped Reads
taxid > 1
genus
species
Sample
Percent of Reads
0%
25%
50%
75%
100%
control_body
NA
control_filter
control_filter_sample
control_library_ntc
control_maxwell
control_wipe_flown
control_zymo_culture
control_zymo_dna
ear
mouth
nostril
saliva
skin
ARED_foot_platform
dining_table
lab_overhead_3
overhead_4
PMM_port_1
port_crew_quarters
port_panel
WHC
crew
surfaces
control
sample

2.3 LMAT matchscores

Every read has a matchscore assigned by LMAT. Reads with a matchscore lower than the threshold of 0.5 were removed. A matchscore of 0.5 means that the read's fraction of k-mers that match a reference taxon is $\exp(0.5) = 1.6487213$ times as large as a random read's fraction of matching k-mers (as generated by LMAT's null model).

In the reads which passed the matchscore threshold, taxa seen in most negative control samples were mapped to about as well as those in samples and positive controls (There are microbes detected in the negative controls).

```
#> Picking joint bandwidth of 0.127
```

```
#> Picking joint bandwidth of 0.121
```

```
#> Picking joint bandwidth of 0.109
```

```
#> Picking joint bandwidth of 0.111
```

```
#> Picking joint bandwidth of 0.102
```

```
#> Picking joint bandwidth of 0.0954
```

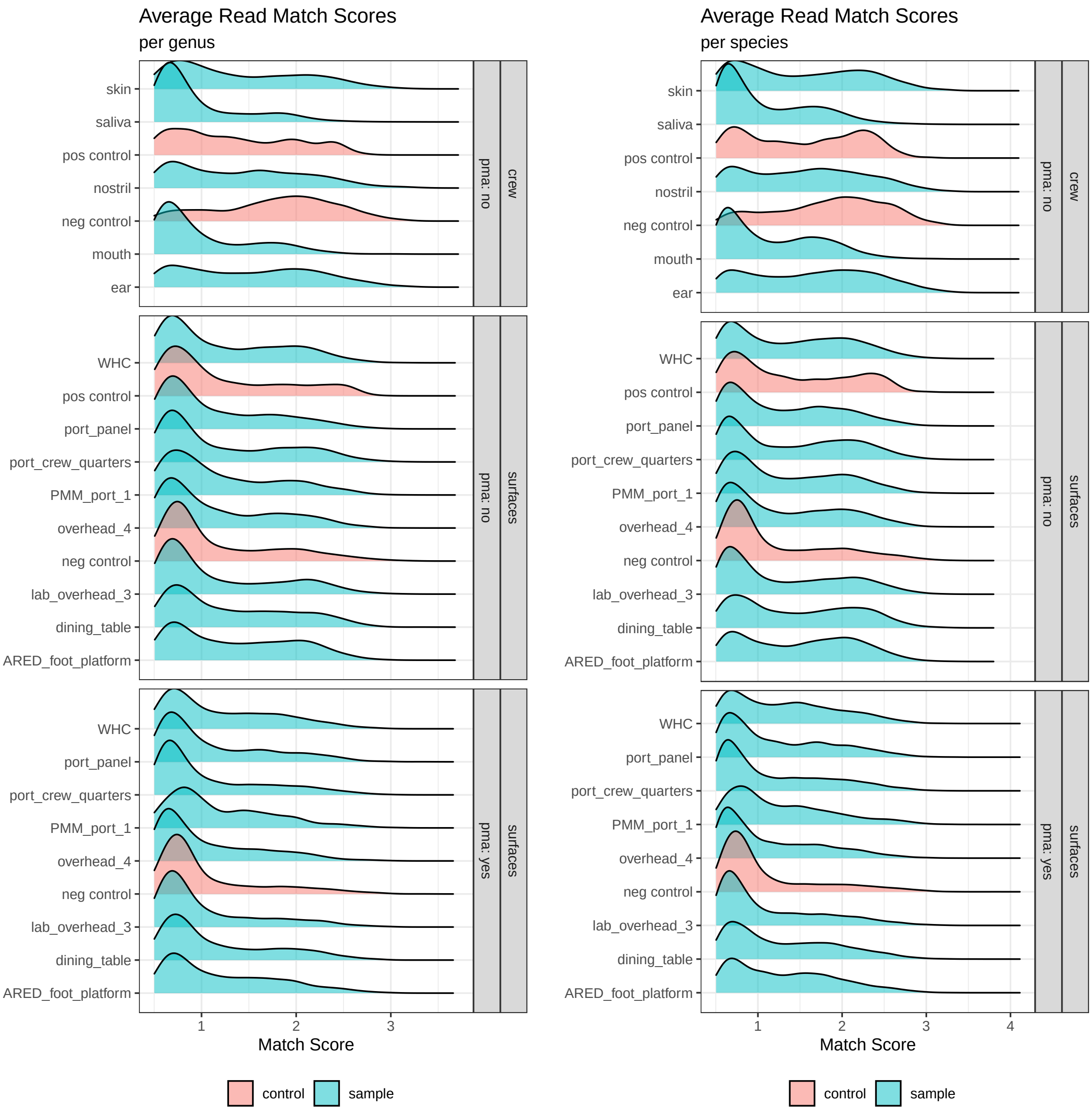
Average Read Match Scores
per genus
skin
saliva
pos control
nostril
neg control
mouth
ear
WHC
port_panel
port_crew_quarters
PMM_port_1
overhead_4
lab_overhead_3
dining_table
ARED_foot_platform
pma: no
pma: yes
crew
surfaces
1
2
3
Match Score
control
sample
Average Read Match Scores
per species
4
Match Score
control
sample

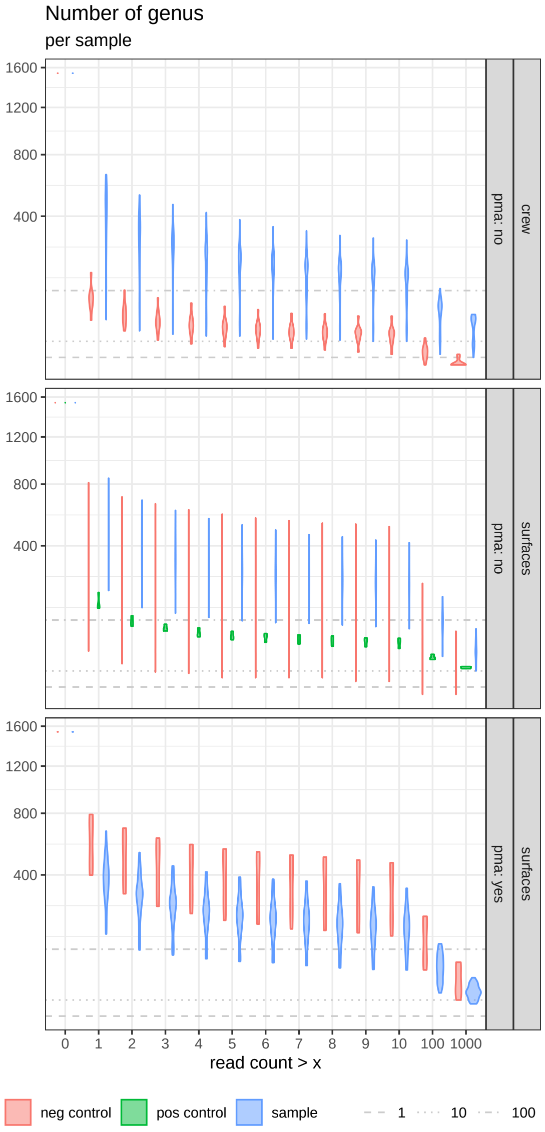

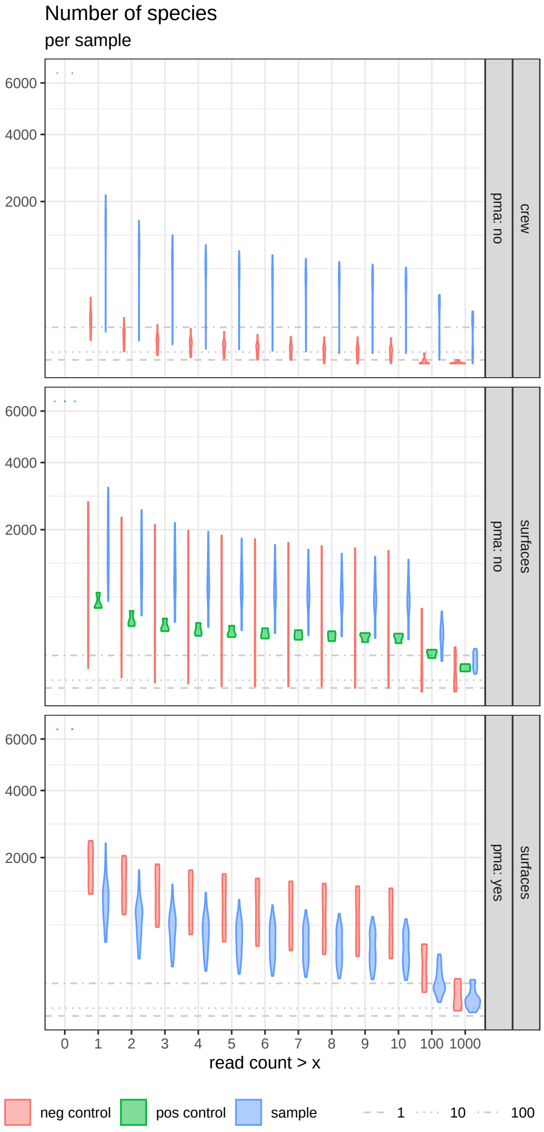

2.3.1 Save data with UNKNOWNs

2.4 CLR monte carlo

For many of the following analyses we will be treating the mapped reads as compositions (parts constrained to sum to a not-very-informative-total). For example, an increase in one species proportion will necessarily decrease others, which potentially leads to spurious Pearson correlations. The centered log ratio (clr) transform is applied to compositional data in order to apply statistical methods developed for unconstrained

data. Species abundances can instead be interpreted with respect to a sample's mean abundance (with the assumption that the mean is a *mean*-ingful reference).

The `aldex2` package samples clr-transformed expected abundances for each sample from a Dirichlet posterior distribution with concentration parameter $\alpha = \texttt{read\_counts} + 0.5$. We then take the mean of the transformed counts as a point estimate for visualization and distance-based analyses. Unmapped read counts were removed prior to the calculation.

The clr transformation is sensitive to removing or adding elements to the composition (i.e., filtering species changes the means, which in turn changes the ratios). We'll remove the UNKNOWNs only.

This next part takes a few minutes.

2.5 Select samples

For the main analyses, we separate samples from controls and make sure unmapped read counts are removed.

2.6 Save

We save read counts in two tables, one for species and one for genera. Unmapped read counts are included, with an "NA" entry for the clr value. We also save the "microbial" only read counts in a third table. These data and the parameters used to run `aldex.clr` are saved as two R objects as well.

3 Prevalence and Abundance

We take a roll call of our tiny stowaways and estimate two parameters of the samples. For our purposes, we define **prevalence** of a taxon as the proportion of samples (of a particular environment or type, e.g., `experiment x pma_treated x location`) in which we detect its presence (usually above a threshold). Relative abundance is the fraction of reads mapped to a taxon in a particular sample. Relative **abundances** are averaged across samples of a particular type or group.

3.1 Geomtric mean

For averaging abundance, we use the geometric mean (GM) because it consistently ranks taxa regardless of which constant is used to "normalize" read counts. In this case, the average proportion of read counts across samples and the average read counts divided by the average "library sizes" should be ranked consistently.

For taxon t with counts $\vec{x}_t$ and library sizes $\vec{L}$:

$$GM\left(\frac{\vec{x}_t}{\vec{L}}\right) = \frac{GM(\vec{x}_t)}{GM(\vec{L})}$$

In compositional data analysis, such means are often normalized to sum to 1 (i.e., the closure of geometric means). These are known as Centers (Cen). For example, if we had 3 taxa in many samples, their centers would look like $\{\mathrm{Cen}(\vec{x}_1), \mathrm{Cen}(\vec{x}_2), \mathrm{Cen}(\vec{x}_3)\}$.

Zero counts are a "problem" for the geometric mean, because a single zero in a sample will drop a taxon's average abundance to zero. Zero's can be addressed by the following methods:

1. Replacing zeros with 1s (as in the `propr` package)
2. Adding a pseudocount to all counts (the `ALDEx2` package uses 0.5)
3. Excluding 0s from the calculation
4. Other fancy techniques

```
#> function (x, method = "discard")
#> {
#>     if (method == "ones") {
```

```
#>         x[x == 0] <- 1
#>     }
#>     else if (method == "discard") {
#>         x <- keep(x, ~.x > 0)
#>     }
#>     else {
#>         x <- x + 0.5
#>     }
#>     if (length(x) == 0 & method == "discard") {
#>         g <- 0
#>     }
#>     else {
#>         g <- exp(mean(log(x)))
#>     }
#>     g
#> }
```

For the tables and figures, we use exclude 0s (method 3) while counting the number of non-zero abundances. For differential abundance analysis via ALDEx2, method 2 is used.

3.2 Groups of samples

Abundances are averaged within various groupings of samples. One investigator might want to rank genera by average abundance within body site. Another investigator might only care about samples taken during flight. Likewise, there are too many taxa to display in plots and choosing a "top N" only makes with respect to the samples displayed. Not to mention, it is possible to rank taxa by additional statistics, e.g., prevalence, log-ratio transformed abundances…

3.3 Top taxa

Color palettes max out at around 12 useful colors, arguably fewer.

We save a few top tables sorted by Cen(proportion)

```
#> Adding missing grouping variables: `experiment`, `pma_treated`, `flight_status`
#> Adding missing grouping variables: `experiment`, `pma_treated`, `flight_status`
```

3.4 Proportion bars

Behold the proportion of reads from the top 12 taxa by Cen(proportion) out of the top 12 taxa per group by mean clr transformed abundance.

```
#> Joining, by = c("pma_treated", "location", "experiment", "genus")
```

```
#> Joining, by = c("pma_treated", "location", "experiment", "species")
```

```
#> Removing pma treated samples
```

```
#> Joining, by = c("pma_treated", "location", "experiment", "genus")
```

```
#> Removing pma treated samples
```

```
#> Joining, by = c("pma_treated", "location", "experiment", "species")
```

```
#> Removing pma treated samples
```

```
#> Joining, by = c("pma_treated", "location", "experiment", "genus")
```

```
#> Removing pma treated samples
```

```
#> Joining, by = c("pma_treated", "location", "experiment", "species")
```

3.4.1 Crew

3.4.1.1 genus

```
#> Warning in RColorBrewer::brewer.pal(n, pal): n too large, allowed maximum for palette Set3
#> Returning the palette you asked for with that many colors
```

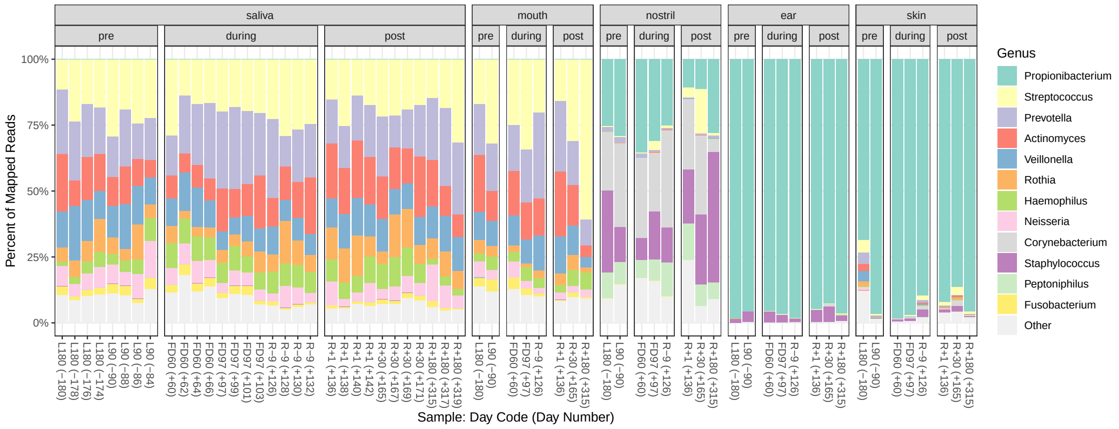

3.4.1.2 species

```
#> Warning in RColorBrewer::brewer.pal(n, pal): n too large, allowed maximum for pa
#> Returning the palette you asked for with that many colors
```

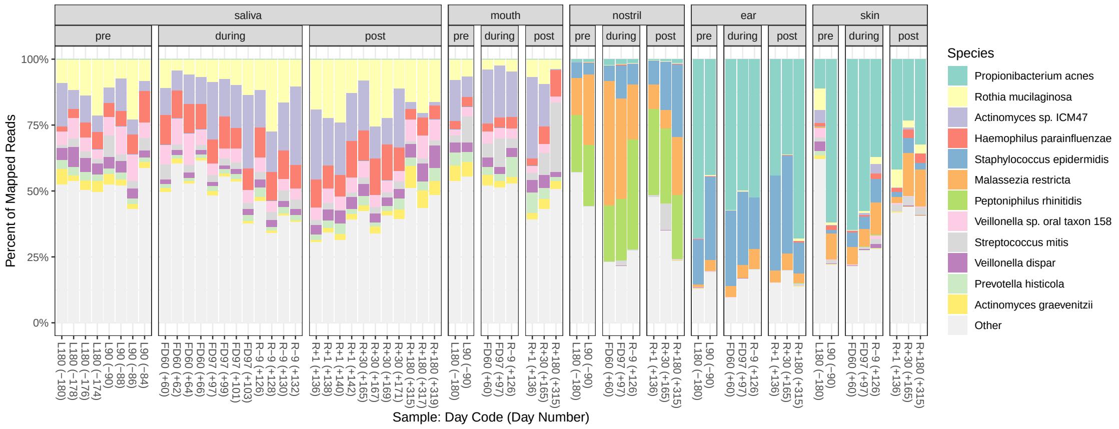


```
#> Warning in RColorBrewer::brewer.pal(n, pal): n too large, allowed maximum for pa
#> Returning the palette you asked for with that many colors

#> Warning in RColorBrewer::brewer.pal(n, pal): n too large, allowed maximum for pa
#> Returning the palette you asked for with that many colors
```

3.4.2 Surfaces

No PMA

3.4.2.1 genus

```
#> Warning in RColorBrewer::brewer.pal(n, pal): n too large, allowed maximum for palette Set3
#> Returning the palette you asked for with that many colors
```

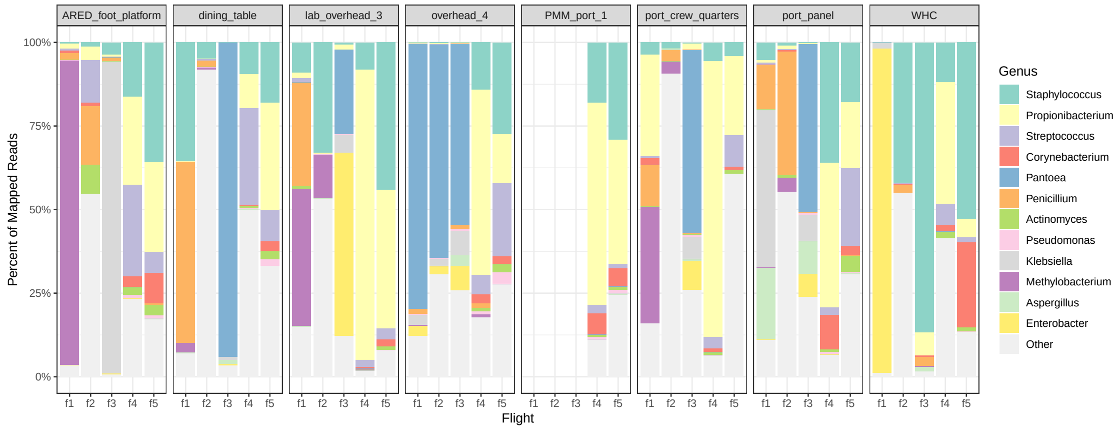

3.4.2.2 species

```
#> Warning in RColorBrewer::brewer.pal(n, pal): n too large, allowed maximum for palette Set3
#> Returning the palette you asked for with that many colors
```

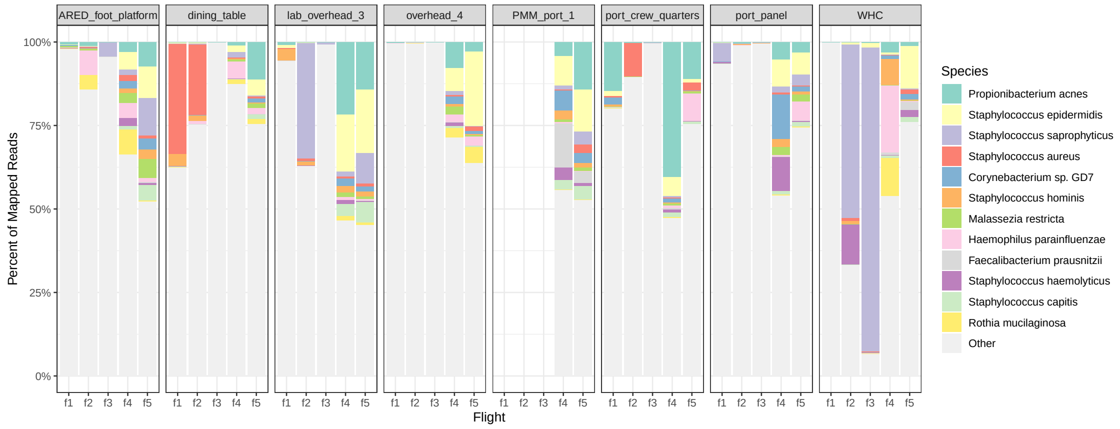


```
#> Warning in RColorBrewer::brewer.pal(n, pal): n too large, allowed maximum for palette Set3
#> Returning the palette you asked for with that many colors
```

```
#> Warning in RColorBrewer::brewer.pal(n, pal): n too large, allowed maximum for palette Set3
#> Returning the palette you asked for with that many colors
```

```
#> Warning in RColorBrewer::brewer.pal(n, pal): n too large, allowed maximum for pa
#> Returning the palette you asked for with that many colors
```

3.5 MT1 vs MT2

Sample location ignored.

```
#> Joining, by = c("taxon", "pma_treated")
#> Joining, by = c("taxon", "pma_treated")
```

3.5.1 PMA

	Flights 1–3				Flights 4, 5			
genus	rank	Cen(p) [%]	$\overline{\text{clr}(p)}$	Prev. [%]	rank	Cen(p) [%]	$\overline{\text{clr}(p)}$	Prev. [%]
Penicillium	1	33.28	1629.82	100.00	60	0.03	670.89	100.00
Staphylococcus	2	22.84	1575.67	100.00	2	16.09	1583.76	100.00
Methylobacterium	3	5.87	1302.99	95.24	12	0.39	1046.07	100.00
Pantoea	4	2.83	1274.37	100.00	123	0.01	438.83	100.00
Lecanosticta	5	2.62	1263.36	100.00	424	0.00	3.35	37.50
Propionibacterium	6	1.72	1201.87	100.00	1	63.66	1782.07	100.00
Klebsiella	7	1.58	1192.76	100.00	42	0.06	779.29	100.00
Aspergillus	8	1.60	1191.50	100.00	55	0.04	716.91	100.00
Rhodotorula	9	1.45	1177.85	100.00	293	0.00	117.45	43.75
Enterobacter	10	1.37	1168.17	100.00	39	0.08	809.15	100.00
Puccinia	11	0.83	1096.77	100.00	41	0.06	782.38	100.00
Pseudomonas	12	0.68	1069.08	100.00	5	1.13	1200.94	100.00
Rhodosporidium	13	0.50	1024.74	100.00	416	0.01	8.27	25.00
Paenibacillus	14	0.41	997.22	100.00	75	0.02	617.40	100.00
Escherichia	15	0.56	976.34	95.24	66	0.03	655.22	100.00
Streptococcus	16	0.32	957.18	100.00	3	3.56	1365.92	100.00
Acinetobacter	19	0.23	911.38	100.00	8	0.67	1125.34	100.00
Corynebacterium	21	0.14	836.38	100.00	4	2.61	1321.35	100.00
Malassezia	38	0.05	685.96	100.00	7	0.73	1137.24	100.00
Haemophilus	46	0.03	602.26	100.00	14	0.33	1024.81	100.00
Actinomyces	47	0.07	589.29	80.95	6	1.08	1193.38	100.00
Veillonella	50	0.02	580.44	100.00	9	0.56	1099.15	100.00
Prevotella	56	0.02	549.74	100.00	10	0.53	1092.17	100.00
Neisseria	58	0.02	535.89	95.24	15	0.32	1016.94	100.00
Rothia	92	0.03	430.96	76.19	11	0.47	1073.75	100.00
Lactococcus	180	0.00	273.53	90.48	13	0.37	1041.01	100.00

	Flights 1–3				Flights 4, 5			
species	rank	Cen(p) [%]	$\overline{\mathrm{clr}(p)}$	Prev. [%]	rank	Cen(p) [%]	$\overline{\mathrm{clr}(p)}$	Prev. [%]
Penicillium chrysogenum	1	2.66	1381.18	100.00	1533	0.01	26.63	31.25
Lecanosticta acicola	2	1.80	1323.86	100.00	1312	0.01	57.29	37.50
Penicillium fuscoglaucum	3	1.16	1261.37	100.00	1607	0.00	18.22	25.00
Rhodotorula glutinis	4	1.00	1239.20	100.00	789	0.02	163.17	43.75
Penicillium nalgiovense	5	0.68	1184.63	100.00	1738	0.01	3.34	18.75
Staphylococcus saprophyticus	6	1.63	1172.15	90.48	10	1.05	1020.44	100.00
Puccinia striiformis	7	0.47	1129.02	100.00	89	0.09	657.63	100.00
Rhodosporidium toruloides	8	0.35	1085.92	100.00	1284	0.04	61.70	25.00
Klebsiella pneumoniae	9	0.85	1053.44	85.71	137	0.05	572.56	100.00
Staphylococcus epidermidis	10	0.22	1020.98	100.00	2	7.59	1305.54	100.00
Staphylococcus aureus	11	0.30	1019.64	95.24	8	1.12	1029.71	100.00
Penicillium roqueforti	12	0.23	973.22	95.24	2158	0.02	-26.66	12.50
Penicillium biforme	13	0.20	958.21	95.24	2365	0.02	-40.87	12.50
Penicillium digitatum	14	0.77	953.92	80.95	325	0.03	388.12	81.25
Aspergillus niger	15	0.14	911.03	95.24	672	0.01	201.61	62.50
Staphylococcus hominis	18	0.09	888.93	100.00	6	1.19	1038.51	100.00
Propionibacterium acnes	22	0.07	854.85	100.00	1	17.87	1429.10	100.00
Staphylococcus capitis	58	0.04	647.83	85.71	4	2.49	1144.35	100.00
Malassezia restricta	74	0.01	602.57	100.00	3	2.68	1155.52	100.00
Staphylococcus sp. AL1	80	0.03	590.95	80.95	15	0.70	962.02	100.00
Corynebacterium sp. GD7	138	0.01	496.07	85.71	5	1.75	1094.56	100.00
Haemophilus parainfluenzae	152	0.01	486.70	90.48	7	1.13	1030.88	100.00
Streptococcus mitis	191	0.01	449.32	85.71	9	1.05	1020.94	100.00
Rothia mucilaginosa	203	0.01	435.85	76.19	13	0.80	981.45	100.00
Streptococcus sanguinis	213	0.01	426.56	80.95	14	0.74	969.31	100.00
Micrococcus luteus	431	0.01	292.78	66.67	12	0.81	982.21	100.00
Lactococcus lactis	515	0.00	251.49	71.43	11	0.83	986.76	100.00

3.5.2 No PMA

	Flights 1–3				Flights 4, 5			
genus	rank	Cen(p) [%]	$\overline{\mathrm{clr}(p)}$	Prev. [%]	rank	Cen(p) [%]	$\overline{\mathrm{clr}(p)}$	Prev. [%]
Staphylococcus	1	27.20	1628.25	100.00	2	26.58	1914.54	100.00
Penicillium	2	22.79	1602.73	100.00	64	0.02	847.84	100.00
Propionibacterium	3	11.86	1508.44	100.00	1	46.64	1995.67	100.00
Methylobacterium	4	5.25	1390.63	100.00	44	0.04	987.68	100.00
Streptococcus	5	2.66	1292.82	100.00	3	9.07	1759.34	100.00
Pantoea	6	2.43	1280.16	100.00	82	0.01	766.70	100.00
Klebsiella	7	1.94	1248.18	100.00	58	0.02	887.81	100.00
Lecanosticta	8	1.87	1242.04	100.00	290	0.00	233.14	93.75
Aspergillus	9	1.61	1220.10	100.00	68	0.01	828.12	100.00
Enterobacter	10	1.56	1215.47	100.00	54	0.03	911.26	100.00
Corynebacterium	11	1.27	1186.41	100.00	4	4.22	1649.06	100.00
Actinomyces	12	1.03	1155.98	100.00	5	1.87	1531.81	100.00
Puccinia	13	0.78	1116.09	100.00	43	0.04	988.55	100.00
Rhodotorula	14	0.54	1063.84	100.00	200	0.00	379.33	87.50
Pseudomonas	15	0.53	1059.50	100.00	13	0.49	1338.90	100.00
Veillonella	18	0.34	995.74	100.00	8	1.09	1454.37	100.00
Malassezia	19	0.31	984.17	100.00	9	0.69	1386.93	100.00
Haemophilus	20	0.30	978.12	100.00	6	1.20	1467.60	100.00
Neisseria	22	0.31	922.73	95.24	7	1.13	1458.87	100.00
Rothia	23	0.20	919.09	100.00	10	0.67	1383.01	100.00
Prevotella	36	0.06	753.82	100.00	11	0.56	1357.77	100.00
Gemella	37	0.05	729.36	100.00	12	0.49	1339.18	100.00
Lautropia	45	0.08	687.09	90.48	15	0.37	1297.45	100.00
Fusobacterium	56	0.03	639.96	100.00	14	0.41	1313.87	100.00

	Flights 1–3				Flights 4, 5			
species	rank	Cen(p) [%]	$\overline{\text{clr}(p)}$	Prev. [%]	rank	Cen(p) [%]	$\overline{\text{clr}(p)}$	Prev. [%]
Penicillium chrysogenum	1	2.70	1333.75	100.00	867	0.00	290.09	87.50
Lecanosticta acicola	2	2.17	1302.36	100.00	873	0.00	286.04	93.75
Propionibacterium acnes	3	1.31	1229.65	100.00	2	14.14	1654.07	100.00
Staphylococcus saprophyticus	4	1.26	1223.59	100.00	6	2.30	1392.11	100.00
Penicillium fuscoglaucum	5	1.26	1223.51	100.00	1254	0.00	155.22	68.75
Klebsiella pneumoniae	6	0.86	1168.33	100.00	266	0.02	700.98	100.00
Staphylococcus epidermidis	7	0.84	1165.93	100.00	1	15.35	1665.99	100.00
Staphylococcus aureus	8	0.76	1152.53	100.00	13	1.51	1331.10	100.00
Penicillium nalgiovense	9	0.76	1151.62	100.00	1356	0.00	133.28	68.75
Rhodotorula glutinis	10	0.63	1124.71	100.00	593	0.01	432.33	87.50
Puccinia striiformis	11	0.58	1112.83	100.00	179	0.04	816.04	100.00
Staphylococcus hominis	12	0.37	1046.38	100.00	9	2.10	1379.31	100.00
Penicillium digitatum	13	0.43	1013.16	95.24	343	0.01	623.84	100.00
Aspergillus niger	14	0.38	994.35	95.24	627	0.00	408.42	93.75
Malassezia restricta	15	0.22	971.47	100.00	7	2.22	1386.95	100.00
Haemophilus parainfluenzae	16	0.22	970.53	100.00	3	3.80	1464.47	100.00
Corynebacterium sp. GD7	20	0.17	936.46	100.00	4	3.59	1456.19	100.00
Rothia mucilaginosa	21	0.17	935.73	100.00	14	1.48	1328.94	100.00
Streptococcus sanguinis	31	0.15	876.74	95.24	12	1.58	1337.58	100.00
Staphylococcus capitis	34	0.09	837.61	100.00	5	3.32	1445.19	100.00
Streptococcus mitis	45	0.06	776.76	100.00	8	2.16	1383.06	100.00
Lautropia mirabilis	54	0.09	747.69	90.48	10	1.71	1349.23	100.00
Staphylococcus caprae	96	0.03	640.11	95.24	15	1.25	1304.10	100.00
Staphylococcus pettenkoferi	131	0.04	571.54	80.95	11	1.66	1345.39	100.00

3.6 Caveats

1. We must keep in mind that genome sizes vary between and even within species, and that cells may contain multiple genome copies (polyploidy). Cellular and DNA abundance as measured by read counts are not equivalent. Cells preparing for division complicate estimates by replicating DNA near origins of replication.
2. Additionally, read counts are "count-compositional" data because they are whole numbers constrained to sum to a total (library size) that is typically limited by the instrument and non-trivially affected by sample preparation and chance. In other words, they may be treated as proportions, specifically as proportions of detectable taxa, with the understanding that information about precision of the measurement and variance may be lost in the division and subsequent transformations.
3. Taxon read counts may also be modeled as discrete counts from negative binomial (gamma poisson) distributions. However, in both cases, mispecifying the model has been shown to inflate the false discovery rate.
4. *Propionibacterium* has been reorganized into 4 genera including *Cutibacterium*.

4 Alpha diversity

4.1 Richness and Evenness

Richness and evenness of taxa describe compositional diversity. We measure it in our samples and use these measurements to estimate the diversity of an environment, perhaps over time.

Richness can be used to investigate "How many species does this environment support?" Evenness asks "Are all species equally dominant?"

Common diversity indices such as richness, Shannon, and Simpson indices correspond to special cases of generalized weighted means and Renyí entropies of degrees 0, 1, and 2 respectively and Hill numbers N_0, N_1, N_2.

We use `vegan::renyi(hill = TRUE)` to estimate Hill numbers which represent the expected "effective" number of species according to their abundances.

4.2 N_1 vs. mapped microbial reads

Sequencing deeper may detect more low abundance species. Additionally, score and abundance thresholds, and mapping approach, and reference databases affect how species are counted.

4.2.1 Crew

```
#> `geom_smooth()` using method = 'loess' and formula 'y ~ x'
#> `geom_smooth()` using method = 'loess' and formula 'y ~ x'
#> `geom_smooth()` using method = 'loess' and formula 'y ~ x'
```

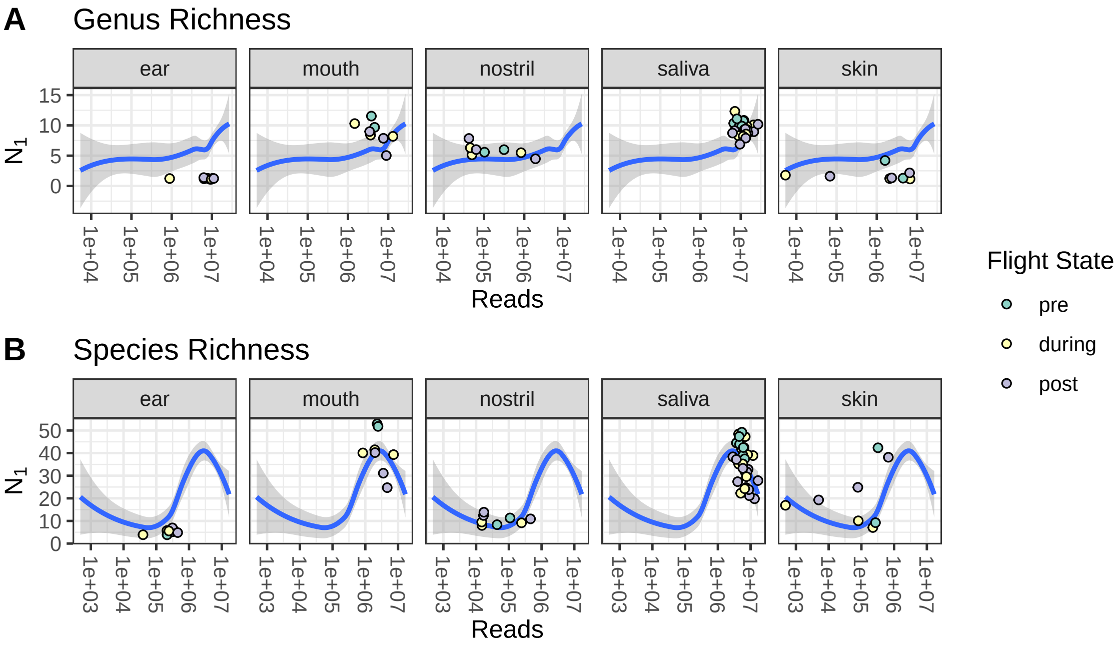

4.2.2 Surfaces

```
#> `geom_smooth()` using method = 'loess' and formula 'y ~ x'
#> `geom_smooth()` using method = 'loess' and formula 'y ~ x'
#> `geom_smooth()` using method = 'loess' and formula 'y ~ x'
```

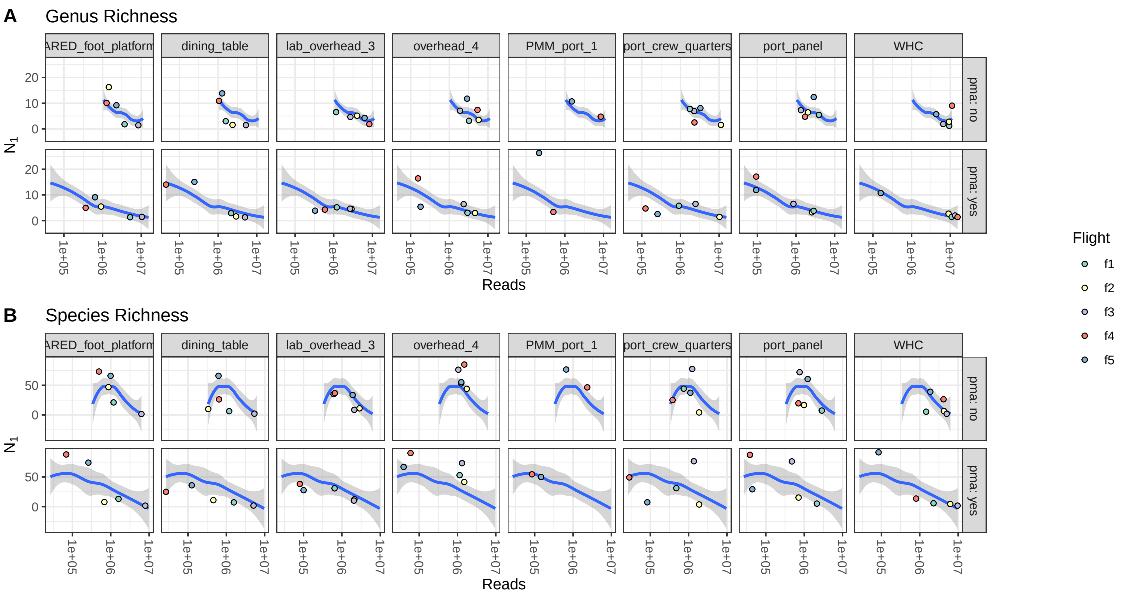

4.3 Effective counts of taxa

Hill numbers capture evenness by weighting counts by taxon proportion [1]: $\exp(\text{Shannon}) = N_1$, $\text{Simpson}^{-1} = N_2$.

In this way, evenness can also be framed as counts of taxa that are weighted by their relative abundances. A perfectly uniformly distributed sample will equally weight all taxa, whereas a skewed sample will downweight rare taxa, resulting in smaller effective counts.

4.3.1 Crew

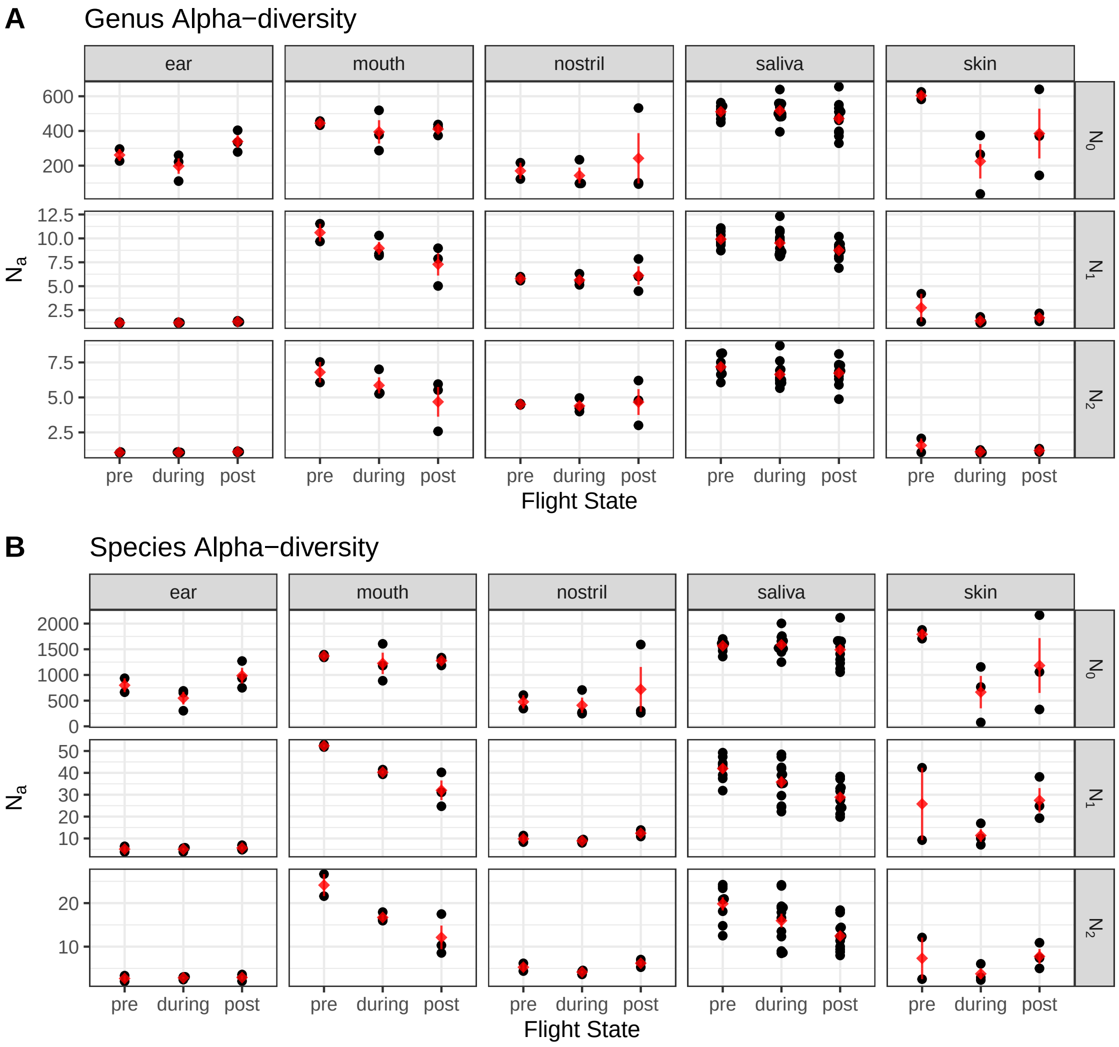

4.3.2 Surfaces

```
#> Warning: Removed 111 rows containing missing values (geom_pointrange).
```

```
#> Warning: Removed 111 rows containing missing values (geom_pointrange).
```

```
#> Warning: Removed 111 rows containing missing values (geom_pointrange).
```

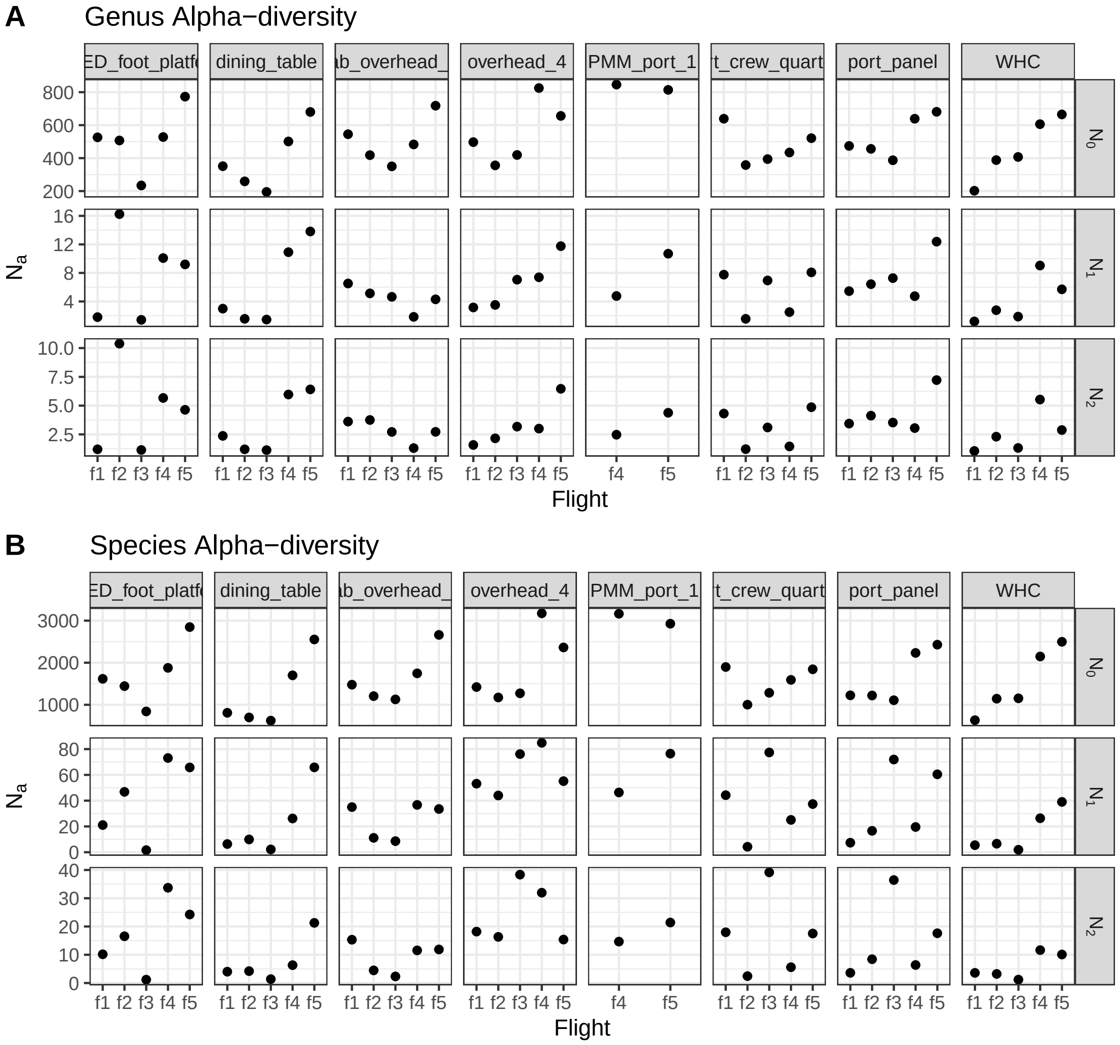


```
#> Warning: Removed 111 rows containing missing values (geom_pointrange).
```

```
#> Warning: Removed 111 rows containing missing values (geom_pointrange).
```

```
#> Warning: Removed 111 rows containing missing values (geom_pointrange).
```

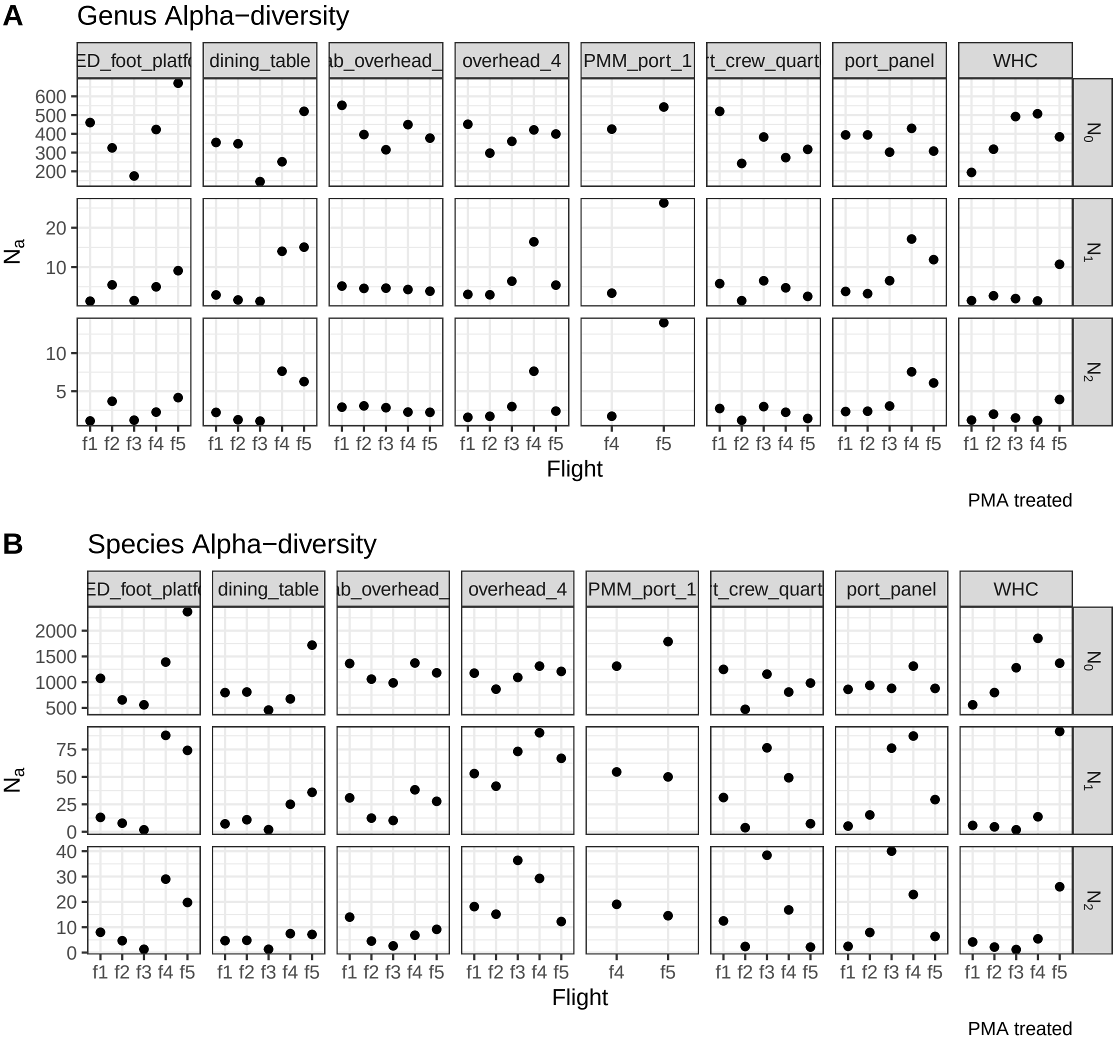

4.4 Saliva α-diversity

```
#> Warning: Removed 93 rows containing missing values (geom_pointrange).
```

```
#> Warning: Removed 93 rows containing missing values (geom_pointrange).
```

```
#> Warning: Removed 93 rows containing missing values (geom_pointrange).
```

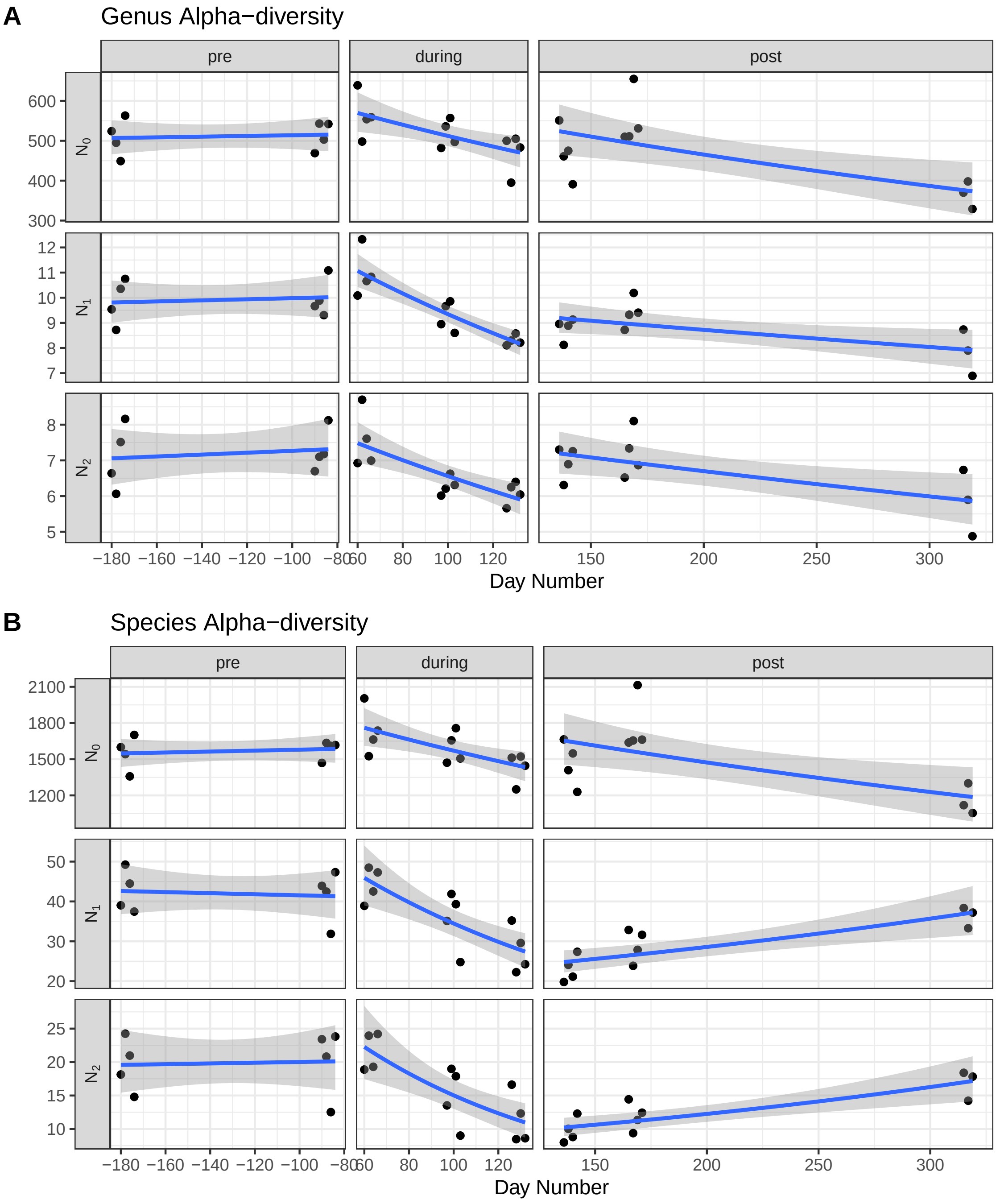


```
#> Warning: Removed 93 rows containing missing values (geom_pointrange).
```

4.4.1 Species N_1

```
#> Warning: Removed 31 rows containing missing values (geom_pointrange).
```

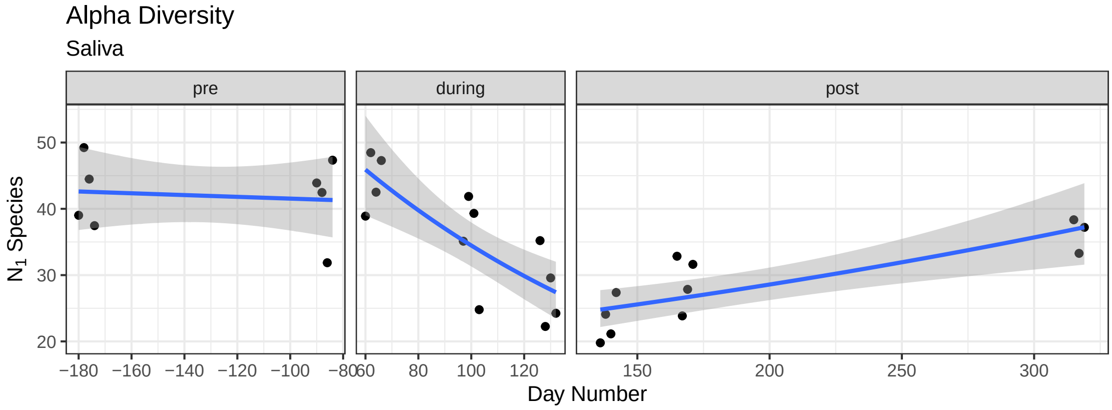


```
#> Warning: Removed 31 rows containing missing values (geom_pointrange).
```

4.4.2 Test effect of `day_number` and `sum_reads`

We fit a model for each flight state (pre, during, post) for each alpha diversity measure (N_0, N_1, N_2) of genus and species read counts (3 x 3 x 2 = 18 models). We fit a Gamma GLM with a log link function because the Hill numbers are non-negative. We include the day number and the number of mapped reads as predictors of Hill number.

For each model, we compare it to a model which drops either term by visually inspectng the change in effect sizes and performing a likelihood ratio test to identify terms that "significantly" affect model fit.

```
#> # A tibble: 18 x 5
#>    tax_rank flight_status a     data              model
#>    <chr>    <fct>         <chr> <list>            <list>
#>  1 genus    during        N_0   <tibble [12 x 3]> <glm>
#>  2 genus    pre           N_0   <tibble [8 x 3]>  <glm>
#>  3 genus    post          N_0   <tibble [11 x 3]> <glm>
#>  4 species  during        N_0   <tibble [12 x 3]> <glm>
#>  5 species  pre           N_0   <tibble [8 x 3]>  <glm>
#>  6 species  post          N_0   <tibble [11 x 3]> <glm>
#>  7 genus    during        N_1   <tibble [12 x 3]> <glm>
#>  8 genus    pre           N_1   <tibble [8 x 3]>  <glm>
#>  9 genus    post          N_1   <tibble [11 x 3]> <glm>
#> 10 species  during        N_1   <tibble [12 x 3]> <glm>
#> 11 species  pre           N_1   <tibble [8 x 3]>  <glm>
#> 12 species  post          N_1   <tibble [11 x 3]> <glm>
#> 13 genus    during        N_2   <tibble [12 x 3]> <glm>
#> 14 genus    pre           N_2   <tibble [8 x 3]>  <glm>
```

```
#> 15 genus    post          N_2   <tibble [11 x 3]> <glm>
#> 16 species  during        N_2   <tibble [12 x 3]> <glm>
#> 17 species  pre           N_2   <tibble [8 x 3]>  <glm>
#> 18 species  post          N_2   <tibble [11 x 3]> <glm>
```

```
#> Joining, by = c("tax_rank", "flight_status", "a", "term")
```

4.4.2.1 Fitted vs. Observed

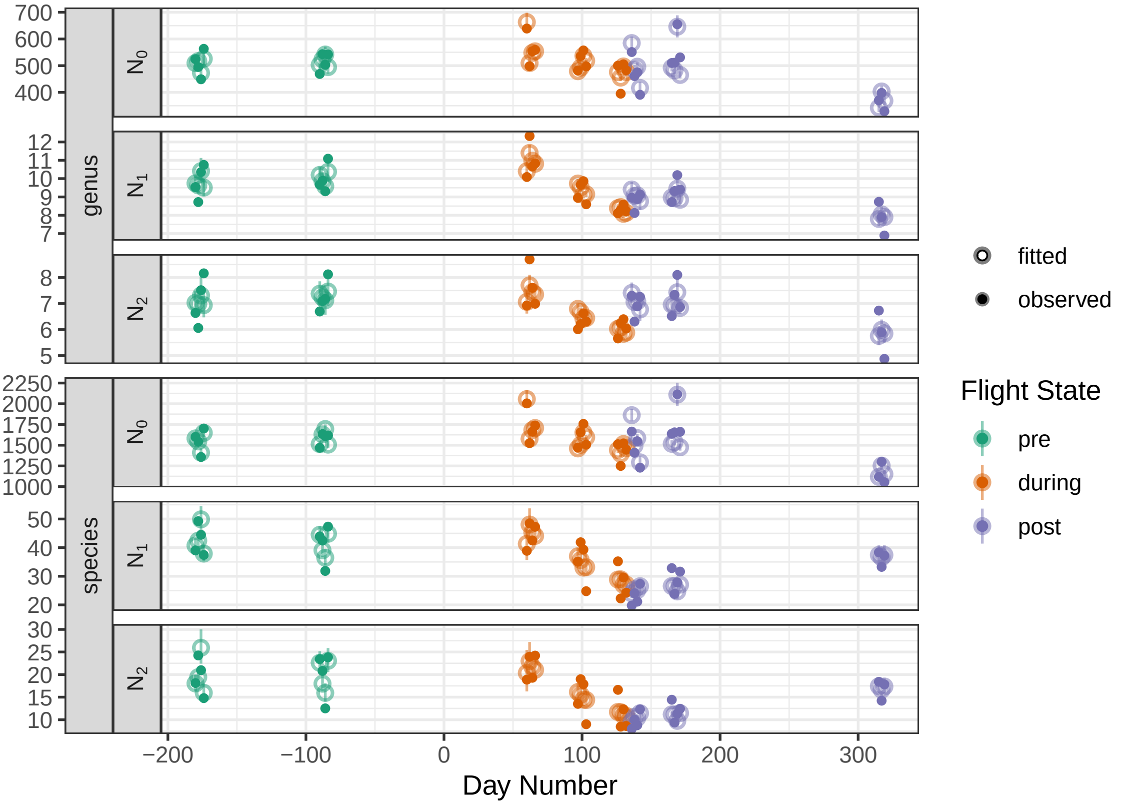

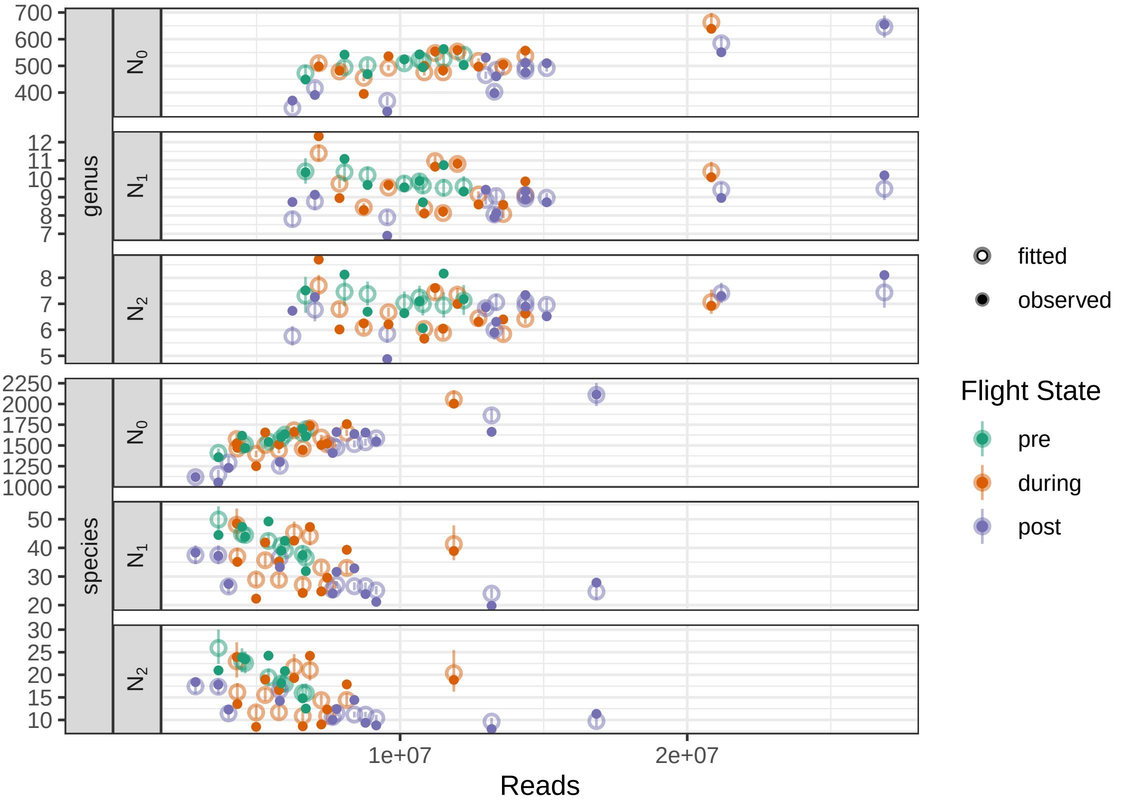

tax_rank	flight_status	a	term	estimate	std.error	p.value	padj
**species**	**during**	**N_2**	**day_number**	**-0.0100868**	**0.0028842**	**0.0004351**	**0.0019581**
**species**	**during**	**N_1**	**day_number**	**-0.0074704**	**0.0018885**	**0.0000755**	**0.0005436**
**genus**	**during**	**N_1**	**day_number**	**-0.0043703**	**0.0006317**	**0.0000000**	**0.0000000**
**genus**	**during**	**N_2**	**day_number**	**-0.0034245**	**0.0008426**	**0.0000338**	**0.0003042**
species	post	N_2	day_number	0.0023132	0.0008397	0.0057506	0.0172518
**species**	**during**	**N_0**	**day_number**	**-0.0022024**	**0.0006643**	**0.0009746**	**0.0038986**
**genus**	**during**	**N_0**	**day_number**	**-0.0021598**	**0.0006629**	**0.0011488**	**0.0041356**
species	post	N_1	day_number	0.0019303	0.0007405	0.0091799	0.0236055
genus	post	N_0	day_number	-0.0010259	0.0003734	0.0070713	0.0195821
genus	post	N_2	day_number	-0.0009119	0.0004783	0.0592297	0.1254275
genus	post	N_1	day_number	-0.0006549	0.0003873	0.0942377	0.1884755
species	post	N_0	day_number	-0.0005777	0.0004076	0.1604036	0.2887264
genus	pre	N_2	day_number	0.0003839	0.0009237	0.6778374	0.7624761
species	pre	N_1	day_number	-0.0003230	0.0009025	0.7201163	0.7624761
species	pre	N_0	day_number	0.0002473	0.0003720	0.5056763	0.6277361
genus	pre	N_1	day_number	0.0002388	0.0006543	0.7150514	0.7624761
species	pre	N_2	day_number	0.0001928	0.0014758	0.8959953	0.8959953
genus	pre	N_0	day_number	0.0001636	0.0006125	0.7889769	0.8115191
species	pre	N_2	sum_reads	-0.0000002	0.0000001	0.0169357	0.0406457
species	pre	N_1	sum_reads	-0.0000001	0.0000000	0.0239615	0.0539133
**species**	**pre**	**N_0**	**sum_reads**	**0.0000001**	**0.0000000**	**0.0014777**	**0.0048362**
**species**	**post**	**N_0**	**sum_reads**	**0.0000000**	**0.0000000**	**0.0000002**	**0.0000030**
**species**	**during**	**N_0**	**sum_reads**	**0.0000000**	**0.0000000**	**0.0001528**	**0.0009168**
**genus**	**post**	**N_0**	**sum_reads**	**0.0000000**	**0.0000000**	**0.0000013**	**0.0000153**
genus	pre	N_0	sum_reads	0.0000000	0.0000000	0.1706297	0.2925081
species	during	N_1	sum_reads	0.0000000	0.0000000	0.4040761	0.5387681
genus	pre	N_1	sum_reads	0.0000000	0.0000000	0.2619558	0.4100178
**genus**	**during**	**N_0**	**sum_reads**	**0.0000000**	**0.0000000**	**0.0002712**	**0.0013947**
species	during	N_2	sum_reads	0.0000000	0.0000000	0.6444753	0.7484230
species	post	N_2	sum_reads	0.0000000	0.0000000	0.2493253	0.4079869
genus	pre	N_2	sum_reads	0.0000000	0.0000000	0.6402878	0.7484230
species	post	N_1	sum_reads	0.0000000	0.0000000	0.4751278	0.6108786
genus	during	N_1	sum_reads	0.0000000	0.0000000	0.1369345	0.2594549
genus	during	N_2	sum_reads	0.0000000	0.0000000	0.3012445	0.4518668
genus	post	N_2	sum_reads	0.0000000	0.0000000	0.3254293	0.4686182
genus	post	N_1	sum_reads	0.0000000	0.0000000	0.3423242	0.4739874

4.4.3 Sensitivity of estimate

```
#> Joining, by = c("tax_rank", "flight_status", "a", "term")
```

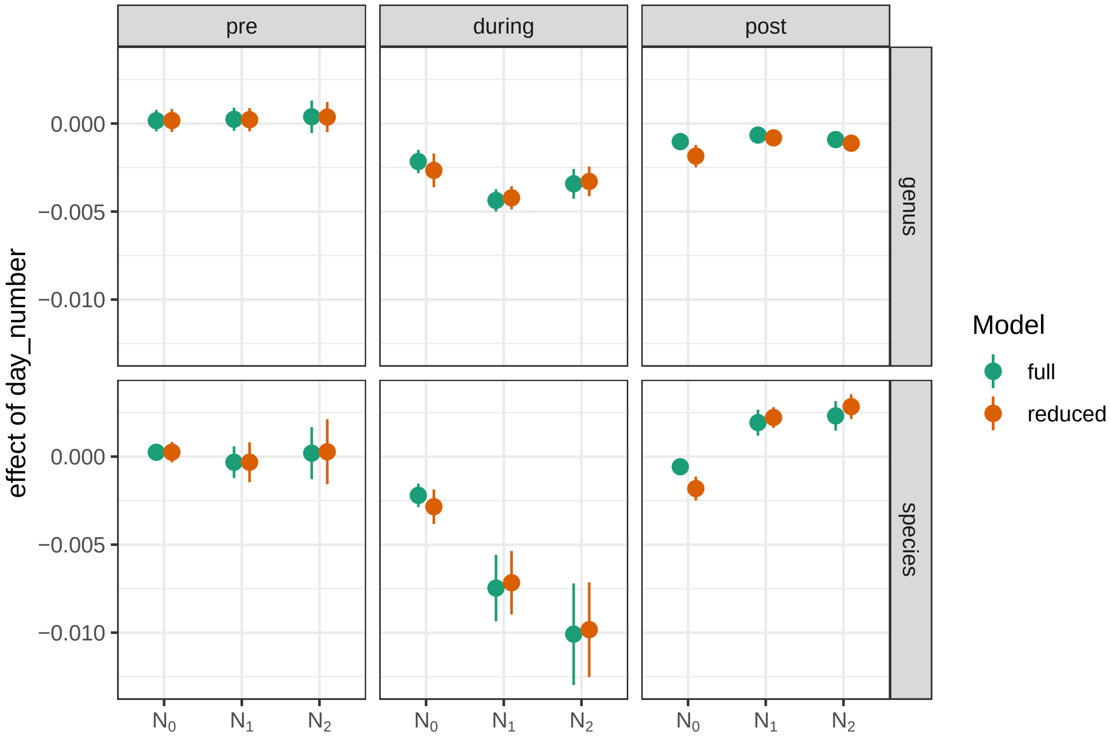

4.5 Caveats

Typically, taxon presence-absence and relative abundances are plugged into formulas for "true α-diversity" (See Amy Willis' work on estimating diversity).

This assumes taxa are interchangeable (i.e., phylogenetic relationships between all taxa are equivalent and that taxa behave independently). The diversity statistics are calculated per-sample and we attempt to generalize these findings to environments.

In the future, we are eager to explore methods that account for phylogenetic relationships, and non-independence between taxa such as DivNet.

Estimates of α-diversity may be affected by sequencing effort. Samples which yielded fewer reads might miss less abundant species. **Species with relatively larger genomes may appear more abundant**. Additionally, database biases and mapping accuracy affect the recruitment of reads which are then used as plug-in frequencies to these calculations. Abundant species are also likely to result in highly variable measurements as there is an overdispersed mean-variance relationship in read count data.

5 Beta-diversity

See Nick's code for inspiration: `20180906_diversity_code.r`, `20180913_distance_jaccard_code.r`

5.1 Ecological distances

Beta-diversity is about how similar or different microbial compositions are between environments.

We estimate distances using pairs of samples from within and between environments. At course-grain level, samples can be thought of as binary vectors with an entry for each taxon, 1 = present, 0 = absent. We can then use the Jaccard distance or "Intersection over Union" of two sets.

We can visualize whether groups of samples from similar environments cluster together using ordination methods, and perform tests for significant ecological distances (or dissimilarities) between groups (e.g., PERMANOVA).

Additionally, we can incorporate relative abundances into our distance metric, taking care to transform relative abundances to be relative to the mean abundance rather than the sum, and to choose ordination methods for the distances/dissimilarities we have computed. An alternative approach is to test for differential abundance of each taxon between conditions as with ALDEx2.

We now take advantage of the great `phyloseq` package.

5.2 Ordination

5.2.1 Skin-surfaces Distances

We visualize the raw pairwise distances within and between groups.

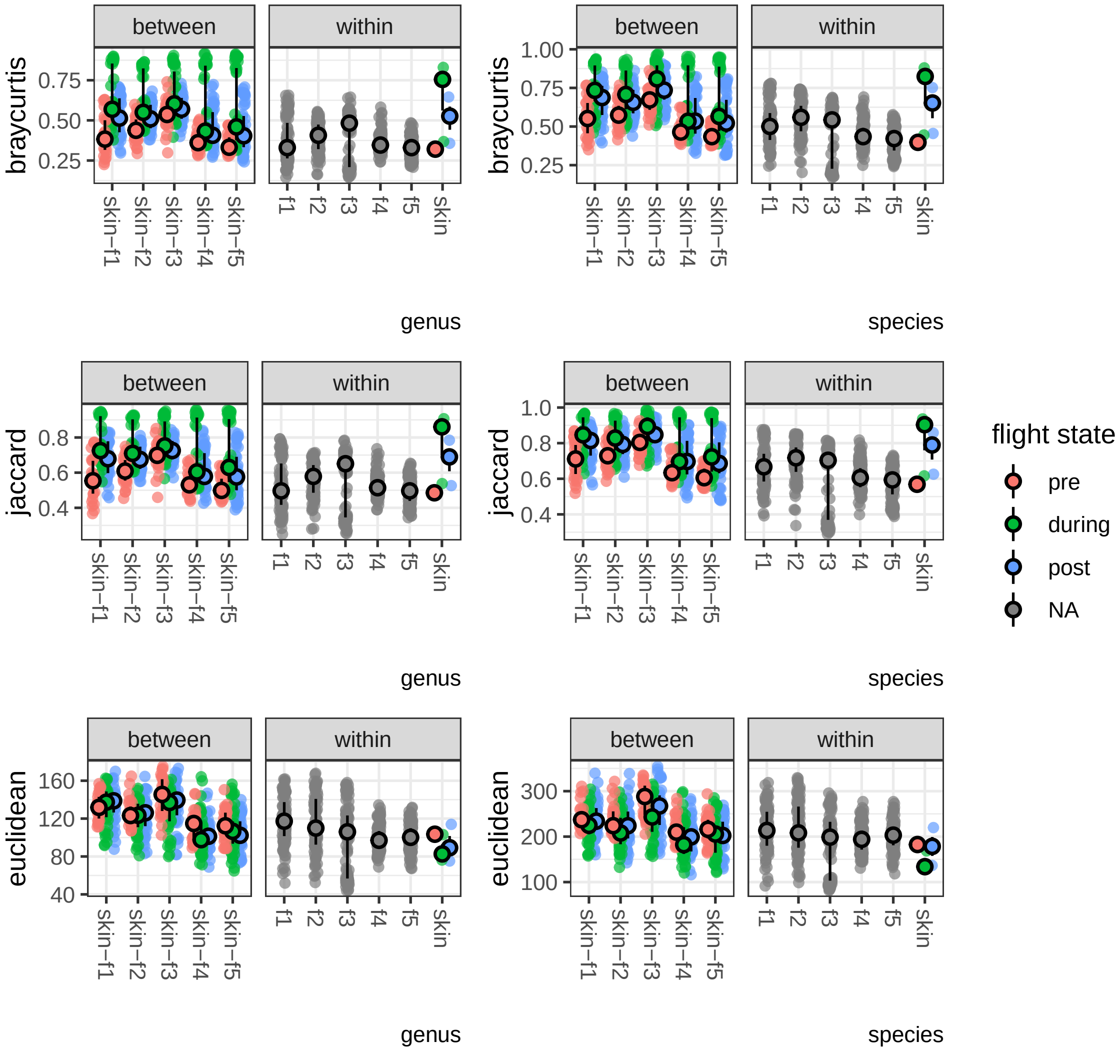

Skin-surface distances shrink from F1–3 to F4/F5 during flight. However, among pre, during, post, the during- flight distances are larger. In other words, surfaces resemble pre and post flight skin, but are different from during flight, perhaps suggesting shedding.

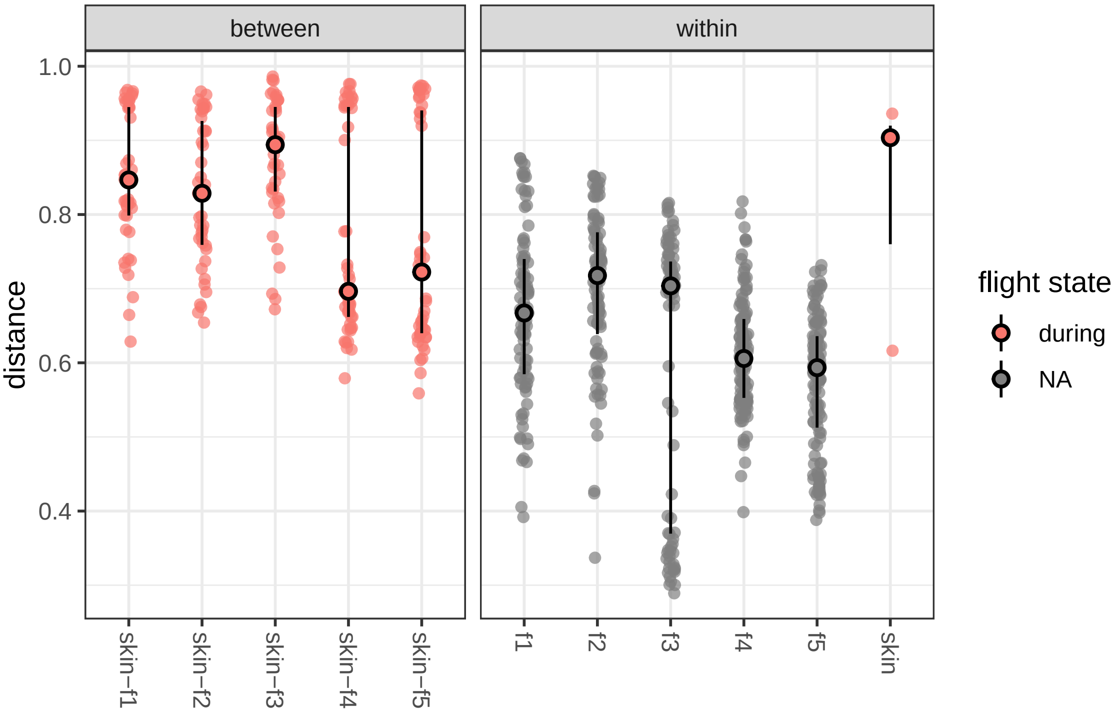

statistic	p.value	parameter	method
23.76938	8.88e-05	4	Kruskal-Wallis rank sum test

5.2.2 Flight 4 surfaces-bodysite Distances

Similarly, we compare within and between group distances for Flight 4 surfaces vs crewmember.

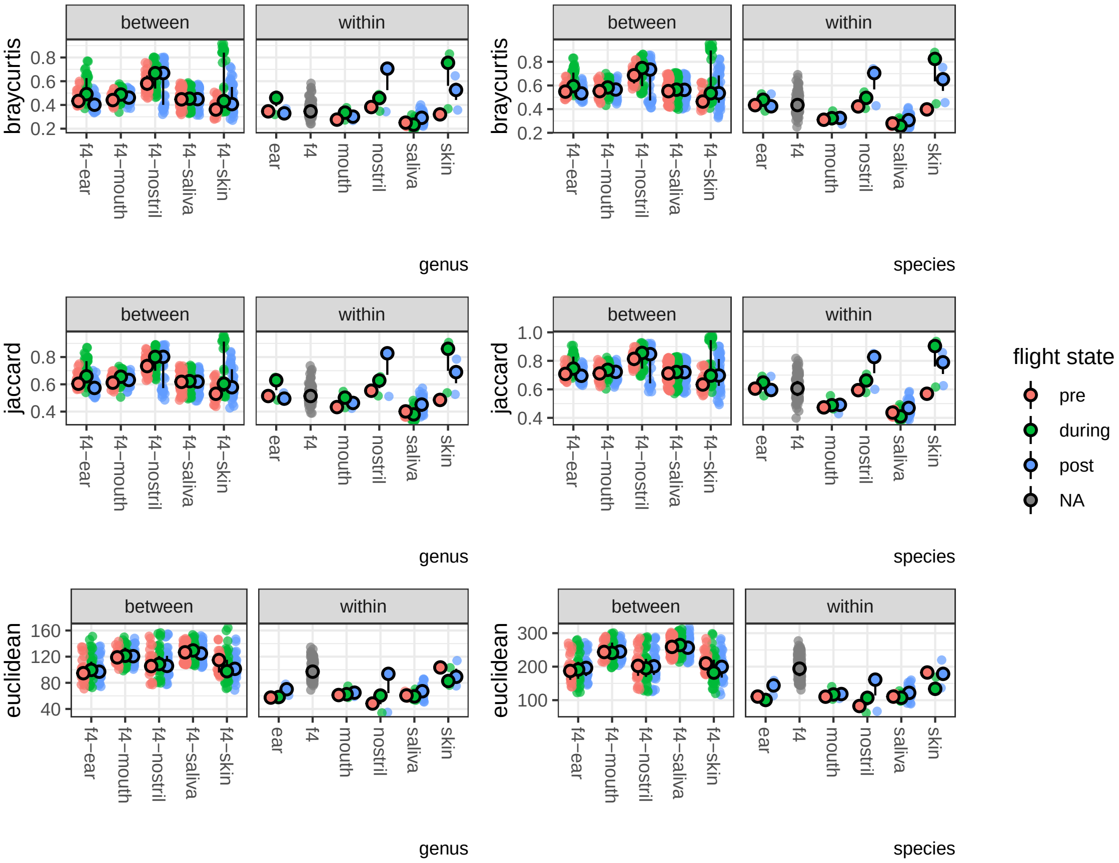

Pre-flight skin is the closest bodysite to F4. Though, there is a spike of different samples during flight.

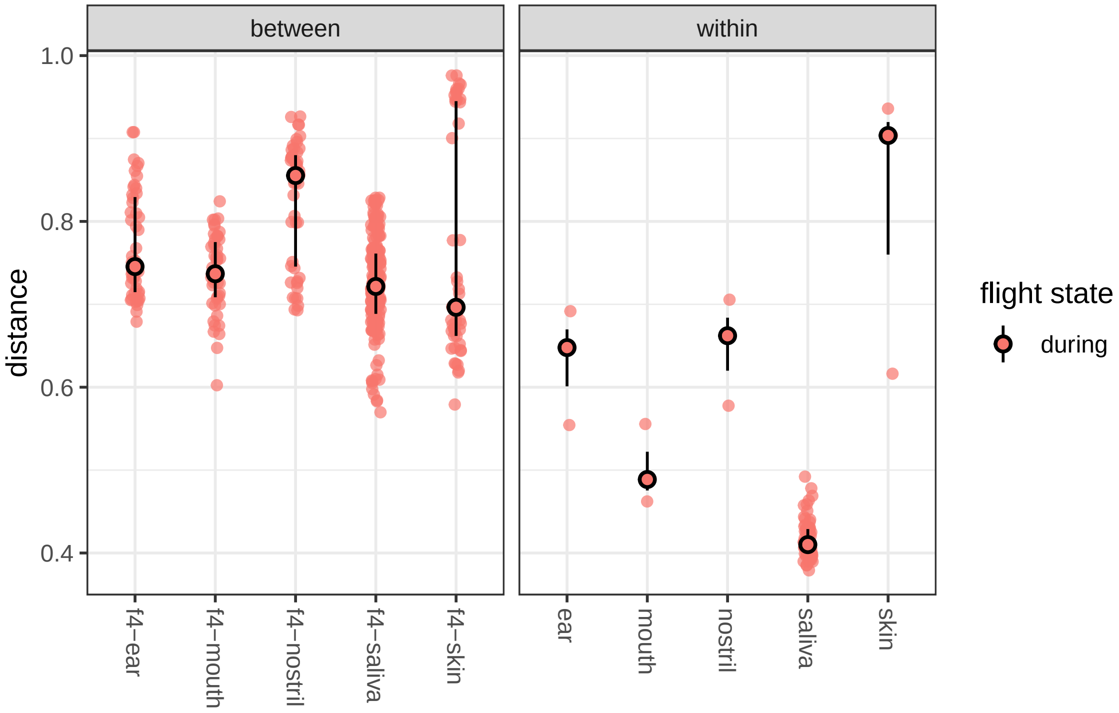

statistic	p.value	parameter	method
55.8157	0	4	Kruskal-Wallis rank sum test

5.2.3 Jaccard - NMDS

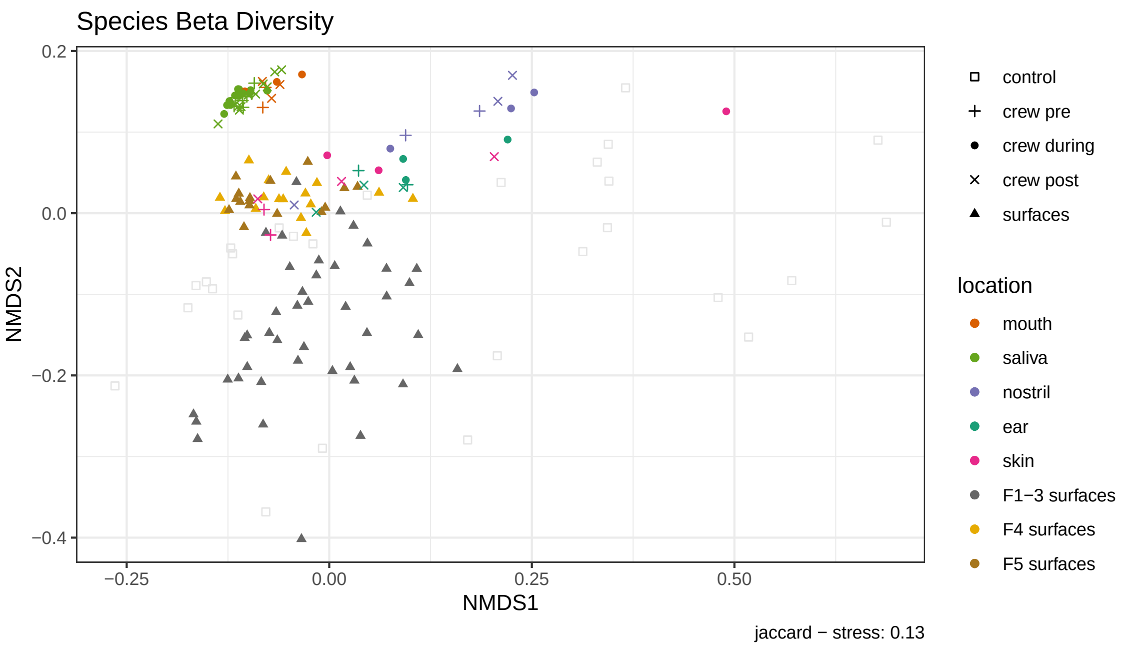

5.2.4 Euclidean - PCoA

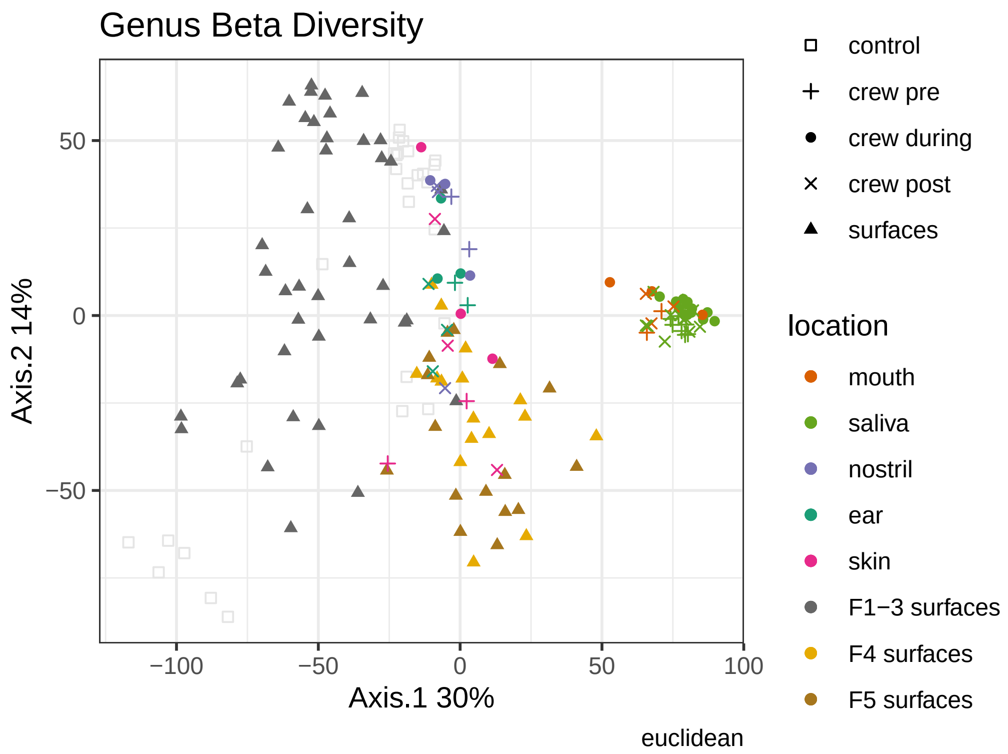

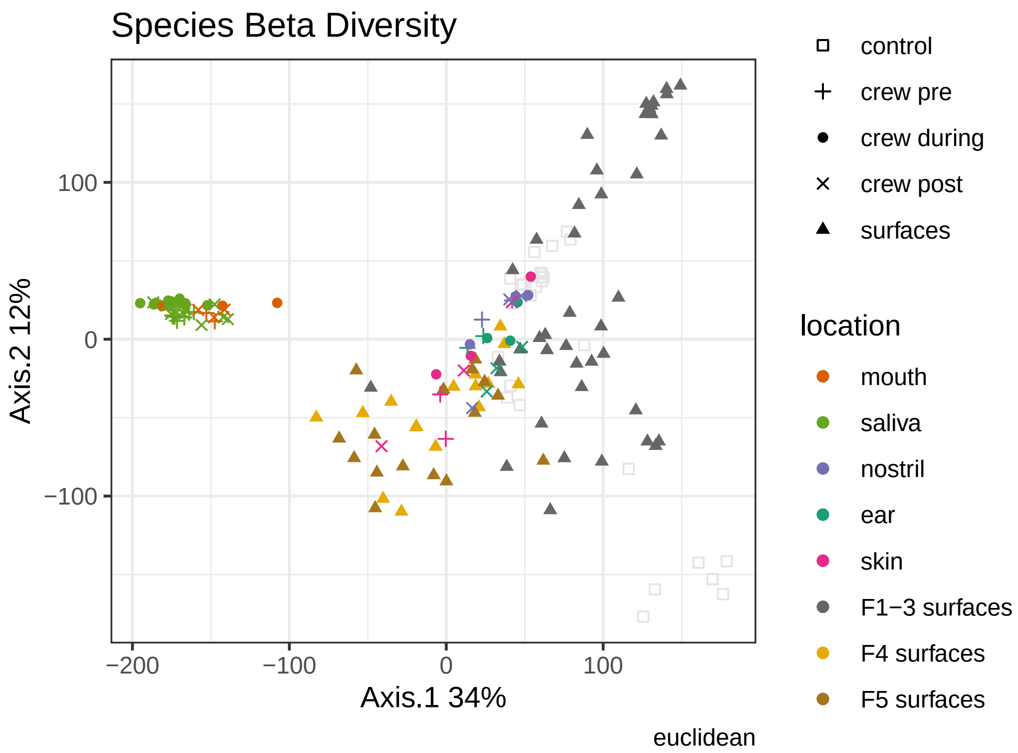

5.3 PERMANOVA

Permutational multivariate analysis of variance using distance matrices (`adonis2`).

Taxa may "prefer" one or another type of sample. We can test for this by checking if sample-sample distances (e.g., the distances used for the ordination) are larger between sample types than within sample types.

Distances between samples are larger the fewer taxa they share (or the more different their compositions are). And sample types can be defined as any grouping of samples.

5.3.1 Details

PERMANOVA compares the sum of squared distances within groups, among groups (i.e., between group centroids), and over the whole data set. The null hypothesis is that differences within groups are equal to or larger than among groups. `adonis2` allows for complicated designs such as when groups are nested, interact, and can include continuous variables as predictors.

The test statistic is a pseudo-F statistic which is not distributed like Fisher's F-ratio under the null hypothesis, and thus permutations are used to estimate a p-value.

The main assumption is that the observations (sample rows) are exchangeable under the null hypothesis, i.e., observations are independent and have similar dispersions of points (similarly distributed abundances of taxon columns).

The test is a location test, but has been shown to confound location and dispersion [2]. In other words, something might be "significant", but could result from lower variance in one group vs another. Variance and mean abundances are related in most data sets. But maybe not too much [3]

5.3.2 Model and data set up

We are interested in whether the apparent groupings are measurable, e.g., do Flight 4 surfaces cluster with Crew skin samples? Therefore, we partition our samples into a few more groups

- f1–3, f4, and f5 surfaces; oral, skin, ear/nose
- oral vs not oral
- f4 vs not f4
- skin and f4 surfaces vs not…
- skin and f4, f5 surfaces vs not…

Furthermore, we drop pma-treated samples and two Crew 1 skin samples with poor library peaks. See Jimmy's email (Thurs Oct 25, 2018) and `20181024_NASA_ISS_surface_crew_QC_readCounts.xlsx`

```
pmva_samples <- the_samples$genus %>%
  select(-lmat) %>%
  mutate(
    visgrp = case_when(
      experiment == "crew" & location %in% c("mouth", "saliva") ~ "oral",
      experiment == "crew" & location %in% c("ear", "nostril") ~ "earnose",
      experiment == "crew" ~ location, # skin
      study == "MT1" ~ study,
      study == "MT2" ~ flight_group
    ),
    is_oral = visgrp == "oral",
    is_f4 = flight_group == "f4",
    is_skin_f4surf = location == "skin" |
      (experiment == "surfaces" & is_f4),
    is_skin_mt2surf = location == "skin" |
      (experiment == "surfaces" & study == "MT2"),
    fsloc = ifelse(experiment == "crew", flight_status, location)
  )

pmva_samples <- pmva_samples %>%
  filter(
    pma_treated == "no",
    !(sample %in% c("S1_R+1_Pool", "S1+R-9_Pool")), # no library peak
    sample != "F4_4S", # red in xlsx
  )

pmva_otu <- prep_pmva(pmva_samples)
```

```
#> Joining, by = "sample"
#> Joining, by = "sample"
```

5.3.3 Visible groups

The NMDS plot shows samples may group into oral, ear/nose, skin, flights 1–3, flight 4, flight 5.

```
grps_rhs <- ~ sum_reads + visgrp
pmva_visgrps <- pmva_otu %>%
```

Table 5.1: sum_reads + visgrp

tax_rank	abundtype	term	df	SumOfSqs	R2	statistic	p.value
genus	read_count	sum_reads	1	4.038584e-01	0.0195480	1.967348	0.030
		visgrp	5	1.575424e+00	0.0762555	1.534898	0.010
		Residual	91	1.868054e+01	0.9041965	NA	NA
		Total	97	2.065982e+01	1.0000000	NA	NA
	clr_zero	sum_reads	1	1.487514e+04	0.0204641	2.113292	0.048
		visgrp	5	7.147785e+04	0.0983341	2.030954	0.003
		Residual	91	6.405348e+05	0.8812018	NA	NA
		Total	97	7.268878e+05	1.0000000	NA	NA
species	read_count	sum_reads	1	4.616192e-01	0.0179808	1.819165	0.051
		visgrp	5	2.119762e+00	0.0825680	1.670727	0.003
		Residual	91	2.309155e+01	0.8994513	NA	NA
		Total	97	2.567294e+01	1.0000000	NA	NA
	clr_zero	sum_reads	1	5.958969e+04	0.0209714	2.190899	0.049
		visgrp	5	3.068029e+05	0.1079730	2.256009	0.002
		Residual	91	2.475085e+06	0.8710556	NA	NA
		Total	97	2.841478e+06	1.0000000	NA	NA

```
  do_adonis(grps_rhs, data = pmva_samples, by = "terms", permutations = 999, parall
  select(tax_rank, abundtype, tdy) %>%
  unnest(tdy)

pmva_visgrps %>%
  kable(caption = fmlacap(grps_rhs)) %>%
  kable_styling_scale() %>%
  collapse_rows(1:2)
```

```
pmva_visgrps %>% write_tsv("results/permanova_visiblegroups_sequential.tsv")
```

5.3.4 Surfaces only: location or Flight

Do environmental surface samples separate by flight group or by location after accounting for each other term?

```
locfgr_rhs <- ~ sum_reads + flight_group + location
pmva_surfsamples <- filter(pmva_samples, experiment == "surfaces")
pmva_surfotu <- prep_pmva(pmva_surfsamples)
```

```
#> Joining, by = "sample"
#> Joining, by = "sample"
```

```
pmva_locfgr <- pmva_surfotu %>%
  do_adonis(locfgr_rhs, data = pmva_surfsamples, by = "margin", permutations = 999, parallel =
  select(tax_rank, abundtype, tdy) %>%
  unnest(tdy)

pmva_locfgr %>%
  kable(caption = fmlacap(locfgr_rhs)) %>%
  kable_styling_scale() %>%
  collapse_rows(1:2)
```

```
pmva_locfgr %>% write_tsv("results/permanova_location_flightgroup_marginal.tsv")
```

5.3.5 Crew only: body site or flight state

Do environmental surface samples separate by body site or by flight state (`flight_status`) after accounting for each other term?

```
bdyfls_rhs <- ~ sum_reads + flight_status + location
pmva_crewsamples <- filter(pmva_samples, experiment == "crew")
pmva_crewotu <- prep_pmva(pmva_crewsamples)
```

```
#> Joining, by = "sample"
#> Joining, by = "sample"
```

```
pmva_bdyfls <- pmva_crewotu %>%
  do_adonis(bdyfls_rhs, data = pmva_crewsamples, by = "margin", permutations = 999, parallel =
  select(tax_rank, abundtype, tdy) %>%
  unnest(tdy)

pmva_bdyfls %>%
  kable(caption = fmlacap(bdyfls_rhs)) %>%
  kable_styling_scale() %>%
  collapse_rows(1:2)
```

Table 5.2: sum_reads + flight_group + location

tax_rank	abundtype	term	df	SumOfSqs	R2	statistic	p.value
genus	read_count	sum_reads	1	1.787626e-01	0.0283828	1.2941922	0.196
		flight_group	4	1.903673e+00	0.3022535	3.4455179	0.001
		location	7	1.050665e+00	0.1668181	1.0866468	0.267
		Residual	23	3.176916e+00	0.5044111	NA	NA
		Total	35	6.298267e+00	1.0000000	NA	NA
	clr_zero	sum_reads	1	1.016205e+04	0.0327383	1.6108048	0.117
		flight_group	4	1.100184e+05	0.3544374	4.3598031	0.001
		location	7	4.896659e+04	0.1577517	1.1088257	0.262
		Residual	23	1.450997e+05	0.4674558	NA	NA
		Total	35	3.104030e+05	1.0000000	NA	NA
species	read_count	sum_reads	1	2.421073e-01	0.0291354	1.3182083	0.179
		flight_group	4	2.523884e+00	0.3037264	3.4354652	0.001
		location	7	1.364066e+00	0.1641528	1.0609952	0.328
		Residual	23	4.224270e+00	0.5083523	NA	NA
		Total	35	8.309730e+00	1.0000000	NA	NA
	clr_zero	sum_reads	1	3.702087e+04	0.0316456	1.5418144	0.128
		flight_group	4	4.338244e+05	0.3708353	4.5168897	0.001
		location	7	1.654215e+05	0.1414031	0.9841909	0.516
		Residual	23	5.522584e+05	0.4720733	NA	NA
		Total	35	1.169857e+06	1.0000000	NA	NA

Table 5.3: sum_reads + flight_status + location

tax_rank	abundtype	term	df	SumOfSqs	R2	statistic	p.value
genus	read_count	sum_reads	1	1.492103e-01	0.0125320	0.9072579	0.447
		flight_status	2	4.225660e-01	0.0354907	1.2846846	0.152
		location	4	1.787164e+00	0.1501014	2.7166673	0.001
		Residual	54	8.880998e+00	0.7459023	NA	NA
		Total	61	1.190638e+01	1.0000000	NA	NA
	clr_zero	sum_reads	1	2.612681e+03	0.0090008	0.7257966	0.562
		flight_status	2	1.064266e+04	0.0366642	1.4782532	0.143
		location	4	5.505580e+04	0.1896686	3.8235928	0.001
		Residual	54	1.943861e+05	0.6696650	NA	NA
		Total	61	2.902736e+05	1.0000000	NA	NA
species	read_count	sum_reads	1	1.770639e-01	0.0123572	0.9067693	0.459
		flight_status	2	5.054934e-01	0.0352781	1.2943516	0.150
		location	4	2.222727e+00	0.1551229	2.8457247	0.001
		Residual	54	1.054452e+01	0.7358968	NA	NA
		Total	61	1.432881e+01	1.0000000	NA	NA
	clr_zero	sum_reads	1	1.064152e+04	0.0091227	0.7838672	0.443
		flight_status	2	3.845982e+04	0.0329705	1.4164988	0.181
		location	4	2.528447e+05	0.2167566	4.6562127	0.001
		Residual	54	7.330858e+05	0.6284537	NA	NA
		Total	61	1.166491e+06	1.0000000	NA	NA

```
pmva_bdyfls %>% write_tsv("results/permanova_bodysite_flightstate_marginal.tsv")
```

5.3.6 Flight 4 surfaces and skin

We test whether Flight 4 surfaces and skin samples form a group versus samples from other flights or body sites. We nest location in groups (`a/b` means `a + b %in% a` and equivalently `a + a:b`) and restrict permutations to within locations.

```
skinf4_rhs <- ~ sum_reads +
  is_skin_mt2surf / location + is_skin_f4surf / location
perm <- how(nperm = 999, blocks = pmva_samples$fsloc)
pmva_skinf4 <- pmva_otu %>%
  do_adonis(skinf4_rhs, data = pmva_samples, by = "terms", permutations = perm, par
  select(tax_rank, abundtype, tdy) %>%
  unnest(tdy)

pmva_skinf4 %>%
  kable(caption = fmlacap(skinf4_rhs)) %>%
  kable_styling_scale() %>%
  collapse_rows(columns = 1:2)
```

```
pmva_skinf4 %>% write_tsv("results/permanova_skinf4.tsv")
```

5.3.7 "One big model"

Sequentially test a bunch of grouping terms (order matters).

```
obm_rhs <- ~ sum_reads +
  experiment + study + flight_group + flight_status +
  is_oral + is_skin_mt2surf + is_skin_f4surf + location
pmva_obm <- pmva_otu %>%
  do_adonis(obm_rhs, data = pmva_samples, by = "terms", permutations = 999, paralle
  select(tax_rank, abundtype, tdy) %>%
  unnest(tdy)

pmva_obm %>%
  kable(caption = fmlacap(obm_rhs)) %>%
  kable_styling_scale() %>%
  collapse_rows(1:2)
```

Table 5.4: sum_reads + is_skin_mt2surf/location + is_skin_f4surf/lo...

tax_rank	abundtype	term	df	SumOfSqs	R2	statistic	p.value
genus	read_count	sum_reads	1	4.038584e-01	0.0195480	1.9317935	0.065
		is_skin_mt2surf	1	3.071019e-01	0.0148647	1.4689741	0.102
		is_skin_f4surf	1	1.989046e-01	0.0096276	0.9514290	0.353
		is_skin_mt2surf:location	18	4.295713e+00	0.2079259	1.1415483	0.076
		is_skin_f4surf:location	6	8.201290e-01	0.0396968	0.6538265	0.951
		Residual	70	1.463411e+01	0.7083369	NA	NA
		Total	97	2.065982e+01	1.0000000	NA	NA
	clr_zero	sum_reads	1	1.487514e+04	0.0204641	2.0000055	0.159
		is_skin_mt2surf	1	8.700234e+03	0.0119692	1.1697720	0.231
		is_skin_f4surf	1	6.882916e+03	0.0094690	0.9254283	0.386
		is_skin_mt2surf:location	18	1.562442e+05	0.2149496	1.1670829	0.041
		is_skin_f4surf:location	6	1.955700e+04	0.0269051	0.4382493	1.000
		Residual	70	5.206283e+05	0.7162430	NA	NA
		Total	97	7.268878e+05	1.0000000	NA	NA
species	read_count	sum_reads	1	4.616192e-01	0.0179808	1.7771308	0.115
		is_skin_mt2surf	1	3.814701e-01	0.0148588	1.4685744	0.091
		is_skin_f4surf	1	2.547334e-01	0.0099223	0.9806667	0.320
		is_skin_mt2surf:location	18	5.408609e+00	0.2106736	1.1567742	0.032
		is_skin_f4surf:location	6	9.836297e-01	0.0383139	0.6311257	0.973
		Residual	70	1.818287e+01	0.7082507	NA	NA
		Total	97	2.567294e+01	1.0000000	NA	NA
	clr_zero	sum_reads	1	5.958969e+04	0.0209714	2.0924832	0.193
		is_skin_mt2surf	1	3.258778e+04	0.0114686	1.1443152	0.274
		is_skin_f4surf	1	3.275258e+04	0.0115266	1.1501020	0.232
		is_skin_mt2surf:location	18	6.513973e+05	0.2292460	1.2707623	0.018
		is_skin_f4surf:location	6	7.169210e+04	0.0252306	0.4195763	0.999
		Residual	70	1.993458e+06	0.7015569	NA	NA
		Total	97	2.841478e+06	1.0000000	NA	NA

Table 5.5: sum_reads + experiment + study + flight_group + flight_s...

tax_rank	abundtype	term	df	SumOfSqs	R2	statistic	p.value
genus	read_count	sum_reads	1	4.038584e-01	0.0195480	2.0805427	0.028
		experiment	1	3.501662e-01	0.0169491	1.8039385	0.042
		study	1	5.864768e-01	0.0283873	3.0213314	0.002
		flight_group	3	8.425895e-01	0.0407840	1.4469126	0.042
		flight_status	2	8.279517e-01	0.0400755	2.1326643	0.008
		is_oral	1	1.010554e-01	0.0048914	0.5206033	0.987
		is_skin_mt2surf	1	3.912526e-01	0.0189378	2.0156016	0.024
		location	9	2.015731e+00	0.0975677	1.1538188	0.133
		Residual	78	1.514074e+01	0.7328592	NA	NA
		Total	97	2.065982e+01	1.0000000	NA	NA
	clr_zero	sum_reads	1	1.487514e+04	0.0204641	2.2624723	0.023
		experiment	1	2.025259e+04	0.0278621	3.0803708	0.008
		study	1	2.976236e+04	0.0409449	4.5267837	0.002
		flight_group	3	3.300957e+04	0.0454122	1.6735587	0.038
		flight_status	2	3.976016e+04	0.0546992	3.0237126	0.001
		is_oral	1	3.353509e+03	0.0046135	0.5100607	0.920
		is_skin_mt2surf	1	1.469828e+04	0.0202208	2.2355732	0.031
		location	9	5.834755e+04	0.0802704	0.9860580	0.510
		Residual	78	5.128286e+05	0.7055128	NA	NA
		Total	97	7.268878e+05	1.0000000	NA	NA
species	read_count	sum_reads	1	4.616192e-01	0.0179808	1.9243837	0.027
		experiment	1	5.031293e-01	0.0195977	2.0974297	0.009
		study	1	7.865744e-01	0.0306383	3.2790464	0.001
		flight_group	3	1.008822e+00	0.0392952	1.4018490	0.055
		flight_status	2	1.161912e+00	0.0452583	2.4218717	0.003
		is_oral	1	1.366689e-01	0.0053235	0.5697409	0.980
		is_skin_mt2surf	1	5.180038e-01	0.0201770	2.1594379	0.018
		location	9	2.385644e+00	0.0929245	1.1050219	0.207
		Residual	78	1.871056e+01	0.7288049	NA	NA
		Total	97	2.567294e+01	1.0000000	NA	NA
	clr_zero	sum_reads	1	5.958969e+04	0.0209714	2.3622543	0.032
		experiment	1	8.265407e+04	0.0290884	3.2765724	0.009
		study	1	1.254404e+05	0.0441462	4.9727080	0.001
		flight_group	3	1.210360e+05	0.0425961	1.5993695	0.061
		flight_status	2	1.595452e+05	0.0561487	3.1623457	0.001
		is_oral	1	1.419223e+04	0.0049947	0.5626085	0.827
		is_skin_mt2surf	1	7.460940e+04	0.0262573	2.9576657	0.015
		location	9	2.368005e+05	0.0833371	1.0430274	0.386
		Residual	78	1.967610e+06	0.6924602	NA	NA
		Total	97	2.841478e+06	1.0000000	NA	NA

```
pmva_obm %>% write_tsv("results/permanova_onebigmodel.tsv")
```

5.4 aldex.glm

We can explicitly test for differential abundance (relative to a mean) of each taxon between sample types.

ALDEx2 approaches this by fitting a glm to predict abundance from sample conditions (clr-transformed abundances). ALDEx2 first generates a distribution of abundances by sampling from a dirichlet posterior (adding a 0.5 pseudo count to observed counts to estimate the concentration hyperparameter).

ALDEx2 fits the glm and tests the sample type coefficients for significance for each montecarlo instance, adjusts the p-values for multiple testing, and finally averages the p-values to report an expected p-value for each taxon.

`aldex.glm` also performs a Kruskal-Wallis test. This is a non-parametric test which compares two or more samples of potentially multiple sizes. It is similar to the Mann-Whitney U-test, and tests if at least one sample is drawn from a different distribution than the rest.

Basically,

```
for each mc instance m:
  for each taxon t:
    fits <- glm(clr ~ factor(conditions))
    glm_pvalue[m,t] <- drop1(fits, test = "Chis")
    kw_pvalues[m,t] <- kruskal.test(clr, factor(conditions))
  glm_padj[m,] <- p.adjust(glm_pvalues[m,])
  kw_padj[m,] <- p.adjust(kw_pvalues[m,])
for each taxon t:
  average(glm_pvalues[,t])
  average(glm_padj[,t])
  average(kw_pvalues[,t])
  average(kw_padj[,t])
```

5.5 Saliva by flight state

This next part should take 3–5 hours. Set `mc.max` to 2 for debugging.

Plot previously computed data.

```
#> Joining, by = "genus"
```

```
#> Joining, by = "species"
```

```
#> Joining, by = c("study", "sample", "flight_group", "swab_location_code", "pma_tr
```

```
#> Joining, by = c("study", "sample", "flight_group", "swab_location_code", "pma_tr
```

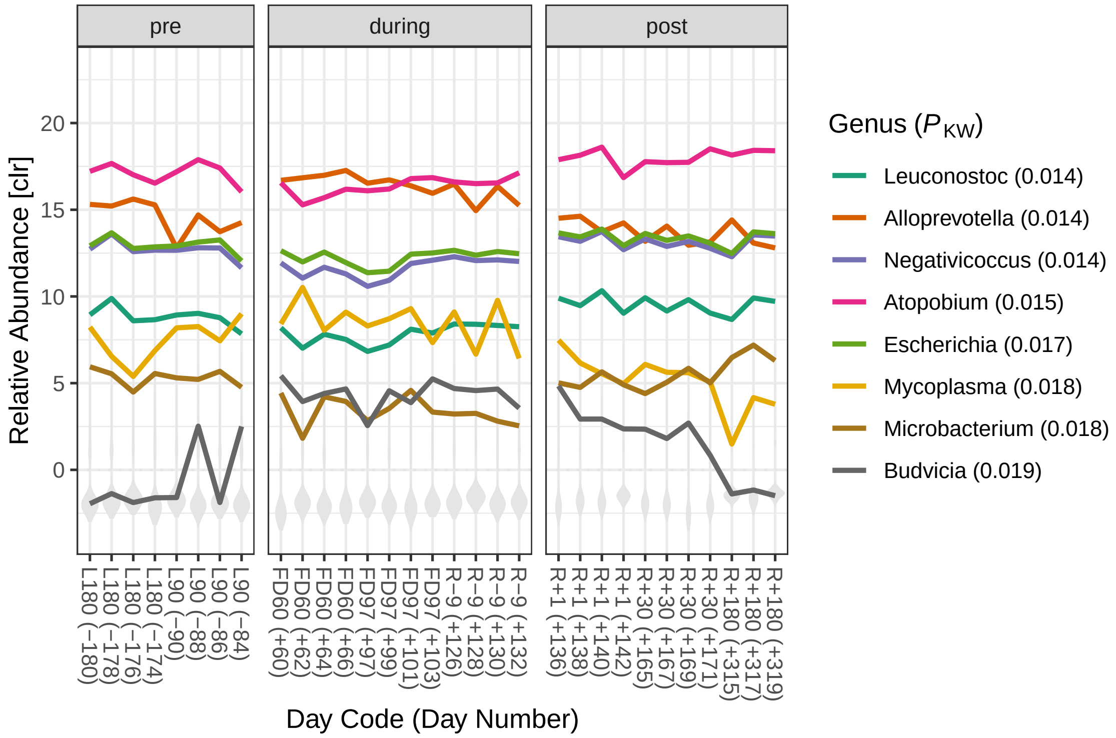

saliva genus

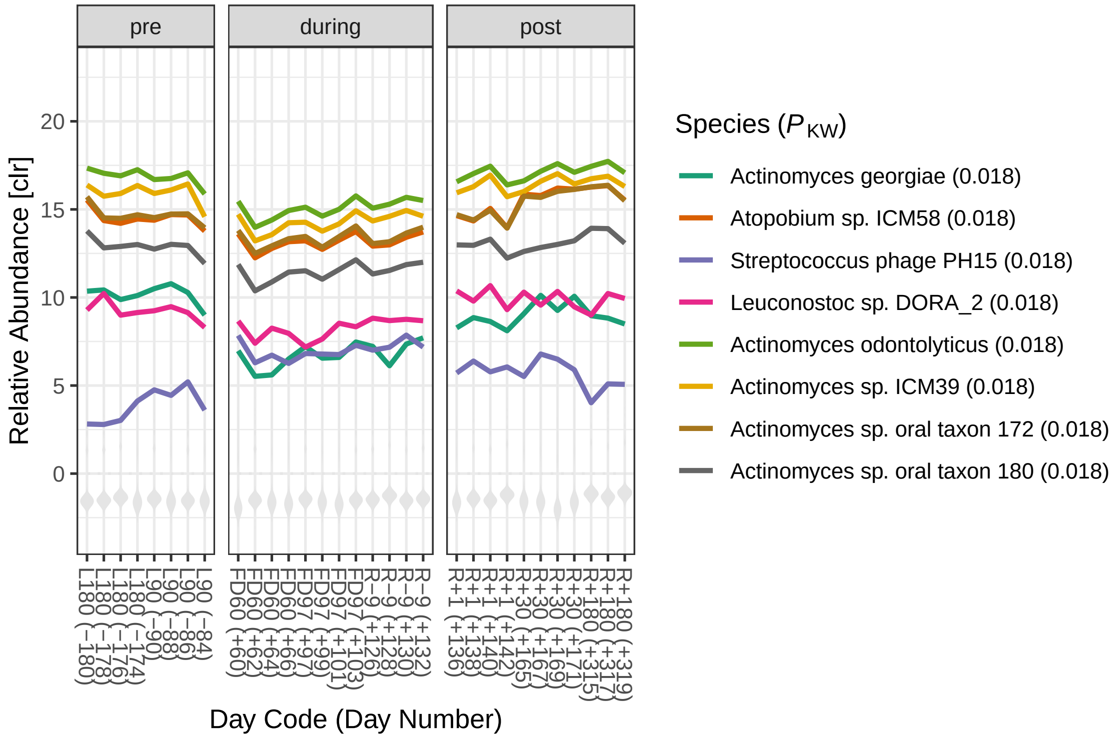

saliva species

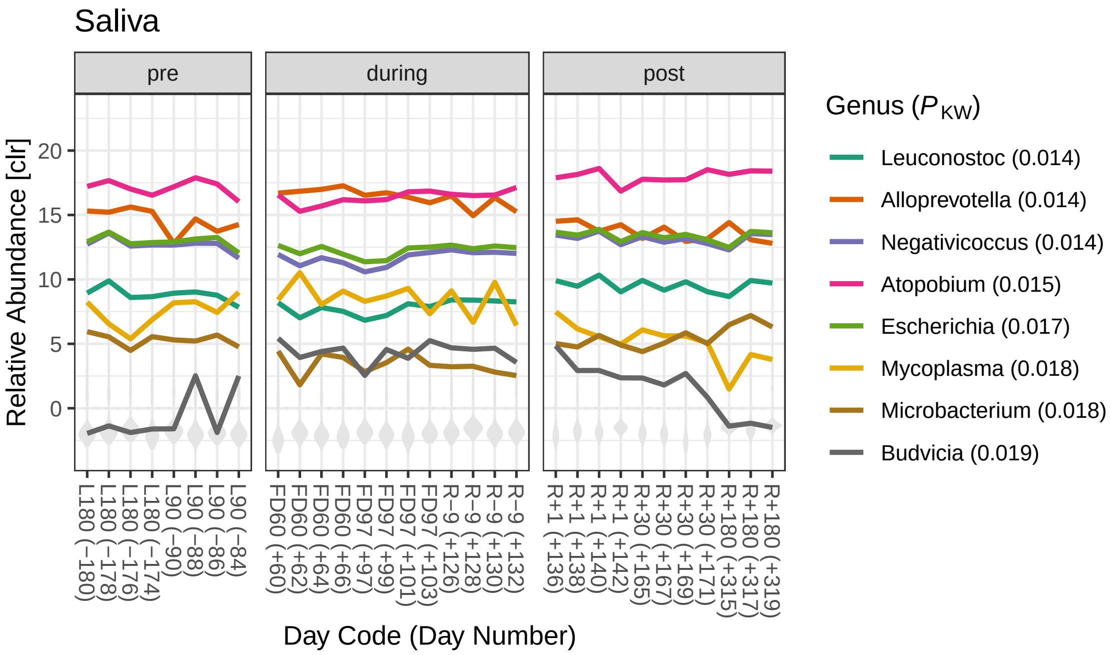

genus

5.6 "Venn" (Euler) Diagrams

Euler diagrams are another way to summarize taxa shared by multiple conditions or locations.

The area of the overlaps is proportional to the number of shared vs total taxa in each condtion. For these figures, a taxon need only be seen in one sample for it to be considered present for the particular condition it was sampled from.

5.6.1 Saliva

Shared taxa among pre, during, post flight in saliva.

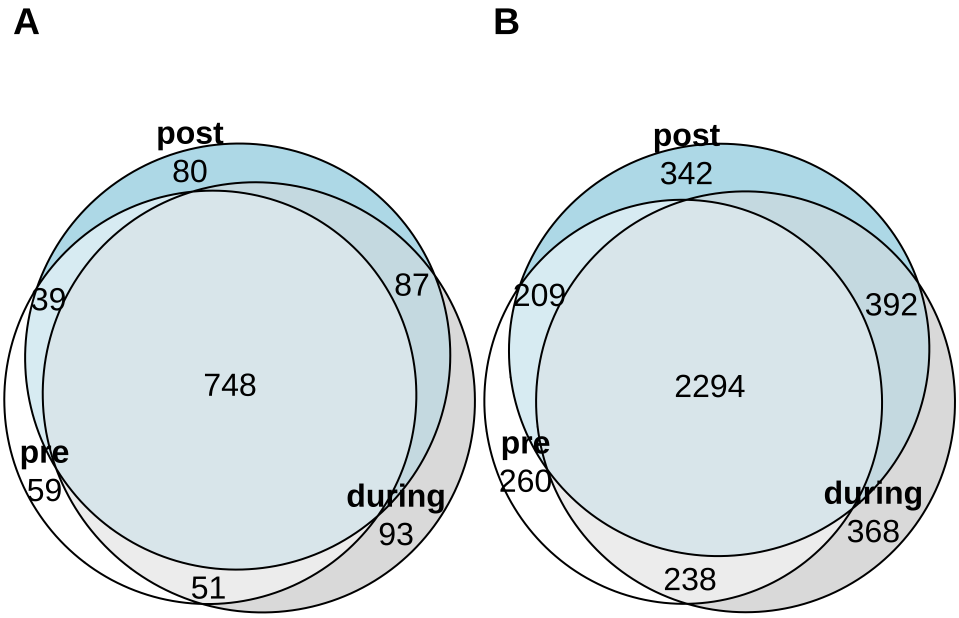

5.6.2 Skin

Skin vs surfaces across flight groups (no PMA)

```
#> Warning in do_euler(.x, reformulate("set", .y)): probably a bad fit, quantities
#> and areas may be inaccurate

#> Warning in do_euler(.x, reformulate("set", .y)): probably a bad fit, quantities
#> and areas may be inaccurate
```

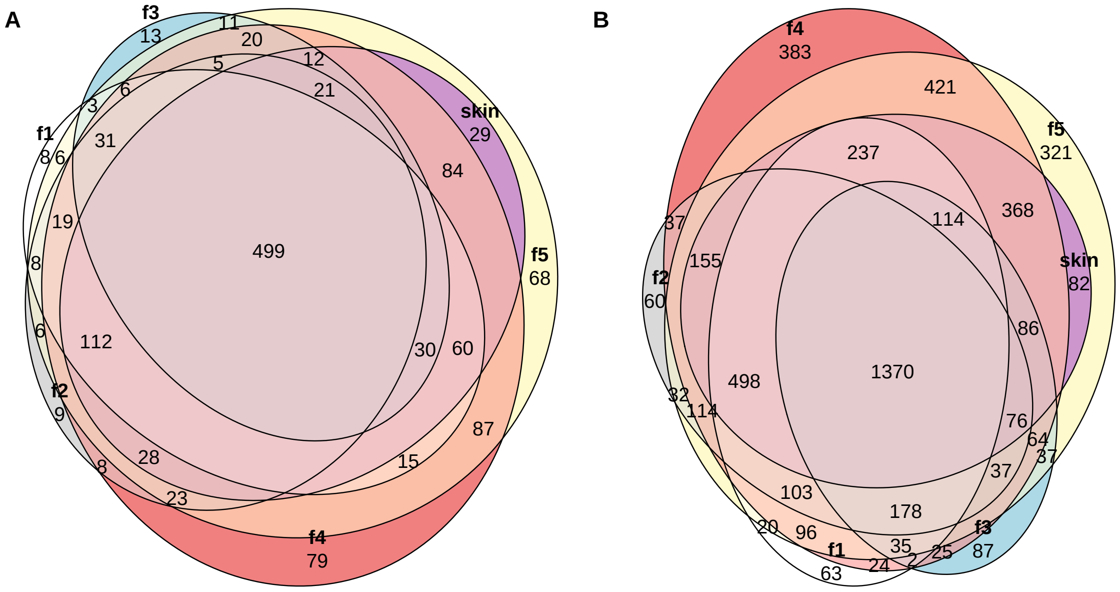

5.6.2.1 F4, F5, and skin only

Skin vs surfaces across F4 and F5 only (no PMA)

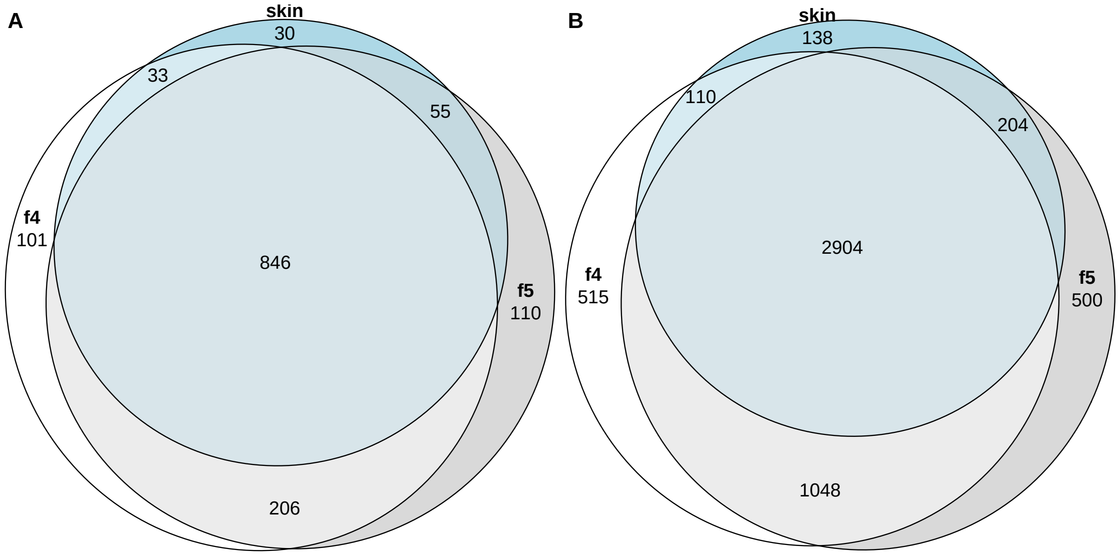

5.6.3 F4 vs Skin pre, during, post

Flight 4 surfaces and pre, during, post flight

tax_rank	flight_status	numer	denom	frac
genus	pre	761	797	0.9548306
	during	430	437	0.9839817
	post	664	690	0.9623188
species	pre	2350	2504	0.9384984
	during	1348	1389	0.9704824
	post	2234	2361	0.9462092

5.6.4 Surfaces MT1 vs MT2

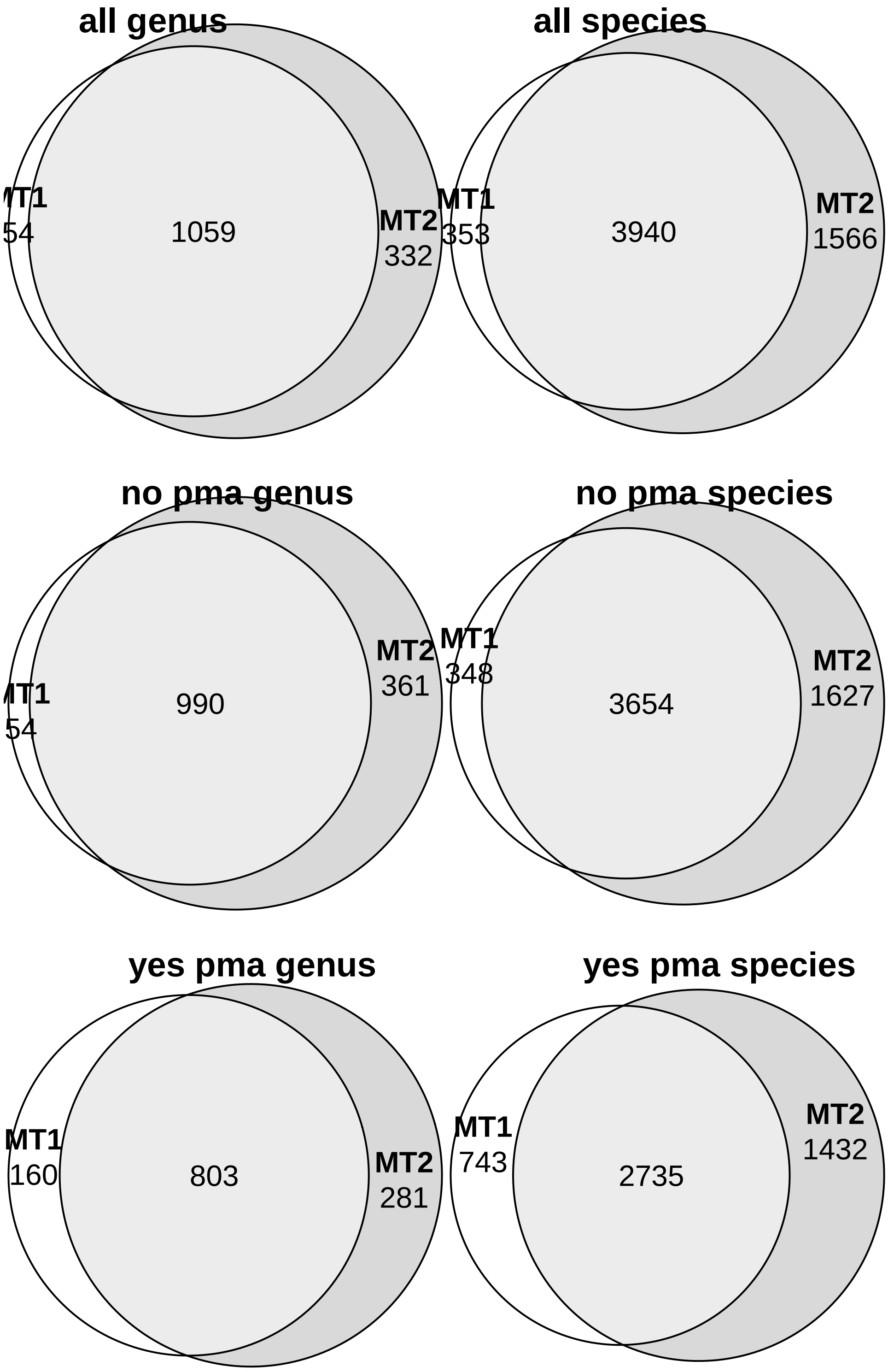

6 SourceTracker

SourceTracker results by Camilla Urbaniak camilla.urbaniak@jpl.nasa.gov

Tests if microbial contribution from crewmember is different between flights 4 and 5. (the crewmember is "S1"; flights 4 and 5 are "f4", "f5")

1. Loads manually created table with values copied from email from Camilla Urbaniak (subj: "Re: [EXTERNAL] Re: stats", date: June 15, 2019).
2. Treats average proportion as point estimates (instead of 10 tight samples).
3. t-test: Null hypothesis is that the mean S1 proportion in F4 is the same as the mean S1 proportion in F5.

```
#> Parsed with column specification:
#> cols(
#>   flight_group = col_character(),
#>   swab_location_code = col_double(),
#>   S1_mean = col_double(),
#>   S1_sd = col_double(),
#>   Unknown_mean = col_double(),
#>   Unknown_sd = col_double()
#> )
```

```
#> Joining, by = "swab_location_code"
```

```
#> # A tibble: 4 x 7
#>   stat         minimum    q1 median    mean    q3 maximum
#>   <chr>          <dbl> <dbl>  <dbl>   <dbl> <dbl>   <dbl>
#> 1 S1_mean         0     0      0.02 0.221    0.39    0.89
#> 2 S1_sd           0     0      0    0.00459  0.01    0.01
#> 3 Unknown_mean    0.11  0.61   0.98 0.779    1       1
#> 4 Unknown_sd      0     0      0    0.00459  0.01    0.01
```

Standard deviations for sample distributions are small compared to the overall spread, so use means as point estimates.

flight_group	mean	var	sd	se	n
f1	0.0371429	0.0058905	0.0767494	0.0290086	7
f2	0.0157143	0.0010952	0.0330944	0.0125085	7
f3	0.0014286	0.0000143	0.0037796	0.0014286	7
f4	0.5537500	0.0854268	0.2922786	0.1033361	8
f5	0.4187500	0.0164982	0.1284454	0.0454123	8

6.1 F4 vs F5

estimate	statistic	p.value	parameter	conf.low	conf.high	method	alternative
0.135	1.188938	0.2732277	7	-0.1334953	0.4034953	Paired t-test	two.sided

6.1.1 Plot

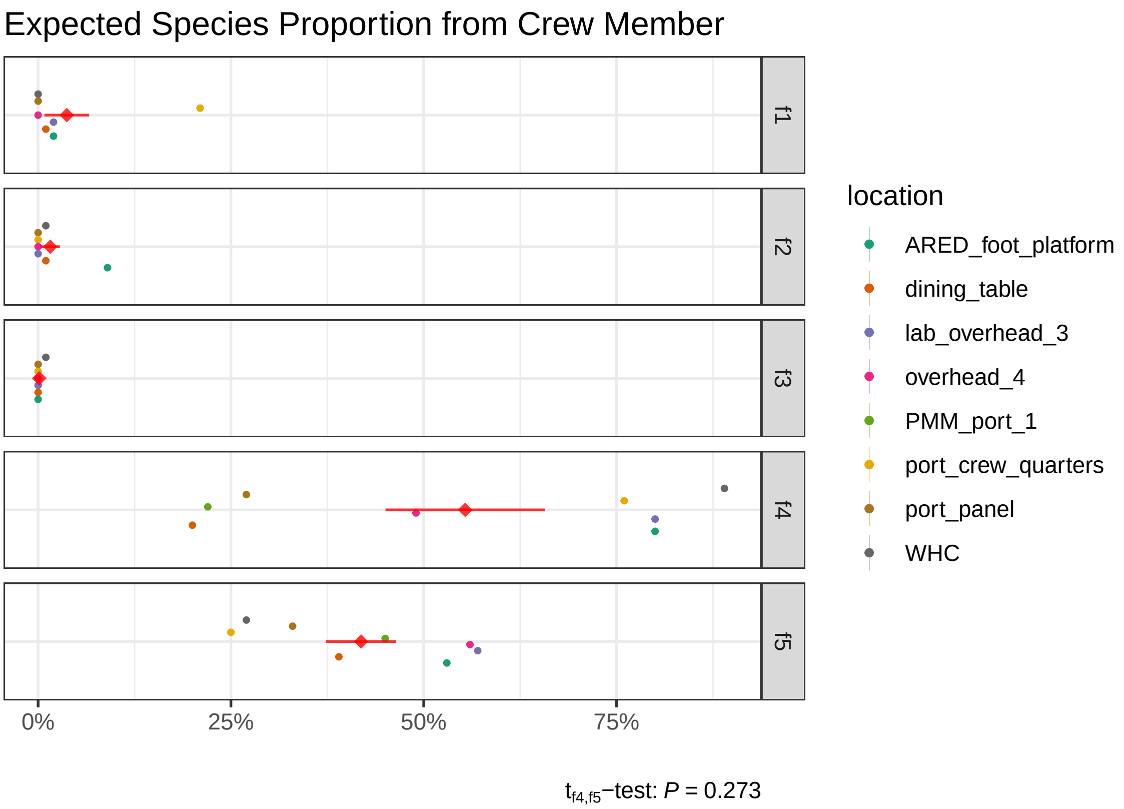

1. Hill MO. Diversity and Evenness: A Unifying Notation and Its Consequences. Ecology. 1973;54: 427–432. doi:10.2307/1934352

2. Warton DI, Wright ST, Wang Y. Distance-based multivariate analyses confound location and dispersion effects: Mean-variance confounding in multivariate analysis. Methods in Ecology and Evolution. 2012;3: 89–101. doi:10.1111/j.2041-210X.2011.00127.x

3. Anderson MJ. Permutational Multivariate Analysis of Variance (PERMANOVA). In: Balakrishnan N, Colton T, Everitt B, Piegorsch W, Ruggeri F, Teugels JL, editors. Wiley StatsRef: Statistics Reference Online. Chichester, UK: John Wiley & Sons, Ltd; 2017. pp. 1–15. doi:10.1002/9781118445112.stat07841
